# Clinical Benefit of PD‐1/PD‐L1 Inhibitors as Standard First‐Line Treatment in Low PD‐L1‐Expressing Advanced Solid Tumors: A Systematic Review and Meta‐Analysis

**DOI:** 10.1002/mco2.70624

**Published:** 2026-02-09

**Authors:** Peng Wu, Xuanyu Gu, Dongyu Li, Xiaohui Zi, Dexin Shang, Jingjing Liu, Ruijie Ma, Jilin Peng, Guochao Zhang, Yun Che, Qingpeng Zeng, Bohui Zhao, Nan Sun, Chaoqi Zhang, Jie He

**Affiliations:** ^1^ Department of Thoracic Surgery, National Cancer Center/National Clinical Research Center For Cancer/ Cancer Hospital Chinese Academy of Medical Sciences and Peking Union Medical College Beijing China; ^2^ Department of Otolaryngology‐Head and Neck Surgery The First Affiliated Hospital of Zhengzhou University Zhengzhou China

**Keywords:** advanced solid tumors, individual patient data, low pd‐l1 expression, meta‐analysis, programmed death‐1/programmed death‐ligand 1 (pd‐1/pd‐l1) inhibitors, survival benefit

## Abstract

This study aimed to assess the benefits of immune checkpoint inhibitors (ICIs) for patients with low or negative PD‐L1 expression in advanced solid tumors. The study included cancers approved by the FDA for first‐line ICI therapy without PD‐L1 restrictions, incorporating phase III randomized clinical trials (RCTs) comparing immunotherapy with conventional care. Individual patient data of PD‐L1 low subgroup were retrieved from Kaplan–Meier (KM) curves using IPDfromKM and KMSubtraction. Pooled analysis employed KM and restricted mean survival time (RMST) analysis to assess ICI benefit. Totally, 40 RCTs with 27,060 patients were enrolled. No survival benefit for low PD‐L1 expression was observed in some cancers. In esophageal squamous cell carcinoma (ESCC), combined positive score (CPS) < 10 had significant OS benefit (HR = 0.82, *p* = 0.02; RMST‐D = 2.34 months); tumor proportion score (TPS) < 1% showed no OS improvement (HR = 0.87, *p* = 0.16, RMST‐D = 1.71 months). Human epidermal growth factor receptor 2 (HER2)‐negative gastroesophageal adenocarcinoma (GEA) had no OS benefit with CPS < 5, 1–4, and < 1, but significant benefits with CPS < 10 (HR = 0.87, *p* = 0.048; RMST‐D = 1.78 months, *p* = 0.038) and CPS 1–9 (HR = 0.83, *p* = 0.0085; RMST‐D = 2.21 months, *p* = 0.007). Patient‐level data indicate that ESCCs with TPS < 1% and HER2‐negative GEAs with CPS < 5 do not benefit from the addition of ICIs to conventional chemotherapy. More nuanced clinical trials and predictive biomarkers are warranted.

## Graphical abstract

This is the first meta‐analysis to focus on the actual clinical benefit of ICIs as standard first‐line therapy for multiple cancers with low PD‐L1 expression, in accordance with current FDA guidelines. The mirror‐based study design enhances precision and accuracy, while ensuring alignment with clinical practice. To our knowledge, our patient‐level analysis provides first evidence that, contrary to FDA guidelines, there is no clinical benefit from the addition of ICIs to conventional chemotherapy as first‐line treatment for HER2‐negative GEAs with CPS <5. Several rigorous algorithms were seamlessly integrated to enhance precision and statistical efficacy in this study. Two well‐known methods, graphical reconstruction and KMSubtraction, were implemented to ensure that individual data more closely approximated the raw data. Additionally, RMST was incorporated as a robust, nonparametric complement to the log‐rank‐based hazard ratio; this metric can be intuitively interpreted as an absolute gain (or loss) of survival time.

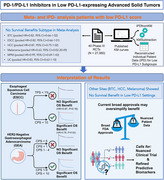

## 1 Introduction

The incidence and mortality rates of cancer have remained high in recent years, posing a serious global public health problem [1]. In the past decades, with the identification of programmed cell death 1 (PD‐1) and programmed death ligand 1 (PD‐L1) as key molecules, which promote tumor immune evasion and anti‐tumor response, immune checkpoint inhibitors (ICIs) targeting the PD‐1/PD‐L1 pathway have garnered significant attention for their effectiveness in treating a range of cancers [2]. The programmed cell death 1 receptor, expressed on activated T cells, and its ligand (PD‐L1), expressed on tumor cells and antigen‐presenting cells, are central to regulating immune responses. Under normal conditions, the interaction between PD‐1 and PD‐L1 downregulates T‐cell activation, preventing excessive immune responses that could lead to autoimmunity. However, many tumors exploit this pathway by upregulating PD‐L1 expression, which allows them to escape immune surveillance [2]. Blocking the PD‐1 receptor with antibodies, such as nivolumab and pembrolizumab, or inhibiting PD‐L1 with agents like atezolizumab and durvalumab reactivates T cells and enhances their ability to recognize and attack tumor cells [2].

In clinical practice, anti‐PD‐1 and anti‐PD‐L1 therapies have demonstrated significant efficacy in a variety of malignancies, including melanoma, non‐small cell lung cancer (NSCLC), renal cell carcinoma (RCC), and urothelial carcinoma. The emergence of these agents dramatically transformed the cancer management landscape and extended patient survival [3]. Furthermore, the combination of anti‐PD‐1/PD‐L1 agents with other treatment modalities, such as chemotherapy, targeted therapies, or other immunotherapies, has shown promise in overcoming resistance and improving outcomes. Therefore, ICI‐based therapies have been recommended as first‐line treatments for most cancers by authoritative guidelines worldwide [3].

As the most influential drug regulatory agencies worldwide, the US Food and Drug Administration [4] (FDA) and European Medicines Agency [5] (EMA) have both approved the use of ICIs for patients with specific programmed death ligand 1 expression statuses. For most indications, the FDA and EMA guidelines are consistent regarding PD‐L1 score cutoff values. However, discrepancies between FDA and EMA guidelines exist for some indications, such as esophageal squamous cell carcinoma (ESCC) and human epidermal growth factor receptor 2 (HER2)‐negative gastric cancer (GC). The FDA has authorized the use of pembrolizumab in combination with chemotherapy for first‐line treatment of ESCC and HER2‐negative GC, regardless of PD‐L1 status [4]. In contrast, the EMA has approved this regimen only for patients with a PD‐L1 combined positive score (CPS) ≥ 10 [5]. These discrepancies have arisen from clinical trial data suggesting that ICI therapy is not beneficial for some patients. For example, in the KEYNOTE‐590 [6] trial, which supported FDA and EMA approvals concerning the use of pembrolizumab to treat esophageal cancer, significant survival benefits were observed across all intent‐to‐treat populations. However, subgroup analysis indicated that only patients with CPS ≥ 10 experienced a survival benefit; patients with CPS < 10 did not experience this benefit. Some researchers speculated that the survival benefit among all randomly assigned patients was primarily driven by the benefit among patients with high PD‐L1 expression (CPS ≥ 10), and patients with low PD‐L1 expression may not experience a benefit [7]. This issue, observed in trials focused on other cancers, could affect regulatory approval. Therefore, the benefit of ICI therapy for cancer patients with low or negative PD‐L1 expression warrants further investigation.

Considering the lack of clinical trials designed to evaluate the efficacy of ICIs among patients with low or negative PD‐L1 expression, recent meta‐analyses [7, 8, 9] have attempted to determine survival benefits for specific cancers. However, the inclusion criteria for these meta‐analyses have differed from FDA/EMA regulatory policies, limiting their clinical relevance. Moreover, these meta‐analyses solely utilized hazard ratios (HRs) to compare drug efficacy; although HRs offer a relative measure of effect, they may not reflect the actual clinical benefit [10].

To address these limitations, we conducted a meta‐analysis of study‐level data across various cancers for which the FDA has approved ICIs without PD‐L1‐based restrictions. To obtain more granular information and comprehensively analyze ICI efficacy in patients with low PD‐L1 expression, we derived individual patient data (IPD) from Kaplan–Meier (KM) curves using the KMSubtraction method [11]. We subsequently performed a series of IPD pooled analyses to investigate ICI efficacy in subgroups with low PD‐L1 scores.

## 2 Results

**FIGURE 1:**
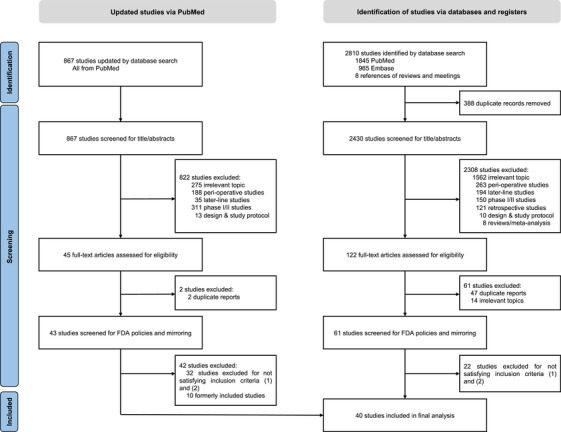
Study selection workflow.

Our database search initially identified a total of 4626 studies; of these, 171 were subjected to full‐text screening. Ultimately, 39 studies met the inclusion criteria and were included in the meta‐analysis (Figure 1). An updated search on PubMed from November 30, 2023 to August 1, 2025 was performed to ensure timeliness of this study, and one study (AK105‐302) [12] was subsequently included for analysis. Moreover, updated survival data were published after initial searching process for following studies, including CheckMate‐648 [13], ESCORT‐1^st^ [14], ORIENT‐16 [15], RATIONALE‐305 [16], ATTRACTION‐4 [17], HIMALAYA [18], TOPAZ‐1 [19], GEMSTONE‐302 [20], CheckMate‐9ER [21], and CLEAR [21], therefore data from these updated papers were retrieved for final analysis. Overall, these studies, focusing on the use of anti‐PD‐1 or anti‐PD‐L1 agents across 10 different cancers, were randomized controlled phase III trials performed in advanced or metastatic settings. The trials involved NSCLC [20, 22, 23, 24, 25, 26, 27, 28] (14 trials, 7639 patients), esophageal squamous cell carcinoma [6, 13, 14, 28, 29] (ESCC; six trials, 4339 patients), HER2‐negative gastroesophageal adenocarcinoma [15, 16, 17, 30, 31] (GEA; five trials, 5227 patients), renal cell carcinoma [21, 32, 33, 34, 35] (RCC; five trials, 3479 patients), small cell lung cancer [36, 37] (SCLC; two trials, 577 patients), hepatocellular carcinoma [18, 38] (HCC; two trials, 1712 patients), melanoma [39, 40] (two trials, 907 patients), biliary tract cancer [19, 41] (BTC; two trials, 1559 patients), malignant pleural mesothelioma [42] (MPM; one trial, 586 patients), and urothelial carcinoma (UC; one trial, 628 patients). The treatment modalities included PD‐1 inhibitors (25 trials), PD‐L1 inhibitors (13 trials), PD‐L1 and cytotoxic T‐lymphocyte associated protein 4 (CTLA‐4) inhibitors (two trials), and PD‐1 and CTLA‐4 inhibitors (two trials). Details are listed in Table S3.

**FIGURE 2:**
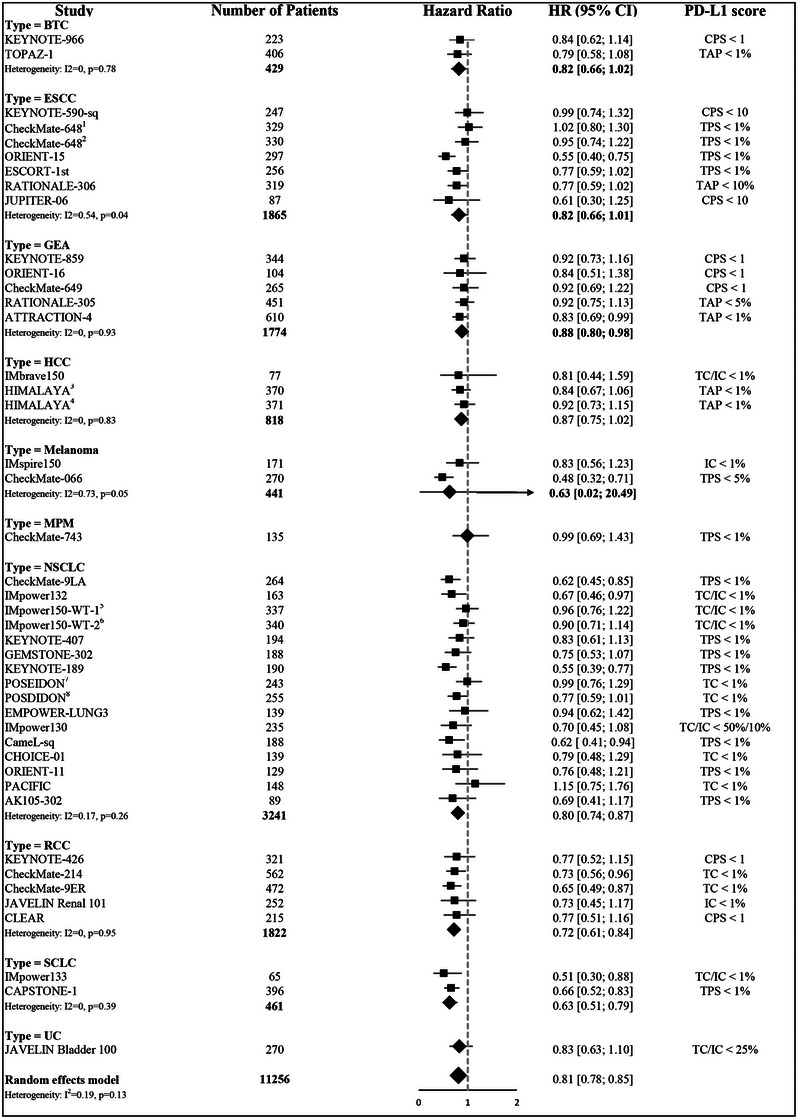
Forest plots for efficacy of immune checkpoint inhibitor in patients with low PD‐L1 score across different cancer types. BTC, biliary tract cancer; ESCC, esophageal squamous cell carcinoma; GEA, gastroesophageal adenocarcinoma, including gastric, gastro‐esophageal junction, and esophageal adenocarcinoma; HCC, hepatocellular carcinoma; MPM, malignant pleural mesothelioma; NSCLC, non‐small cell lung cancer; RCC, renal cell carcinoma; SCLC, small cell lung cancer; UC, urothelial carcinoma; PD‐L1, programmed death‐ligand 1; TPS, tumor proportion score; CPS, combined positive score; TAP, tumor area positivity. Superscript 1 indicates the hazard ratio of nivolumab + chemotherapy arm compared to chemotherapy in CheckMate 648. Superscript 2 indicates the hazard ratio of nivolumab + ipilimumab arm compared to chemotherapy in CheckMate 648. Superscript 3 indicates the hazard ratio of durvalumab + tremelimumab arm compared to sorafinib in HIMALAYA. Superscript 4 indicates the hazard ratio of durvalumab arm compared to sorafinib in HIMALAYA. Superscript 5 indicates the hazard ratio of atezolizumab + chemotherapy arm compared to bevacizumab + chemotherapy in IMpower 150. Superscript 6 indicates the hazard ratio of atezolizumab + bevacizumab + chemotherapy arm compared to bevacizumab + chemotherapy in IMpower 150. Superscript 7 indicates the hazard ratio of durvalumab + chemotherapy arm compared to chemotherapy in POSEIDON. Superscript 8 indicates the hazard ratio of durvalumab + tremelimumab + chemotherapy arm compared to chemotherapy in POSEIDON.

The PD‐L1 scoring systems used for subgroup stratification were tumor proportion score (TPS) (20 studies), CPS (eight studies), tumor cell/immune cell (TC/IC) staining (six studies), tumor area positivity (TAP) (four studies), and IC staining (two studies). Most studies reported survival outcomes based on PD‐L1 positivity (CPS > 1 or TAP/TC/TPS > 1%). However, specific cutoffs were used in some trials: KEYNOTE‐590^6^ utilized a CPS of 10, RATIONALE‐306 [43] utilized a TAP of 10%, RATIONALE‐305 [44] utilized a TAP of 5%, CheckMate 066 [40] utilized a TPS of 5%, and JAVELIN Bladder 100 [45] utilized TC/IC staining of 25% to stratify survival outcomes (reported as HRs). The characteristics of the included trials, as well as the cutoff values and staining methods for defining PD‐L1 status, are summarized in Figure 2. Figure S1 shows a detailed evaluation of the potential bias in each trial.

Generally, patients with high PD‐L1 scores tended to experience favorable overall survival (OS) benefits from ICIs (pooled HR = 0.70, 95% confidence interval [CI] = 0.66–0.74, Figure S2). Among patients with low PD‐L1 expression, significant OS benefits for chemo‐immunotherapy compared with chemotherapy alone were observed in the following subgroups (Figure 2): NSCLC (pooled HR = 0.80, 95% CI = 0.74–0.87), RCC (pooled HR = 0.72, 95% CI = 0.61–0.84), SCLC (pooled HR = 0.63, 95% CI = 0.51–0.79), and HER2‐negative GEA (pooled HR = 0.88, 95% CI = 0.80–0.98). However, survival benefits were not observed among patients with low PD‐L1‐expressing BTC (pooled HR = 0.82, 95% CI = 0.66–1.02), ESCC (pooled HR = 0.82, 95% CI = 0.66–1.01), HCC (pooled HR = 0.87, 95% CI = 0.75–1.02), melanoma (pooled HR = 0.63, 95% CI = 0.02–20.49), MPM (pooled HR = 0.99, 95% CI = 0.69–1.43), or UC (pooled HR = 0.83, 95% CI = 0.63–1.10). In the HCC and melanoma subgroups, patients did not derive survival benefits regardless of PD‐L1 status, suggesting that PD‐L1 is not an ideal biomarker for predicting survival outcomes among patients with these cancers. The MPM and UC subgroups were analyzed in one study each; thus, the statistical power was insufficient for robust conclusions. Subsequently, we conducted pooled analyses based on patient‐level data regarding patients with low PD‐L1‐expressing cancers (i.e. ESCC, BTC, and HER2‐negative GEA) who derived marginal or no survival benefits.

In total, six trials investigating ESCC (KEYNOTE‐590 [6], CheckMate‐648 [13], ORIENT‐15 [29], Jupiter‐06 [46], ESCORT‐1st [14], and RATIONALE‐306 [43]) and four trials investigating HER2‐negative GEA (CheckMate‐649 [31], KEYNOTE‐859 [30], KEYNOTE‐062 [47], and ORIENT‐16 [15]) were included in this analysis. It is worthy of noting that KEYNOTE‐062 was not included in study level meta‐analysis since this trial only enrolled patients with positive PD‐L1 expression as their intent to treat group. However, this study also reported survival outcome for CPS ≥ 1 and CPS < 10 subgroups as KM curves, making it possible to derive further information for IPD analysis. Regarding BTC, neither KEYNOTE‐966 [41] nor TOPAZ‐1 [19] reported KM plots for patients with high or low PD‐L1 status; therefore, patient‐level data could not be acquired. KMSubtraction was utilized to infer IPD for low PD‐L1 expressing subgroups in HER2‐negative GEA, including KEYNOTE‐590 [6], CheckMate‐648 [13], ORIENT‐15 [29], CheckMate‐649 [31], KEYNOTE‐859 [30], ORIENT‐16 [15], and KEYNOTE‐062 [47]. For the remaining studies, the original published KM plots were used to derive time‐to‐event outcomes. The characteristics of these trials, including detailed KM plots, are summarized in Table S4.

Graphical reconstruction yielded individual patient‐level data with HRs and log‐rank values that closely resembled the original plots for the intent‐to‐treat populations and patients with high PD‐L1 status. There was good agreement between the original and reconstructed curves, marginal HRs, and at‐risk tables (Figure S5). Furthermore, the minimal cost bipartite matching algorithm was utilized to match PD‐L1 high expressing subgroup with the overall cohort to infer outcomes of PD‐L1 low expressing subgroup. The HR values derived from this approach was consistent with the originally reported HR values (Figure S6). In terms of effectiveness of matching, empirical cumulative distribution was adopted for each implementation of KMSubstraction. This approach led to negligible mean absolute differences between matched pairs on Bland–Altman plots. Moreover, there was nearly complete overlap between matched cohorts on KM plots. When the KMSubtraction method was implemented, negligible limits of error between the original unreported plots and the reconstructed unmatched plots were consistently observed based on simulations using 1000 Monte Carlo iterations (Figures Figures S7 and S8). Overall, these findings regarding the quality of reconstructed patient‐level data supported further survival analyses based on reconstructed KM plots.

**FIGURE 3:**
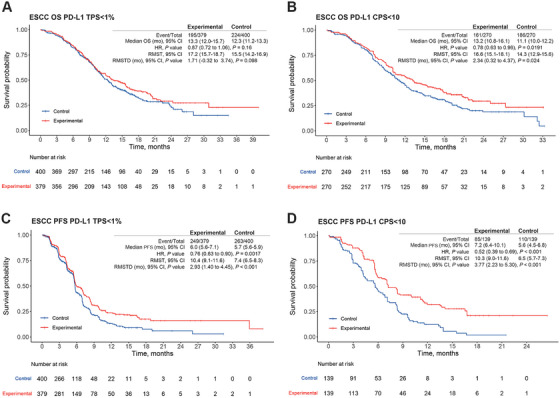
Pooled results of individual patient data (IPD) for programmed death‐ligand 1 (PD‐L1) low‐expression patients with esophageal squamous cell carcinoma (ESCC) based on the KMSubtraction method. (A) Overall survival (OS) of patient subpopulations with tumor proportion score (TPS) < 1%. (B) OS of patient subpopulations with combined positive score (CPS) < 10. (C) Progression‐free survival (PFS) of patient subpopulations with TPS < 1%. (D) PFS of patient subpopulations with CPS < 10. CI, confidence interval; HR, hazard ratio; mo, month; RMST, restricted mean survival time; RMST‐D, RMST difference.

Among low PD‐L1‐expressing ESCC patients, IPD were derived for subpopulations with TPS < 1% or CPS < 10 (Figure 3). In the subgroup with TPS < 1%, derived from the CheckMate‐648 chemo‐immunotherapy arms, Jupiter‐06, and ESCORT‐1st, the HR for OS was 0.87 (95% CI = 0.72–1.06, *p* = 0.16; Figure 3A), and the corresponding RMST‐D was 1.71 months (95% CI = −0.32 to 3.74, *p* = 0.10). However, the HR for PFS was 0.76 (95% CI = 0.63–0.90, *p* = 0.002; Figure 3C), and the corresponding RMST‐D was 2.93 months (95% CI = 1.40–4.45, *p* < 0.001). In the subgroup with CPS < 10, derived from KEYNOTE‐590 and ORIENT‐15, ICIs provided a significant OS benefit (HR = 0.78, 95% CI = 0.63–0.96, *p* = 0.02; RMST‐D = 2.34 months, 95% CI = 0.32–4.37, *p* = 0.02; Figure 3B). Similarly, a significant PFS benefit was observed (HR = 0.52, 95% CI = 0.39–0.69, *p* < 0.001; RMST‐D = 3.77, 95% CI = 2.23–5.30, *p* < 0.001; Figure 3D). Collectively, these results suggest that ESCC patients with CPS < 10 could derive survival benefits from chemo‐immunotherapy. However, ESCC patients with TPS < 1% may not experience a long survival benefit.

**FIGURE 4:**
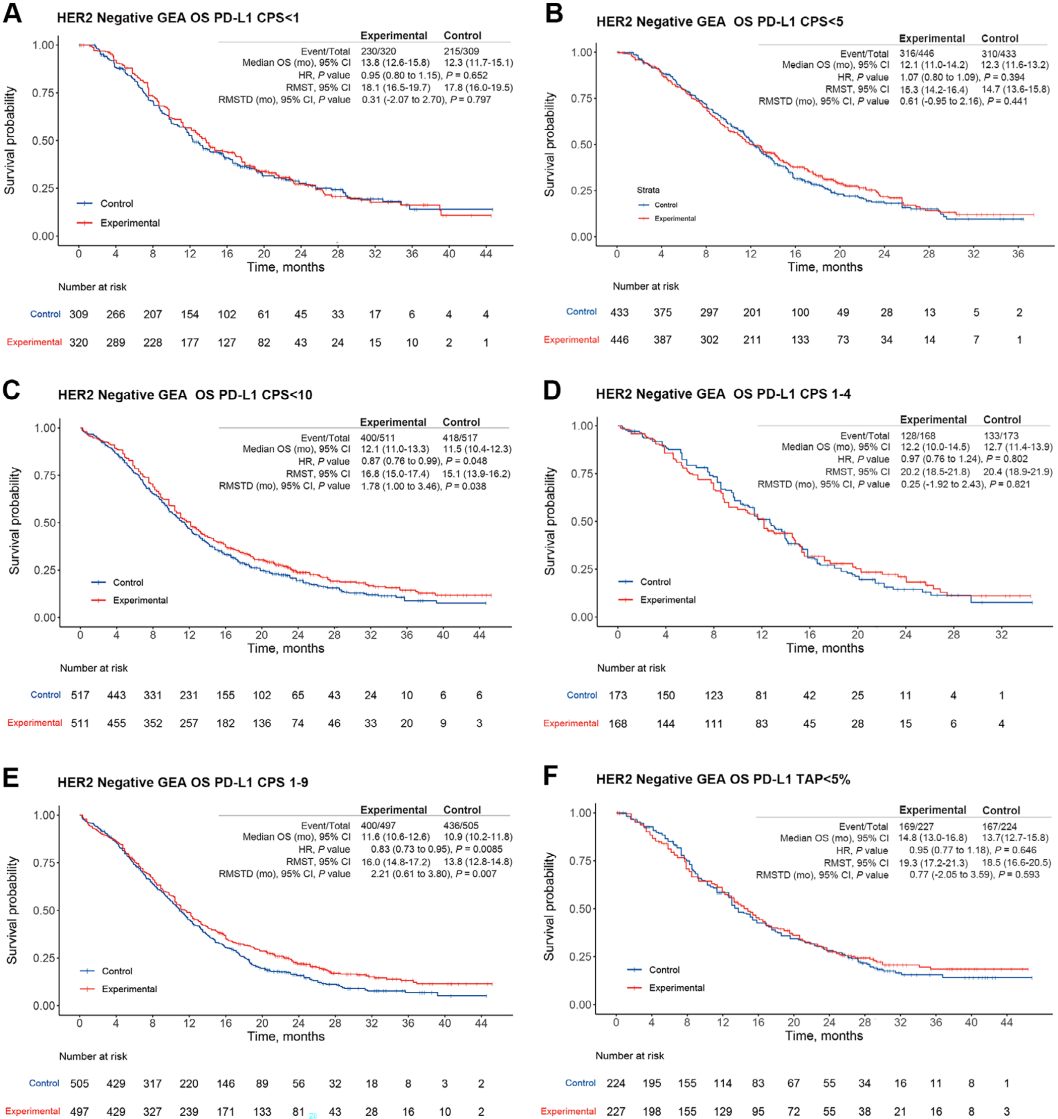
Pooled overall survival (OS) results of individual patient data (IPD) for programmed death‐ligand 1 (PD‐L1) low‐expression patients with HER2‐negative gastroesophageal adenocarcinoma (GEA) based on the KMSubtraction method. (A) OS of patient subpopulations with combined positive score (CPS) < 1. (B) OS of patient subpopulations with CPS < 5. (C) OS of patient subpopulations with CPS < 10. (D) OS of patient subpopulations with CPS 1–4. (E) OS of patient subpopulations with CPS 1–9. (F) OS of patient subpopulations with tumor area positivity (TAP) < 5%. CI, confidence interval; HR, hazard ratio; mo, month; RMST, restricted mean survival time; RMST‐D, RMST difference.

**FIGURE 5:**
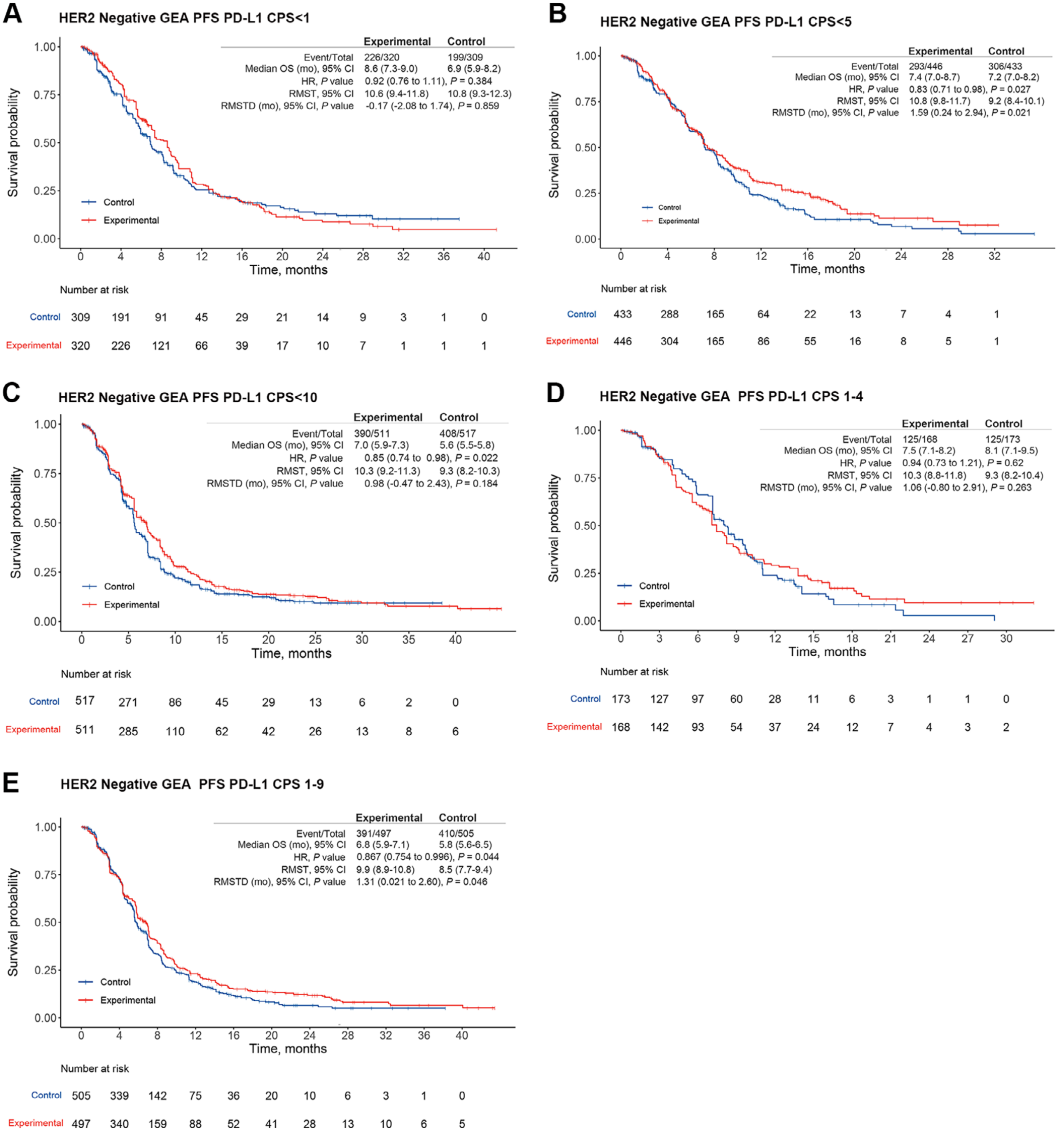
Pooled progression‐free survival (PFS) results of individual patient data (IPD) for programmed death‐ligand 1 (PD‐L1) low‐expression patients with HER2‐negative gastroesophageal adenocarcinoma (GEA) based on the KMSubtraction method. (A) PFS of patient subpopulations with combined positive score (CPS) < 1. (B) PFS of patient subpopulations with CPS < 5. (C) PFS of patient subpopulations with CPS < 10. (D) PFS of patient subpopulations with CPS 1–4. (E) PFS of patient subpopulations with CPS 1–9. CI, confidence interval; HR, hazard ratio; mo, month; RMST, restricted mean survival time; RMST‐D, RMST difference.

Among HER2‐negative GEA patients, IPD were derived for subgroups with CPS < 1, CPS < 5, CPS < 10, CPS 1–9, and CPS 1–4 (Figure 4). In patients with CPS < 1, OS did not significantly differ between ICI therapy and chemotherapy (HR = 0.95, 95% CI = 0.80–1.15, *p* = 0.65; RMST‐D = 0.31 month, 95% CI = −2.07 to 2.70, *p* = 0.80; Figure 4A) based on pooled IPD derived from CheckMate‐649 and KEYNOTE‐859. Similarly, the CPS < 5 subgroup, derived from CheckMate‐649 and ORIENT‐16, showed no significant difference in OS (HR = 1.07, 95% CI = 0.80–1.09, *p* = 0.39; RMST‐D = 0.61 month, 95% CI = −0.95 to 2.16, *p* = 0.44; Figure 4B). However, for the CPS < 10 subgroup, derived from KEYNOTE‐859, ICI therapy demonstrated a significant difference in OS (HR = 0.87, 95% CI = 0.76–0.99, *p* = 0.048; RMST‐D = 1.78, 95% CI = 1.00–3.46, *p* = 0.038; Figure 4C). In the CPS 1–4 subgroup derived from CheckMate‐649, OS did not significantly differ (HR = 0.97, 95% CI = 0.76–1.24, *p* = 0.80; RMST‐D = 0.25 month, 95% CI = −1.92 to 2.43, *p* = 0.82; Figure 4D). In the CPS 1–9 subgroup derived from KEYNOTE‐859 and KEYNOTE‐062, a significant difference in OS was observed (HR = 0.83, 95% CI = 0.73–0.95, *p* < 0.01; RMST‐D = 2.21 months, 95% CI = 0.61–3.80, *p* = 0.007; Figure 4E). Similar trends were observed regarding benefits in PFS for these patient subgroups, except for group of CPS < 5, in which the experimental arm showed only a marginal benefit (Figure 5A–E). For CPS < 10 subgroup, ICI could effectively reduce the risk of progression while not able to significantly prolong PFS (HR = 0.85, 95% CI = 0.74–0.98, *p* = 0.02; RMST‐D = 0.98, 95% CI = −0.47 to 2.43, *p* = 0.18; Figure 5C). For CPS 1–9 subgroup, ICI could both reduce the risk of progression and prolong absolute gain in PFS interval (HR = 0.867, 95% CI = 0.754–0.996, *p* < 0.05; RMST‐D = 1.31 months, 95% CI = 0.02–2.60, *p* < 0.05; Figure 5E). Overall, the above analysis indicates that a CPS of 5 may be a meaningful cutoff value for identifying HER2‐negative GEA patients who could benefit from ICI therapy.

## 3 Discussion

ICI‐based therapy has been approved worldwide as standard first‐line therapy for various solid tumors. The PD‐L1 expression level, quantified by immunohistochemistry, is the most widely accepted biomarker for ICI treatment in many clinical guidelines; this biomarker is also acknowledged by most medical insurance reimbursement policies [7]. The clinical utility of recommended PD‐L1 cutoff values considerably varies among cancers and treatment schedules [4, 5]. Although the FDA has approved the use of ICIs for several cancers regardless of PD‐L1 status in certain treatment settings, the actual efficacy of ICIs among patients with low PD‐L1 expression remains unclear. KM curves for such patients in many phase III trials are often unreported, hindering calculation of the actual efficacy [7]. Furthermore, the divergent guidelines of the FDA and EMA for patients with low PD‐L1 expression underscore the importance of exploring this clinical issue [7]. Therefore, we utilized study‐ and patient‐level data to conduct a comprehensive analysis regarding the actual clinical benefit of PD‐1/PD‐L1 inhibitors as first‐line treatment for low PD‐L1‐expressing advanced solid tumors, in accordance with FDA guidelines. This evaluation of the optimal PD‐L1 cutoff value for certain cancers is important for clinicians and malignant tumor patients, as well as healthcare policymakers.

In this systematic review and meta‐analysis of 40 studies involving 27,060 patients and 10 different cancers, we observed a trend toward better efficacy among patients with high PD‐L1 expression in most cancers (e.g., NSCLC, ESCC, HER2‐negative GEA, HCC, MPM, BTC, and UC). Conversely, among patients with low PD‐L1 expression, individuals with some cancers may not benefit from immunotherapy. According to pooled results from ≥ 2 clinical trials, we found no statistically significant survival benefit in low PD‐L1‐expressing ESCC and BTC, suggesting that patients with low PD‐L1 expression who have these cancers are less likely to derive benefits from immunotherapy compared with patients displaying high PD‐L1 expression. In the HCC and melanoma subgroups, patients did not experience a survival benefit regardless of PD‐L1 status, suggesting that PD‐L1 is not a suitable biomarker for determining ICI suitability among patients with these cancers. Additionally, we observed that patients with low PD‐L1‐expressing HER2‐negative GEA derived a marginal benefit, which hindered conclusions regarding survival benefit solely based on HR findings. Therefore, we conducted a comprehensive analysis of cancers that exhibited marginal or no survival benefit in patients low PD‐L1 scores. The IPDfromKM and KMSubtraction methods were utilized to infer time‐to‐event outcomes among patients with low PD‐L1 scores; secondary analyses were conducted to investigate ICI efficacy among these patients using different cutoff values and scoring methods.

As previously mentioned, esophageal cancer exemplifies the heterogeneity in regulatory policies between the FDA and EMA [4, 5]. According to the EMA, pembrolizumab is only approved for ESCC patients with CPS ≥ 10, whereas nivolumab is only approved for ESCC patients with TC ≥ 1% [5]. In contrast, both pembrolizumab and nivolumab have received FDA approval for ESCC patients regardless of PD‐L1 status [4]. Previous meta‐analyses yielded conflicting results regarding the efficacy of ICIs for patients with low PD‐L1‐expressing ESCC. Yap et al. [8] conducted a pooled analysis of IPD, which suggested an OS benefit for the subpopulation with CPS < 10 but not the subpopulation with TPS < 1%. However, a post hoc analysis of Jupiter‐06 and a study‐level meta‐analysis by Wu et al. [48] indicated the superiority of ICI therapy among patients with CPS < 10 or TPS < 1%. Subsequent commentary regarding the analysis by Yu et al. [49] criticized its methodology and suggested that PD‐1 antibody did not provide an OS benefit in the TPS < 1% subgroup.

In the present study, which included six studies for pooled analysis, both HR and RMST‐D assessments suggested no OS benefit among ESCC patients with TPS < 1%. We also observed a significant OS benefit among patients with CPS < 10, consistent with previous studies. These findings imply that TPS is a more appropriate method for assessing PD‐L1 staining results during ESCC patient selection for ICI therapy. Furthermore, significant PFS benefits were observed among ESCC patients with CPS < 10 and TPS < 1%. This divergence between OS and PFS outcomes has been documented in previous studies; some researchers have suggested that PFS is not a suitable metric for evaluating immunotherapy efficacy [50]. A recent meta‐analysis concerning different endpoints for immunotherapy outcomes also suggested a weak correlation (*R* ^2^ = 0.4) between PFS and clinical outcomes [51]. Considering the limited evidence in this patient subgroup, our results could facilitate improved stratification according to PD‐L1 scores. Overall, based on our findings, we propose using TPS ≥ 1% as an optimal cutoff value when performing patient selection for ICI therapy because ESCC patients with TPS < 1% may not experience a survival benefit.

Regarding GEA patients, the FDA recently restricted the use of pembrolizumab in the treatment of HER2‐positive GEA to patients with positive PD‐L1 expression only based on updated results from KEYNOTE‐811 [52]. Additionally, the FDA approved pembrolizumab in combination with chemotherapy for HER2‐negative GEA regardless of PD‐L1 status based on results from KEYNOTE‐859 [4, 30]. Therefore, we focused on the subpopulation of patients with HER2‐negative GEA and low PD‐L1 expression. Previous meta‐analyses have investigated the efficacy of ICI therapy for GEA from various perspectives. For example, a recent meta‐analysis by Xie et al. [53] suggested that ICIs combined with chemotherapy could provide wide‐ranging survival benefits. However, this conclusion was based on pooled analysis of all relevant clinical trials regardless of treatment line, FDA regulatory policy, and HER2 status. An analysis by Zhao et al. [7] also indicated a lack of benefits from chemo‐immunotherapy in the CheckMate‐649 CPS 1–4 subgroup, as well as the KEYNOTE‐062 CPS 1–9 subgroup; this analysis utilized the KMSubtraction method but only inferred the efficacy of chemo‐immunotherapy from two clinical trials with multiple HER2 statuses. We note that both Xie et al. [53] and Zhao et al. [7] did not clearly determine whether current FDA guidelines regarding PD‐L1 status are suitable for HER2‐negative GEAs with low PD‐L1 expression (CPS < 1).

In our analysis of HER2‐negative GEA, study‐level data suggested that patients with low or negative PD‐L1 expression could derive marginal benefit from ICIs; this finding appears to contradict the results of patient‐level data. The discrepancy likely arose because different PD‐L1 score systems and cutoff values were incorporated in the study‐level analysis, whereas the patient‐level results were calculated based on CPS and a consistent PD‐L1 cutoff value. During the patient‐level analysis, we utilized the KMSubtraction method to reconstruct the time‐to‐event outcomes of five relevant trials, then analyzed ICI efficacy in various PD‐L1 subgroups (CPS < 1, CPS 1–4, CPS < 5, CPS 1–9, and CPS < 10). We observed OS benefits from ICI therapy only in the CPS < 10 and CPS 1–9 subgroups. Similar trends were observed regarding the PFS benefit, although RMST‐D assessment suggested no significant benefit in the CPS < 10 subgroup. Despite the contradictory RMST‐D and HR results regarding efficacy, the presence of an intersection between the survival curves of the two arms on KM plots suggested that the RMST‐D result was more statistically robust. Furthermore, the CPS 1–9 subgroup could derive a PFS benefit but the CPS < 1 subgroup could not. Therefore, the lack of PFS benefit in the CPS < 10 subgroup might be primarily driven by the lack of benefit among PD‐L1‐negative patients.

Currently, the FDA and EMA have different regulatory policies regarding patients with HER2‐negative GEA. The FDA has approved both pembrolizumab and nivolumab for the treatment of HER2‐negative GEA, regardless of PD‐L1 status; conversely, the EMA, opting for a more conservative approval approach, has approved the use of these drugs only for patients with CPS ≥ 10. Our results may help to rectify this discrepancy; they suggest that CPS ≥ 5 is an ideal biomarker for identifying HER2‐negative GEA patients who could benefit from ICI therapy. For RATIONALE‐305, which is the only study that adopts TAP for PD‐L1 staining among patients with HER2‐negative GEA, patients with TAP ≥ 5 could potentially derive better survival benefits compared to patients with TAP < 5 based on the published results at 2023 ESMO conference, highlighting the utility of TAP scores. However, we failed to reconstruct the survival plots as the KM plots have not been published and it is difficult to make definitive conclusions based on a single study. Accordingly, the optimal PD‐L1 cutoff value for identifying patients likely to benefit from ICIs should be reconsidered by regulatory agencies and clinicians.

The lack of benefit observed in patients with low PD‐L1 expression from ICIs can be attributed to several factors. First, monoclonal antibodies such as anti‐PD‐1 and anti‐PD‐L1 rely on sufficient PD‐L1 expression on cell surface to effectively block the PD‐1/PD‐L1 interaction. Without adequate PD‐L1 expression, the inhibition of PD‐1/PD‐L1 pathway is less efficient, resulting in diminished anti‐tumor immune responses [54]. Second, the tumor immune microenvironment (TIME) plays a critical role in shaping the efficacy of immune therapies [55]. Even when PD‐1/PD‐L1 interactions are blocked, other immunosuppressive factors such as metabolic by‐products or myeloid‐derived suppressor cells (MDSCs) may hinder anti‐tumor immunity [56]. Finally, while anti‐PD‐1 and anti‐PD‐L1 therapies activate systemic immune responses, they also require efficient immune cell infiltration into the tumor [57]. Tumor vasculature abnormalities, such as a dense extracellular matrix or poorly structured blood vessels, can impede immune cell access to the tumor site, preventing the activation of localized anti‐tumor immune responses [58]. These factors collectively contribute to the reduced efficacy of ICIs in patients with low PD‐L1 expression.

Compared with previous works, our study has several distinct advantages. First, our inclusion criteria were closely aligned with current FDA regulatory policies, particularly regarding mutation status and molecular subtyping, and the included studies were either cited by FDA as evidence for drug approval or conducted in parallel to these studies cited by FDA regarding dosing regimen, cancer types, and drug target. This approach ensures that our findings are highly relevant to clinical practice. Second, the KMSubtraction method was effectively utilized to reveal unreported subgroups with negative PD‐L1 expression, as well as subgroups with various degrees of low PD‐L1 expression. Third, RMST‐D was incorporated into each prognostic analysis as a robust, nonparametric complement to HR; this metric can be intuitively interpreted as an absolute gain (or loss) of survival time [10].

Nevertheless, our findings should be interpreted cautiously due to some inevitable limitations. First, although we categorized the included studies according to their PD‐L1 counting methods, variation persisted in terms of the PD‐L1 assays used for testing [59]. Second, the inference of IPD using the KMSubtraction method was considerably limited by data availability. For example, we were unable to reconstruct patient‐level data for BTC due to a lack of KM plots; however, we found that patients with low PD‐L1‐expressing BTC also did not derive survival benefits from ICI therapy. Additional post hoc studies are needed to address this limitation. Finally, due to the absence of comprehensive patient‐level data, our analyses were constrained by predefined dichotomies within the trials themselves. Thus, we could not explore the precise PD‐L1 cutoff values at which a patient may begin to benefit from ICIs, nor could we adjust for other related patient‐level covariates that may influence ICI efficacy.

In conclusion, our study provides a comprehensive assessment of the clinical benefit from first‐line ICI therapy for solid tumors with low PD‐L1 expression, in the context of current guidelines. We found no evidence of a survival benefit from ICIs in the first‐line setting for patients with low or negative PD‐L1‐expressing BTC, HCC, and melanoma. Importantly, we demonstrated that the addition of ICIs to conventional chemotherapy does not provide additional benefit for ESCCs with TPS < 1% and HER2‐negative GEAs with CPS < 5. These findings indicate that clinicians should utilize caution in the treatment of solid tumors with low PD‐L1 expression; they may encourage more nuanced application of the PD‐L1 score and prompt regulatory agencies to revise their guidelines.

## 4 Materials and Methods

### 4.1 Search Strategy and Selection Criteria

For this meta‐analysis, we initially identified all types of ICIs and their FDA‐approved regimens. The FDA‐approved regimens for first‐line therapy without PD‐L1‐based restrictions are presented in Table S1. Subsequently, we conducted a systematic search in Embase and PubMed for phase III randomized controlled trials (RCTs) from database inception until November 30, 2023. An updated search for literatures published between December 1, 2023 to August 1, 2025 on PubMed was subsequently conducted to ensure timeliness of this analysis. The search strategy is detailed in Table S2. To ensure comprehensive review, meeting abstracts from the American Association for Cancer Research, American Society of Clinical Oncology, and European Society of Medical Oncology, as well as reference lists from relevant reviews and meta‐analyses, were also examined. In cases of multiple publications from the same trial, the most recent and comprehensive publication was included.

The inclusion and exclusion criteria were prespecified. An eligible RCT met the following inclusion criteria: (1) it was cited by FDA guidelines as a reference for regulatory approval (i.e., FDA cited studies in Table S3); or (2) it was conducted in parallel to the FDA cited study and had identical trial characteristics, including drug targets (PD‐1 or PD‐L1), tumor types, mutational statuses, molecular subtypes, treatment lines, intervention regimens, control groups, and biomarker statuses (i.e., FDA parallel studies in Table S3). All included trials were required to report both PD‐L1 biomarker status and OS outcomes. The exclusion criteria were as follows: (1) Trials evaluating ICI efficacy in cancers not approved by the FDA for use without PD‐L1‐based restrictions (e.g., IMpower 151 [23] [NSCLC with driver gene mutations], KEYNOTE‐811 [52] [HER2‐positive GC without CPS ≥1 restriction], and IMpower131 [60] [squamous NSCLC]). (2) Trials that only included specific patient populations, such as older adults, teenagers, or PD‐L1 positive patients. (3) Trials reporting results from a subgroup analysis that was a potential source of unclear randomization and corresponding bias. (4) Trials comparing efficacy between ICIs and other immunotherapies (e.g., chimeric antigen receptor [CAR]‐T and vaccines). (5) Trials evaluating ICI efficacy as adjuvant or neoadjuvant therapy.

Potential bias was evaluated using the Cochrane risk of bias tool (RoB2) [61]. Discrepancies were resolved through discussion with a corresponding author (S.N. or H.J.), with the goal of reaching a consensus.

This study protocol was designed and conducted in accordance with the Preferred Reporting Items for Systematic Reviews and Meta‐analyses (PRISMA) reporting guideline [62]. The protocol was prospectively registered in the Prospective Register of Systematic Reviews (PROSPERO; CRD42023441048).

### 4.2 Data Analysis

Data extracted from eligible studies were the authors, publication years, trial names, types of cancers, PD‐1 and PD‐L1 inhibitor names, combination agent names, PD‐L1 staining methods, and numbers of patients with low PD‐L1 expression in each arm, as well as the HRs (95% CIs^24^) of OS among patients in different PD‐L1 subgroups. The primary endpoint was the ICI efficacy across different cancers, as measured by the HR of OS. Inter‐study heterogeneity was assessed using Cochrane's *Q* statistic and the *I* ^2^ statistic. The pooled HR of OS was calculated using random‐effects model for subgroups with high level of heterogeneity (*I* ^2^ ≥ 50%), otherwise common‐effects model was adopted. All reported *p*‐values were two‐sided, and *p*‐values < 0.05 were considered statistically significant. Sensitivity analysis was performed by leave‐one‐out method to assess the stability of the results.

We conducted one‐stage pooled analysis using derived IPD for cancers of interest. Time‐to‐event outcomes were extracted from relevant KM plots via methods previously described by Guyot et al. [63] and Neuen et al. [64]. Briefly, we used the web‐based software WebPlotDigitizer (https://apps.automeris.io/wpd/index.zh_ CN.html) to extract data from curve graphs, primarily through reverse engineering and visualization of data images to obtain key numerical data. Then, we retrieved time‐to‐event outcomes from KM curves using the R package IPDfromKM, developed by Liu [65]. The quality of reconstruction was assessed based on reconstructed HRs, at‐risk tables, median survival times, and the shapes of KM curves.

In trials lacking KM plots for patients with low PD‐L1 expression, KMSubtraction was utilized to infer time‐to‐event data from the reconstructed datasets of the overall cohort and the high PD‐L1 expression subgroup. The KMSubtraction algorithm infers unreported subgroup survival data using information from known subgroups. The algorithm primarily consists of three functions: KMSubtractionMatch (for patient matching), KMSubtractionEvaluateMatch (for assessing match quality), and KMSubtractionError (for calculating standard errors).

Minimal cost bipartite matching was mainly performed with the Hungarian algorithm; unmatched patients constituted the unreported group. Match quality was evaluated using the Bland–Altman, empirical cumulative distribution function, and Kolmogorov–Smirnov statistical tests, as well as KM plots. Deviations from true values were represented by RMST‐D and the absolute value of ln(HR). Monte Carlo simulations with 1000 iterations were conducted to evaluate error boundaries between original and reconstructed data.

Survival analysis was conducted when the match quality was acceptable and HR error was minimal. To investigate treatment benefit among patients with low PD‐L1 expression, KMSubtraction‐derived IPD were collected for pooled analysis. PD‐L1 expression was categorized using multiple scoring systems (TPS, CPS, and TAP) and various cutoff values. Time‐to‐event outcomes were analyzed using KM curves and stratified log‐rank tests, with stratification according to PD‐L1 score. Cox proportional hazards regression was performed to estimate HRs and 95% CIs, adjusted for stratification factors. RMST statistics were calculated using the R package survRM2 (version 1·0‐4); *p*‐values < 0.05 indicated statistical significance.

## Author Contributions

Conceptualization and supervision and funding acquisition: Chaoqi Zhang and Jie He. Formal analysis: Peng Wu, Xuanyu Gu, and Dongyu Li. Visualization: Xiaohui Zi, Dexin Shang, Jingjing Liu, and Xuanyu Gu. Data collection and curation, interpretation of data, writing – original draft, and writing – review and editing: Chaoqi Zhang and Jie He. Jie He and Chaoqi Zhang had full access to all of the data in the study and take responsibility for the integrity of the data and the accuracy of the data analysis.

## Funding

This work is supported in part by the Beijing Municipal Science & Technology Commission (No. Z22110000742201), the CAMS Innovation Fund for Medical Sciences (grant number 2024‐I2M‐ZD‐004), the National Natural Science Foundation of China (82188102 and 82203025), the China Postdoctoral Science Foundation (2024M750249), and the Special Research Fund for Central Universities, Peking Union Medical College (3332024052).

## Ethics Statement

The authors have nothing to report.

## Conflicts of Interest

The authors declare no conflicts of interest.

## Supporting information




**Supporting Information file 1**: mco270624‐sup‐0001‐SuppMat.docx

## Data Availability

All data relevant to this study are included in the article or in the supporting information file 1
