# Supplementary material for: Clinical Benefit of PD‐1/PD‐L1 Inhibitors as Standard First‐Line Treatment in Low PD‐L1‐Expressing Advanced Solid Tumors: A Systematic Review and Meta‐Analysis
Source: MedComm (2020). 2026 Feb 9;7(2):e70624. doi: 10.1002/mco2.70624 (PMC12887441; doi:10.1002/mco2.70624)
Supplement: Supplementary file 1 — Supporting Information file 1: mco270624‐sup‐0001‐SuppMat.docx [file MCO2-7-e70624-s001.docx]

**C****linical benefit of PD-1/PD-L1 inhibitors as standard first-line treatment in low PD-L1-expressing advanced solid tumors: a systematic review and meta-analysis**

Peng Wu^1#^, Xuanyu Gu^1#^, Dongyu Li^1#^, Xiaohui Zi^1#^, Dexin Shang^1^, Jingjing Liu^1^, Ruijie Ma^1^, Jilin Peng^2^, Guochao Zhang^1^, Yun Che^1^, Qingpeng Zeng^1^, Bohui Zhao^1^, Nan Sun^1^, Chaoqi Zhang^1^*, Jie He^1^*

1. Department of Thoracic Surgery, National Clinical Research Center for Cancer/Cancer Hospital, National Cancer Center, Chinese Academy of Medical Sciences, Peking Union Medical College, Beijing, 100021, China.
2. Department of Otolaryngology-Head and Neck Surgery, The First Affiliated Hospital of Zhengzhou University, Zhengzhou, 450052, China.

Correspondence to:

Prof. Jie He M.D. & Ph.D., Department of Thoracic Surgery, National Cancer Center/National Clinical Research Center for Cancer/Cancer Hospital, Chinese Academy of Medical Sciences and Peking Union Medical College, Beijing 100021, China. Email: prof.jiehe@gmail.com;

Prof. Chaoqi Zhang M.D. & Ph.D., Department of Thoracic Surgery, National Cancer Center/National Clinical Research Center for Cancer/Cancer Hospital, Chinese Academy of Medical Sciences and Peking Union Medical College, Beijing 100021, China. Email: chaoqizhang@cicams.ac.cn.

# Co-first authors, contributed equally.

* Co-corresponding authors, contributed equally.

**Supplementary Materials**

**Content**

[Table S1. FDA and EMA regulatory policies of anti-PD-1 and anti-PD-L1 as first line for solid tumors. 2](#_Toc213176887)

[Table S2. Search strategy 5](#_Toc213176888)

[Table S3. Characteristics of included clinical trials in the meta-analysis. 8](#_Toc213176889)

[Table S4. Search information and where KMSubtraction was Implemented. 11](#_Toc213176890)

[Figure S1. Risk of bias and quality assessment of RCTs using RoB2 14](#_Toc213176891)

[Figure S2. Random forest for overall survival (OS) outcome in high programmed death ligand (PD-L1) subgroups by tumor type. 15](#_Toc213176892)

[Figure S3. Sensitivity test for low programmed death ligand (PD-L1) subgroups 17](#_Toc213176893)

[Figure S4. Sensitivity test for high programmed death ligand (PD-L1) subgroups 18](#_Toc213176894)

[Figure S5. Comparison of original and reconstructed figures for overall and high/ low programmed death ligand (PD-L1) subgroups 19](#_Toc213176895)

[Figure S6. Reported hazard ratio (HR) for overall survival (OS) and progression free survival (PFS) in programmed death ligand (PD-L1) low subgroup using KMSubtraction workflow. 39](#_Toc213176896)

[Figure S7. Evaluation of KMSubtraction Bipartite Matching 52](#_Toc213176897)

[Figure S8. Convergence plots and histograms of simulations 99](#_Toc213176898)

# Table S1. FDA and EMA regulatory policies of anti-PD-1 and anti-PD-L1 as first line for solid tumors.

**Pembrolizumab：**

**Nivolumab:**

**Cemiplimab:**

**Dostarlimab:**

**Retifanlimab:**

**Durvalumab:**

**Atezolizumab:**

**Avelumab:**

**Abbreviation:**

FDA=Food and Drug Administration. EMA=European Medicines Agency. NSCLC=non-small cell lung cancer. HNSCC=head and neck squamous cell carcinoma. MPM=malignant pleural mesothelioma. GC=Gastric Cancer. ESCC=esophageal squamous cell carcinoma. RCC=renal cell carcinoma. cSCC=cutaneous squamous cell carcinoma. TNBC=triple negative breast cancer. EC=endometrial carcinoma. SCLC=small cell lung cancer. HCC=hepatocellular carcinoma. ASPS=alveolar soft part sarcoma. BTC=biliary tract cancer. UC=urothelial carcinoma. MCC=merkel cell carcinoma. Chemo=chemotherapy. TKIs=tyrosine kinase inhibitors. PD-L1=programmed death-ligand

# Table S2. Search strategy

| **Date** | Inception - 2025.05.30 |
| --- | --- |
| **Database** | **Keywords** |
| **1. Pubmed** |  |
| #1 | (((((((((((((((((((((((((((((((lung cancer[Title/Abstract]) OR (non small cell lung cancer[Title/Abstract])) OR (lung adenocarcinoma[Title/Abstract])) OR (lung squamous cell carcinoma[Title/Abstract])) OR (non-squamous non-small cell lung carcinoma[Title/Abstract])) OR (squamous non-small cell lung carcinoma[Title/Abstract])) OR (melanoma[Title/Abstract])) OR (Hodgkin lymphoma[Title/Abstract])) OR (urothelial carcinoma[Title/Abstract])) OR (Head and Neck squamous cell carcinoma[Title/Abstract])) OR (renal cell carcinoma[Title/Abstract])) OR (colorectal cancer[Title/Abstract])) OR (Oesophageal carcinoma[Title/Abstract])) OR (esophageal cancer[Title/Abstract])) OR (esophageal squamous cell carcinoma[Title/Abstract])) OR (breast cancer[Title/Abstract])) OR (endometrial carcinoma[Title/Abstract])) OR (cervical cancer[Title/Abstract])) OR (gastric cancer[Title/Abstract])) OR (gastro-oesophageal junction adenocarcinoma[Title/Abstract])) OR (hepatocellular carcinoma[Title/Abstract])) OR (biliary tract[Title/Abstract])) OR (malignant pleura mesothelioma[Title/Abstract])) OR (small cell lung cancer[Title/Abstract])) OR (merkel cell carcinoma[Title/Abstract])) OR (Cutaneous Squamous Cell Carcinoma[Title/Abstract])) OR (basal cell carcinoma[Title/Abstract]) ) OR (gastroesophageal junction cancer[Title/Abstract]) ) OR (gastro-oesophageal junction cancer[Title/Abstract])) OR (oesophageal squamous cell carcinoma[Title/Abstract])) OR (squamous NSCLC[Title/Abstract])) OR (esophageal squamous-cell carcinoma[Title/Abstract]) |
| #2 | (((((((((((((((((((((((Pembrolizumab[Title/Abstract]) OR (Nivolumab[Title/Abstract])) OR (Cemiplimab[Title/Abstract])) OR (Atezolizumab[Title/Abstract])) OR (Dostarlimab[Title/Abstract])) OR (durvalumab[Title/Abstract])) OR (avelumab[Title/Abstract])) OR (retifanlimab[Title/Abstract])) OR (Toripalimab[Title/Abstract])) OR (sintilimab[Title/Abstract])) OR (camrelizumab[Title/Abstract])) OR (Tislelizumab[Title/Abstract])) OR (Serplulimab[Title/Abstract])) OR (Zimberelimab[Title/Abstract])) OR (Envafolimab[Title/Abstract])) OR (Sugemalimab[Title/Abstract])) OR (Adebrelimab[Title/Abstract])) OR (Pucotenlimab[Title/Abstract])) OR (Socazolimab[Title/Abstract])) OR (Immune checkpoint inhibitor[Title/Abstract])) OR (Immune checkpoint blockage[Title/Abstract])) OR (Programmed Cell Death 1 Receptor[Title/Abstract])) OR (PD-1[Title/Abstract])) OR (PD-L1[Title/Abstract]) |
| #3 | #1 AND #2 |
| #4 | (clinicaltrial[Filter] OR randomizedcontrolledtrial[Filter]) |
| #5 | #3 AND #4 |
| **2. Embase** |  |
| #1 | ('melanoma':ab,ti OR 'non small cell lung cancer':ab,ti OR 'small cell lung cancer':ab,ti OR 'squamous cell lung carcinoma':ab,ti OR 'lung adenocarcinoma':ab,ti OR 'hodgkin disease':ab,ti OR 'urothelial tumor':ab,ti OR 'head and neck tumor':ab,ti OR 'renal cell carcinoma':ab,ti OR 'colorectal cancer':ab,ti OR 'esophagus cancer':ab,ti OR 'esophagus carcinoma':ab,ti OR 'stomach cancer':ab,ti OR 'gastroesophageal junction adenocarcinoma':ti,ab OR 'gastroesophageal junction cancer':ab,ti OR 'gastroesophageal cancer':ab,ti OR 'liver cancer':ab,ti OR 'breast carcinoma':ab,ti OR 'endometrium carcinoma':ab,ti OR 'uterine cervix cancer':ab,ti OR 'biliary tract cancer':ab OR 'pleura mesothelioma':ab,ti OR 'merkel cell carcinoma':ab,ti OR 'squamous cell skin carcinoma':ab,ti OR 'basal cell carcinoma':ab,ti) AND [article]/lim AND [english]/lim AND [embase]/lim AND [<1966-2023]/py |
| #2 | ('pembrolizumab':ab,ti OR 'nivolumab':ab,ti OR 'cemiplimab':ab,ti OR 'retifanlimab':ab,ti OR 'atezolizumab':ab,ti OR 'durvalumab':ab,ti OR 'dostarlimab':ab,ti OR 'avelumab':ab,ti OR 'toripalimab':ab,ti OR 'sintilimab':ab,ti OR 'camrelizumab':ti,ab OR 'tislelizumab':ab,ti OR 'serplulimab':ab,ti OR 'zimberelimab':ab,ti OR 'envafolimab':ab,ti OR 'sugemalimab':ab,ti OR 'adebrelimab':ab,ti OR 'pucotenlimab':ab,ti OR 'socazolimab':ab,ti OR 'ipilimumab':ab,ti OR 'tremelimumab':ab,ti) AND [article]/lim AND [english]/lim AND [embase]/lim AND [<1966-2023]/py |
| #3 | #1 AND #2 |
| #4 | #3 AND ('clinical trial'/de OR 'phase 3 clinical trial'/de OR 'randomized controlled trial'/de OR 'controlled clinical trial'/de) |

# Table S3. Characteristics of included clinical trials in the meta-analysis.

| **Clinical trial** | **NCT ID** | **Year** | **Target** | **Type** | **Intervention** | **Control** | **Group** |
| --- | --- | --- | --- | --- | --- | --- | --- |
| TOPAZ1 | NCT03875235 | 2022 | PD-L1 | BTC | Durvalumab + Chemo | Placebo + Chemo | FDA cited |
| KEYNOTE-966 | NCT04003636 | 2023 | PD-1 | BTC | Pembrolizumab + Chemo | Placebo + Chemo | FDA cited |
| CHECKMATE-648 | NCT03143153 | 2022 | Arm1: PD-1+CTLA-4  Arm2: PD-1 | ESCC | Arm1: Nivolumab + Ipilimumab Arm2: Nivolumab + Chemo | Chemo | FDA cited |
| ESCORT-1^st^ | NCT03691090 | 2021 | PD-1 | ESCC | Camrelizumab + Chemo | Placebo + Chemo | FDA parallel |
| KEYNOTE-590 | NCT03189719 | 2021 | PD-1 | ESCC | Pembrolizumab + Chemo | Placebo + Chemo | FDA cited |
| ORlENT-15 | NCT03748134 | 2022 | PD-1 | ESCC | Sintilimab + Chemo | Placebo + Chemo | FDA parallel |
| RATIONALE-306 | NCT03783442 | 2023 | PD-1 | ESCC | Tislelizumab + Chemo | Placebo + Chemo | FDA parallel |
| JUPITER-06 | NCT03829969 | 2022 | PD-1 | ESCC | Toripalimab + Chemo | Placebo + Chemo | FDA parallel |
| ATTRACTION-4 | NCT02746796 | 2022 | PD-1 | HER2^-^ GEA | Nivolumab + Chemo | Placebo + Chemo | FDA cited |
| KEYNOTE-859 | NCT03675737 | 2023 | PD-1 | HER2^-^ GEA | Pembrolizumab + Chemo | Placebo + Chemo | FDA cited |
| ORIENT-16 | NCT03745170 | 2023 | PD-1 | HER2^-^ GEA | Sintilimab + Chemo | Placebo +Chemo | FDA parallel |
| RATIONALE-305 | NCT03777657 | 2023 | PD-1 | HER2^-^ GEA | Tislelizumab + Chemo | Placebo + Chemo | FDA parallel |
| IMbrave150 | NCT03434379 | 2022 | PD-L1 | HCC | Atezolizumab + Bevacizumab | Sorafenib | FDA cited |
| HIMALAYA | NCT03298451 | 2023 | PD-L1 | HCC | Durvalumab + Tremelimumab | Sorafenib | FDA cited |
| IMspire150 | NCT02908672 | 2023 | PD-L1+BRAF V600E | Melanoma | Atezolizumab + Vemurafenib + Cobimetinib | Placebo + Vemurafenib + Cobimetinib | FDA cited |
| CHECKMATE-066 | NCT01721772 | 2015 | PD-1 | Melanoma | Nivolumab | Chemo | FDA cited |
| CHECKMATE-743 | NCT02899299 | 2022 | PD-1 + CTLA-4 | MPM | Nivolumab + Ipilimumab | Chemo | FDA cited |
| **Clinical trial** | **NCT ID** | **Year** | **Target** | **Type** | **Intervention** | **Control** | **Group** |
| IMpower150 | NCT02366143 | 2021 | Arm1: PD-L1 + VEGF  Arm2: PD-L1 | Non-Sq NSCLC | Arm1: Atezolizumab + Bevacizumab + Chemo;  Arm2: Atezolizumab + Chemo | Chemo | FDA cited |
| IMpower130 | NCT02367781 | 2019 | PD-L1 | Non-Sq NSCLC | Atezolizumab + Chemo | Placebo + Chemo | FDA cited |
| IMpower132 | NCT02657434 | 2021 | PD-L1 | Non-Sq NSCLC | Atezolizumab + Chemo | Chemo | FDA parallel |
| KEYNOTE-189 | NCT02578680 | 2023 | PD-1 | Non-Sq NSCLC | Pembrolizumab + Chemo | Placebo + Chemo | FDA cited |
| ORIENT-11 | NCT03607539 | 2021 | PD-1 | Non-Sq NSCLC | Sintilimab + Chemo | Placebo + Chemo | FDA parallel |
| POSEIDON | NCT03164616 | 2023 | Arm1: PD-L1 + CTLA-4  Arm2: PD-L1 | NSCLC | Arm1: Durvalumab + Tremelimumab + chemo;  Arm2: Durvalumab + Chemo | Chemo | FDA cited |
| EMPOWER-LUNG3 | NCT03409614 | 2022 | PD-1 | NSCLC | Cemiplimab + Chemo | Placebo + Chemo | FDA cited |
| PACIFIC | NCT02125461 | 2022 | PD-L1 | NSCLC | Durvalumab | Chemoradiotherapy + Placebo | FDA cited |
| CheckMate-9LA | NCT03215706 | 2021 | PD-1 + CTLA-4 | NSCLC | Nivolumab + Ipilimumab + Chemo | Chemo | FDA cited |
| Gemstone-302 | NCT03789604 | 2023 | PD-L1 | NSCLC | Sugemalimab + Chemo | Placebo + Chemo | FDA parallel |
| CHOICE-01 | NCT03856411 | 2023 | PD-1 | NSCLC | Toripalimab + Chemo | Placebo + Chemo | FDA parallel |
| **Clinical trial** | **NCT ID** | **Year** | **Target** | **Type** | **Intervention** | **Control** | **Group** |
| AK105-302 | NCT03866993 | 2024 | PD-1 | Sq NSCLC | Penpulimab + Chemo | Placebo+Chemo | FDA parallel |
| CameL-sq | NCT03668496 | 2022 | PD-1 | Sq NSCLC | Camrelizumab + Chemo | Placebo+Chemo | FDA parallel |
| KEYNOTE-407 | NCT02775435 | 2023 | PD-1 | Sq NSCLC | Pembrolizumab + Chemo | Placebo + Chemo | FDA cited |
| JAVELIN Renal 101 | NCT02684006 | 2020 | PD-L1+VEGF | RCC | Avelumab + Axitinib | Sunitinib | FDA cited |
| CHECKMATE-9ER | NCT03141177 | 2021 | PD-1+VEGF | RCC | Nivolumab + Cabozantinib | Sunitinib | FDA cited |
| CHECKMATE-214 | NCT02231749 | 2020 | PD-1+CTLA-4 | RCC | Nivolumab + Ipilimumab | Sunitinib | FDA cited |
| KEYNOTE-426 | NCT02853331 | 2020 | PD-1+VEGF | RCC | Pembrolizumab + Axitinib | Sunitinib | FDA cited |
| CLEAR | NCT02811861 | 2021 | PD-1+VEGF | RCC | Pembrolizumab + Lenvatinib | Sunitinib | FDA cited |
| CAPSTONE-1 | NCT03711305. | 2022 | PD-L1 | SCLC | Adebrelimab + Chemo | Placebo + Chemo | FDA parallel |
| IMpower133 | NCT02763579 | 2021 | PD-L1 | SCLC | Atezolizumab + Chemo | Placebo + Chemo | FDA cited |
| JAVELIN Bladder 100 | NCT02603432 | 2020 | PD-L1 | UC | Avelumab + Supportive care | Supportive care | FDA cited |

**Abbreviation:** BTC, biliary tract cancer; RCC, renal cell carcinoma; SCLC, small cell lung cancer; UC, urothelial carcinoma; Sq NSCLC, squamous non-small cell lung cancer; Non-Sq NSCLC, non-squamous non-small cell lung cancer; ESCC, esophageal squamous cell carcinoma; HER2^-^ GEA, HER2 negative gastroesophageal adenocarcinoma, including gastric, gastro-esophageal junction, and esophageal adenocarcinoma; HCC, hepatocellular carcinoma; MPM, malignant pleura mesothelioma; PD-1, Programmed Death-1; PD-L1, Programmed Death-Ligand 1; CTLA-4, Cytotoxic T-Lymphocyte-Associated Protein 4; VEGF, Vascular Endothelial Growth Factor; Chemo, Chemotherapy

FDA cited: Study which is cited by FDA regulatory policies.

FDA parallel: Study which has identical trial characteristics and conducted in parallel to FDA cited study.

# Table S4. Search information and where KMSubtraction was Implemented.

| **Clinical trial** | **Cancer Type** | **Treatment strategy**  **(Experimental vs Control)** | **PD-L1 test** | **Cutoff point** | **Outcome** | **Overall curve^1^** | **High PD-L1 curve^2^** | **Low PD-L1 curve^3^** | **KMSub-stracton Method** |
| --- | --- | --- | --- | --- | --- | --- | --- | --- | --- |
| KEYNOTE-590 | ESCC | Pembrolizumab + Chemo vs Chemo | CPS | 10 | OS | Yes | Yes | No | Yes |
| Checkmate 648 | ESCC | Nivolumab + Chemo  vs Chemo | TPS | 1 | OS | Yes | Yes | No | Yes |
| Checkmate 648 | ESCC | Nivolumab + Ipilimumab vs Chemo | TPS | 1 | OS | Yes | Yes | No | Yes |
| Checkmate 648 | ESCC | Nivolumab + Chemo  vs Chemo | TPS | 1 | PFS | Yes | Yes | No | Yes |
| Checkmate 648 | ESCC | Nivolumab + Ipilimumab vs Chemo | TPS | 1 | PFS | Yes | Yes | No | Yes |
| ORIENT-15 | ESCC | Sintilimab + Chemo  vs Chemo | CPS | 10 | OS | Yes | Yes | No | Yes |
| ORIENT-15 | ESCC | Sintilimab + Chemo  vs Chemo | CPS | 10 | PFS | Yes | Yes | No | Yes |
| JUPITER-06 | ESCC | Toripalimab + Chemo  vs Chemo | TPS | 1 | OS | Yes | Yes | Yes | No |
| JUPITER-06 | ESCC | Toripalimab + Chemo  vs Chemo | TPS | 1 | PFS | Yes | Yes | Yes | No |
| ESCORT-1st | ESCC | Camrelizumab + Chemo vs Chemo | TPS | 1 | OS | Yes | Yes | Yes | No |

| **Clinical trial** | **Cancer Type** | **Treatment strategy**  **(Experimental vs Control)** | **PD-L1 test** | **Cutoff point** | **Outcome** | **Overall curve^1^** | **High PD-L1 curve^2^** | **Low PD-L1 curve^3^** | **KMSub-stracton Method** |
| --- | --- | --- | --- | --- | --- | --- | --- | --- | --- |
| ESCORT-1st | ESCC | Camrelizumab + Chemo vs Chemo | TPS | 1 | PFS | Yes | Yes | Yes | No |
| RATIONALE-306 | ESCC | Tislelizumab + Chemo vs Chemo | TAP | 10 | OS | Yes | Yes | Yes | No |
| CheckMate-649 | HER2^-^ GEA | Nivolumab + Chemo  vs Chemo | CPS | 1 | OS | Yes | Yes | No | Yes |
| CheckMate-649 | HER2^-^ GEA | Nivolumab + Chemo  vs Chemo | CPS | 5 | OS | Yes | Yes | No | Yes |
| CheckMate-649 | HER2^-^ GEA | Nivolumab + Chemo  vs Chemo | CPS | 1 | PFS | Yes | Yes | No | Yes |
| CheckMate-649 | HER2^-^ GEA | Nivolumab + Chemo  vs Chemo | CPS | 5 | PFS | Yes | Yes | No | Yes |
| KEYNOTE-859 | HER2^-^ GEA | Pembrolizumab + Chemo vs Chemo | CPS | 1 | OS | Yes | Yes | No | Yes |
| KEYNOTE-859 | HER2^-^ GEA | Pembrolizumab + Chemo vs Chemo | CPS | 10 | OS | Yes | Yes | No | Yes |
| KEYNOTE-859 | HER2^-^ GEA | Pembrolizumab + Chemo vs Chemo | CPS | 1 | PFS | Yes | Yes | No | Yes |
| KEYNOTE-859 | HER2^-^ GEA | Pembrolizumab + Chemo vs Chemo | CPS | 10 | PFS | Yes | Yes | No | Yes |
| ORIENT-16 | HER2^-^ GEA | Sintilimab + Chemo  vs Chemo | CPS | 5 | OS | Yes | Yes | No | Yes |

| **Clinical trial** | **Cancer Type** | **Treatment strategy**  **(Experimental vs Control)** | **PD-L1 test** | **Cutoff point** | **Outcome** | **Overall curve^1^** | **High PD-L1 curve^2^** | **Low PD-L1 curve^3^** | **KMSub-stracton Method** |
| --- | --- | --- | --- | --- | --- | --- | --- | --- | --- |
| ORIENT-16 | HER2^-^ GEA | Sintilimab + Chemo  vs Chemo | CPS | 5 | PFS | Yes | Yes | No | Yes |
| KEYNOTE-062 | HER2^-^ GEA | Pembrolizumab + Chemo vs Chemo | CPS | 1 | OS | Yes | Yes | No | Yes |
| KEYNOTE-062 | HER2^-^ GEA | Pembrolizumab+ Chemo vs Chemo | CPS | 10 | OS | Yes | Yes | No | Yes |
| KEYNOTE-062 | HER2^-^ GEA | Pembrolizumab+ Chemo vs Chemo | CPS | 1 | PFS | Yes | Yes | No | Yes |
| KEYNOTE-062 | HER2^-^ GEA | Pembrolizumab+ Chemo vs Chemo | CPS | 10 | PFS | Yes | Yes | No | Yes |
| KEYNOTE-966 | BTC | Pembrolizumab + Chemo vs Chemo | CPS | 1 | OS | Yes | No | No | No |
| TOPAZ-1 | BTC | Durvalumab + Chemo vs Chemo | TAP | 1% | OS | Yes | No | No | No |

**Abbreviation:** ESCC, Esophageal squamous cell carcinoma; HER2^-^ GEA, HER2 negative Gastroesophageal adenocarcinoma, including gastric, gastro-esophageal junction, and esophageal adenocarcinoma; BTC, Biliary tract cancer; Chemo, Chemotherapy; OS, Overall survival; PFS, Progression free survival; PD-L1, Programmed death-ligand 1; TPS, Tumor proportion Score; CPS, Combined positive score; TAP, Tumor area positivity.

1. Overall curve: Kaplan-Meier plot of OS or PFS in the overall population;
2. High PD-L1 curve: Kaplan-Meier plot of OS or PFS in patients with high PD-L1 expression;
3. Low PD-L1 curve: Kaplan-Meier plot of OS or PFS in patients with low PD-L1 expression.

# Figure S1. Risk of bias and quality assessment of RCTs using RoB2

**Abbreviations:** A. Random sequence generation; B. Allocation concealment; C. Blinding of participants and personnel; D. Blinding of outcome assessment; E. Incomplete outcome data; F. Selective reporting; G. Other bias

# Figure S2. Random forest for overall survival (OS) outcome in high programmed death ligand (PD-L1) subgroups by tumor type.

Horizontal lines represent the 95% confidence interval (CI). The meta-analysis was conducted by HKSJ method.

**Abbreviations:** BTC, Biliary tract cancer; ESCC, Esophageal cell carcinoma; GEA, Gastroesophageal adenocarcinoma, including gastric, gastro-esophageal junction, and esophageal adenocarcinoma; HCC, Hepatocellular carcinoma; MPM, Malignant pleura mesothelioma; NSCLC, Non-small cell lung cancer; RCC, Renal cell carcinoma; SCLC, Small cell lung cancer; UC, Urothelial carcinoma; PD-L1, Programmed death-ligand 1; TPS, Tumor Proportion Score; CPS, Combined positive score; TAP, Tumor Area Positivity.

1. Indicates the hazard ratio of nivolumab + chemotherapy arm compared to chemotherapy in CheckMate 648;

2. Indicates the hazard ratio of nivolumab + ipilimumab arm compared to chemotherapy in CheckMate 648;

3. Indicates the hazard ratio of atezolizumab + chemotherapy arm compared to bevacizumab + chemotherapy in IMpower 150;

4. Indicates the hazard ratio of atezolizumab + bevacizumab + chemotherapy arm compared to bevacizumab + chemotherapy in IMpower 150;

5. Indicates the hazard ratio of durvalumab + chemotherapy arm compared to chemotherapy in POSEIDON;

6. Indicates the hazard ratio of durvalumab + tremelimumab + chemotherapy arm compared to chemotherapy in POSEIDON.

# Figure S3. Sensitivity test for low programmed death ligand (PD-L1) subgroups

# Figure S4. Sensitivity test for high programmed death ligand (PD-L1) subgroups

# Figure S5. Comparison of original and reconstructed figures for overall and high/ low programmed death ligand (PD-L1) subgroups

| **Study** | **Original figure** | **Reconstructed figure** |
| --- | --- | --- |
| **KEYNOTE-590**  ESCC  OS  Overall cohort  PMID: 34454674  Figure 2B  Therapy:  Pembrolizumab plus chemotherapy vs Chemotherapy | 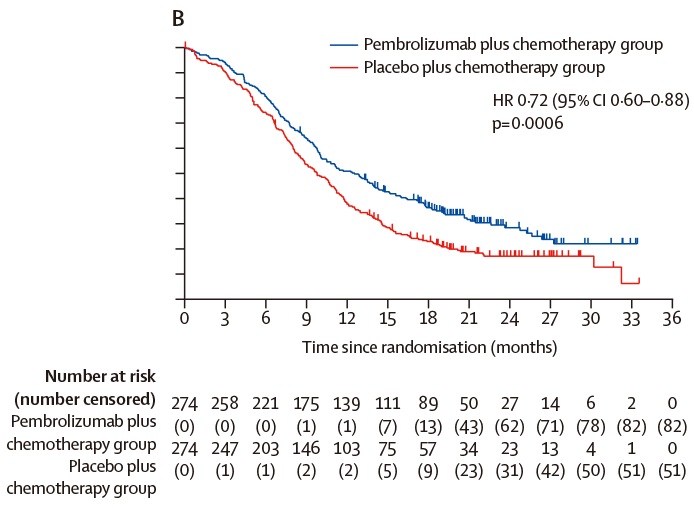 | 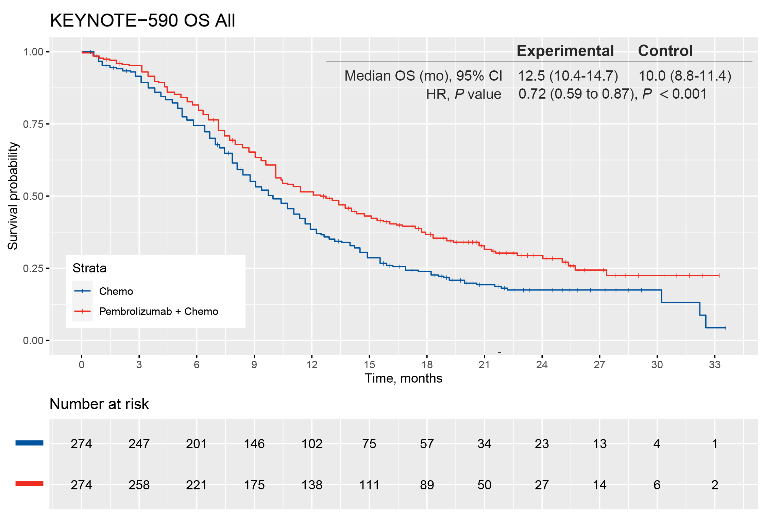 |
| **KEYNOTE-590**  ESCC  OS  CPS>=10  PMID: 34454674  Figure 2A  Therapy:  Pembrolizumab plus chemotherapy vs Chemotherapy | 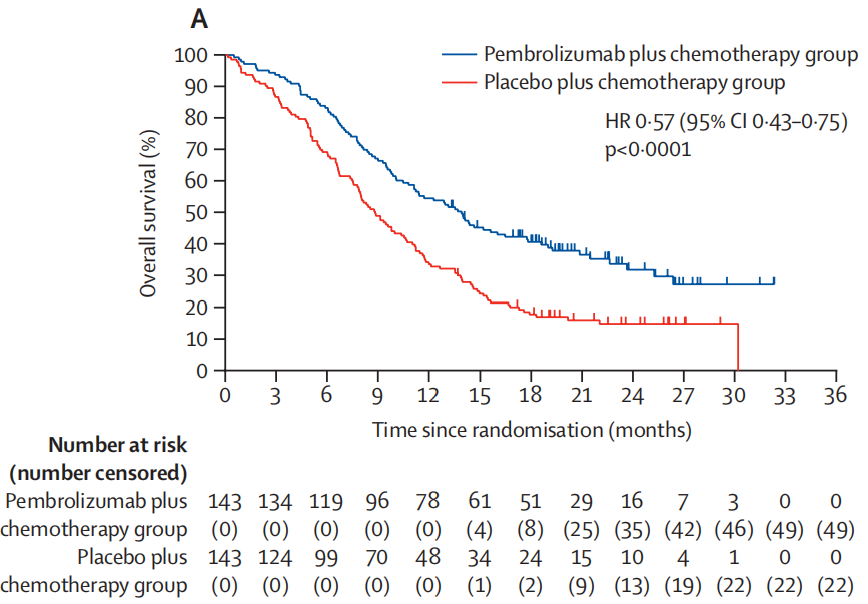 | 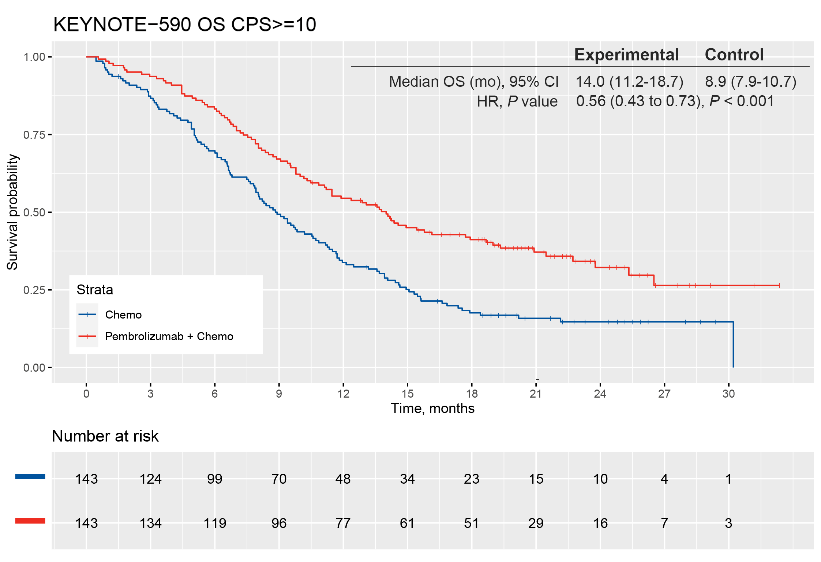 |
| **Study** | **Original figure** | **Reconstructed figure** |
| **Checkmate-648**  ESCC  OS  Overall cohort  PMID: 35108470  Figure 1B  Therapy: Nivolumab plus Chemotherapy vs Chemotherapy | 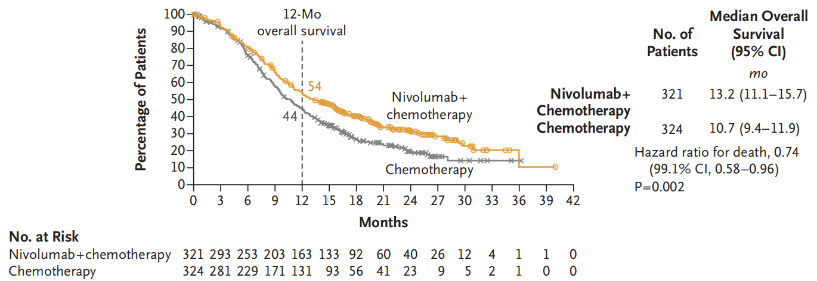 | 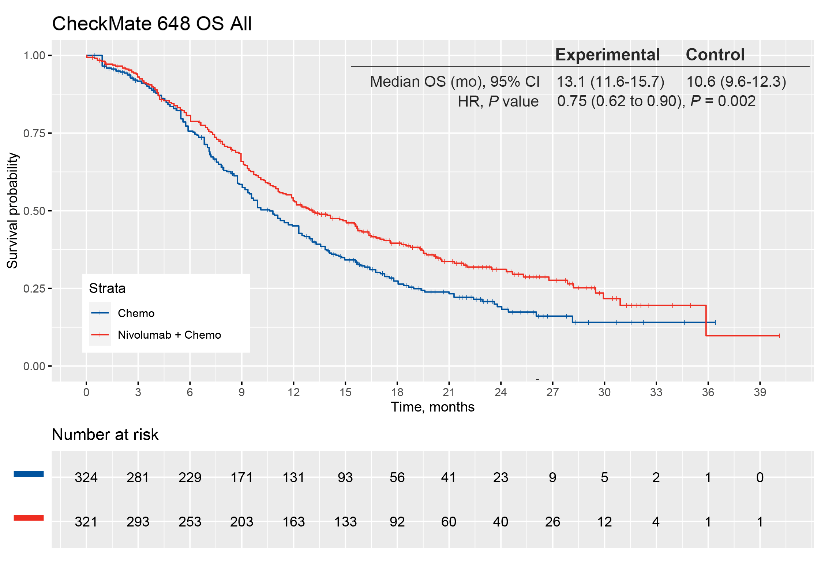 |
| **Checkmate-648**  ESCC  OS  TPS>=1%  PMID: 35108470  Figure 1A  Therapy: Nivolumab plus Chemotherapy vs Chemotherapy | 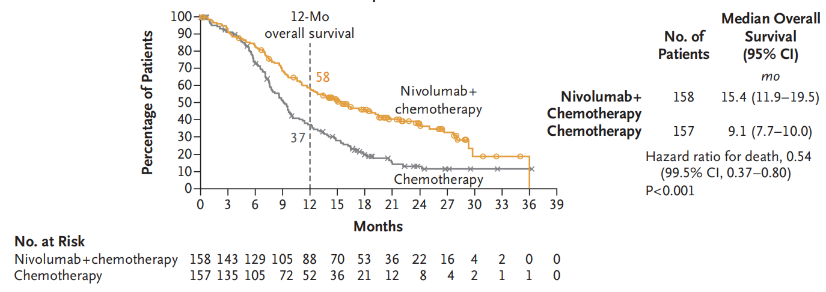 | 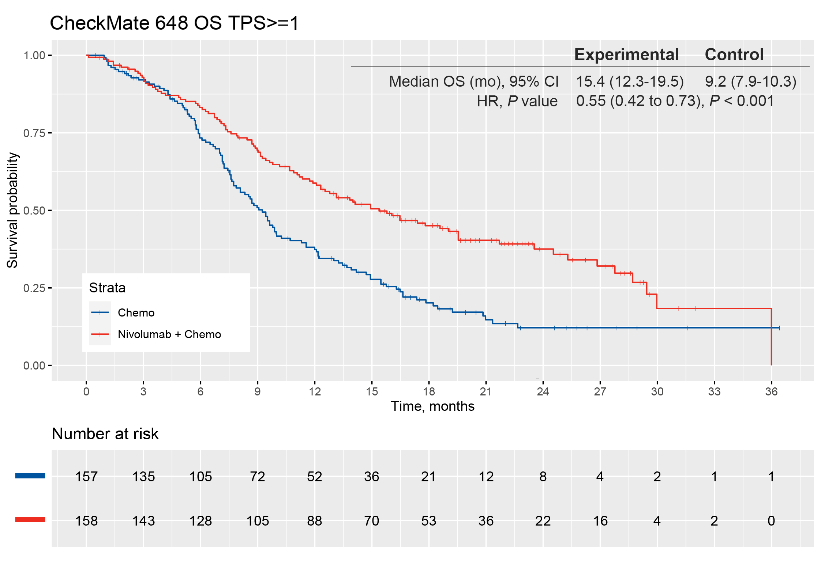 |
| **Study** | **Original figure** | **Reconstructed figure** |
| **Checkmate-648**  ESCC  OS  Overall cohort  PMID: 35108470  Figure 2B  Therapy: Nivolumab plus ipilimumab vs Chemotherapy | 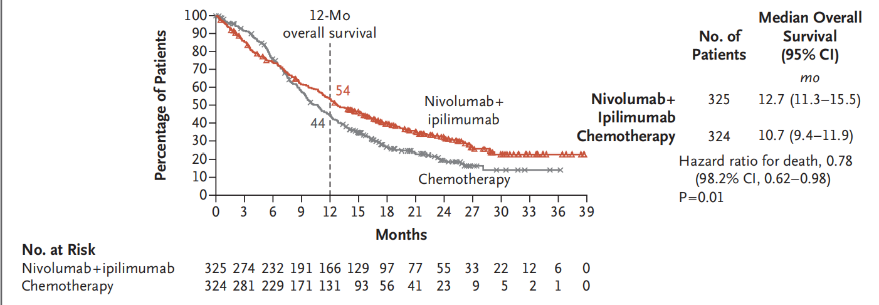 | 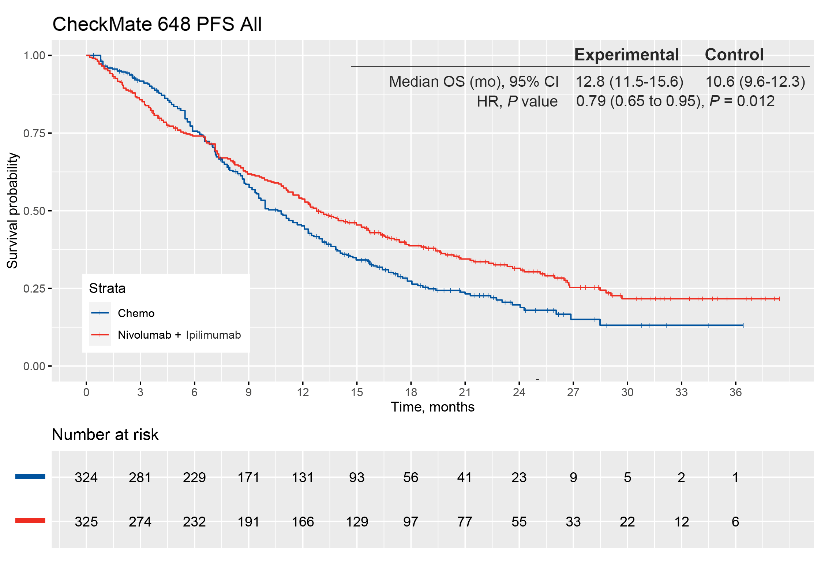 |
| **Checkmate-648**  ESCC  OS  TPS>=1%  PMID: 35108470  Figure 2A  Therapy: Nivolumab plus ipilimumab vs Chemotherapy | 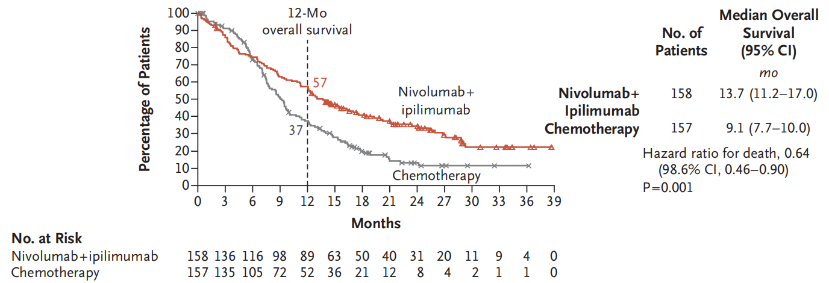 | 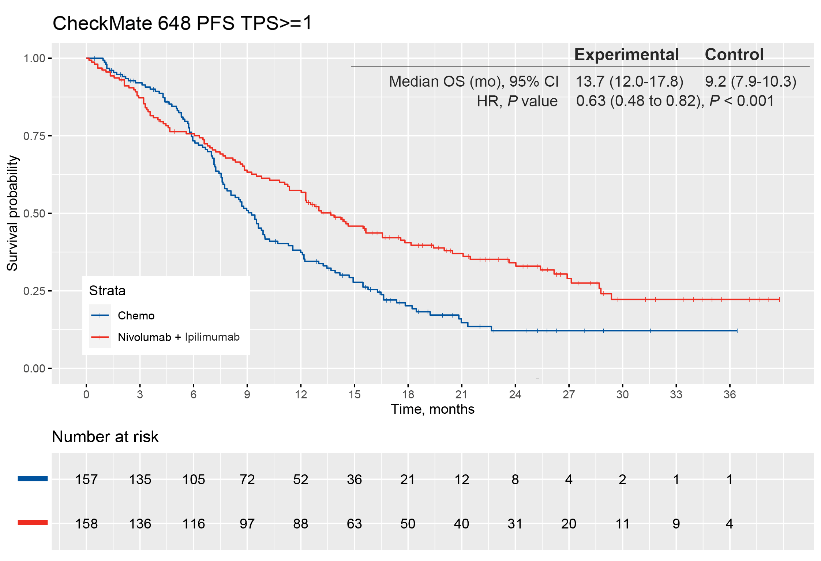 |
| **Study** | **Original figure** | **Reconstructed figure** |
| **Checkmate-648**  ESCC  PFS  Overall cohort  PMID: 35108470  Figure 1D  Therapy: Nivolumab plus Chemotherapy vs Chemotherapy | 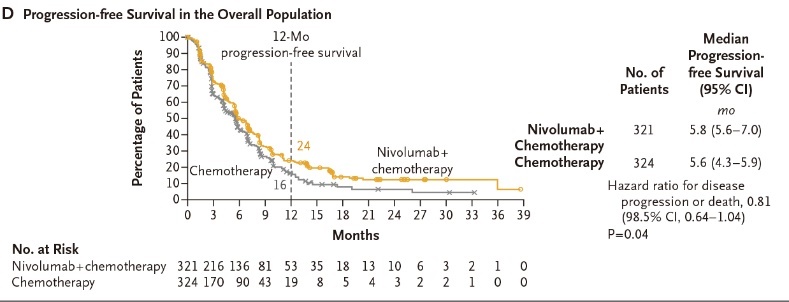 | 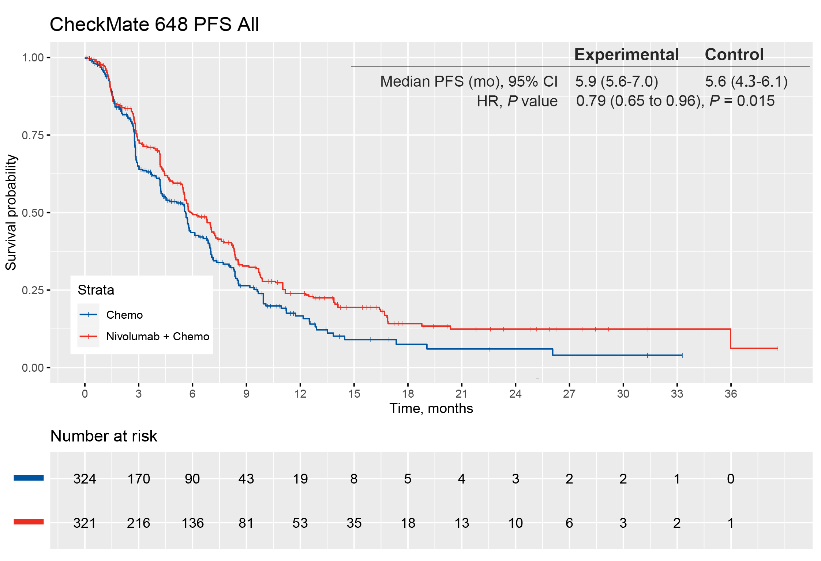 |
| **Checkmate-648**  ESCC  PFS  TPS>=1%  PMID: 35108470  Figure 1C  Therapy: Nivolumab plus Chemotherapy vs Chemotherapy | 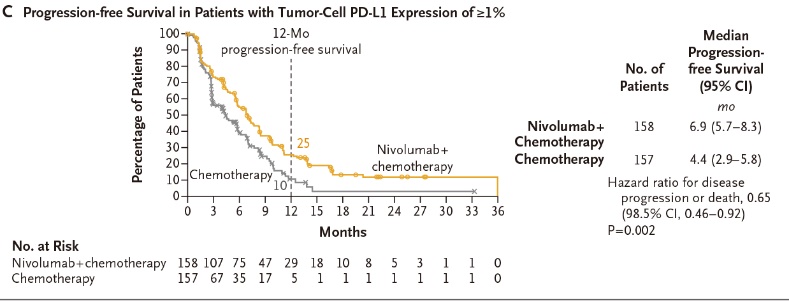 | 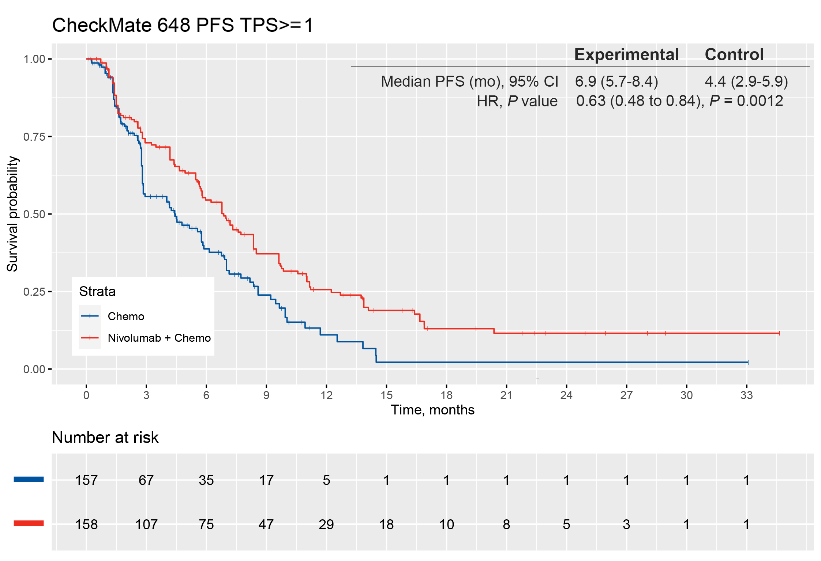 |
| **Study** | **Original figure** | **Reconstructed figure** |
| **Checkmate-648**  ESCC  PFS  Overall cohort  PMID: 35108470  Figure 2D  Therapy: Nivolumab plus ipilimumab vs Chemotherapy | 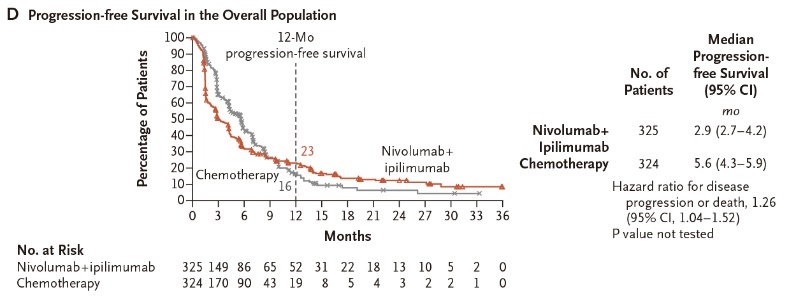 | 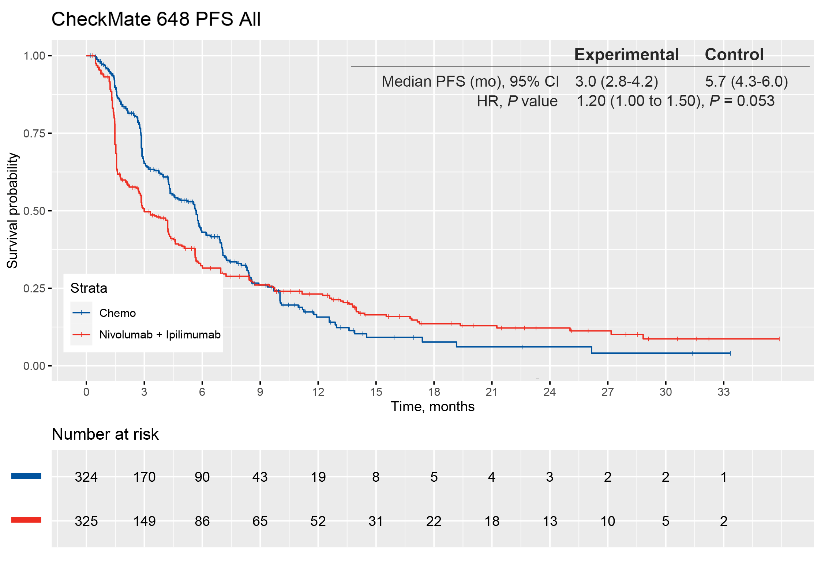 |
| **Checkmate-648**  ESCC  PFS  TPS>=1%  PMID: 35108470  Figure 2C  Therapy: Nivolumab plus ipilimumab vs Chemotherapy | 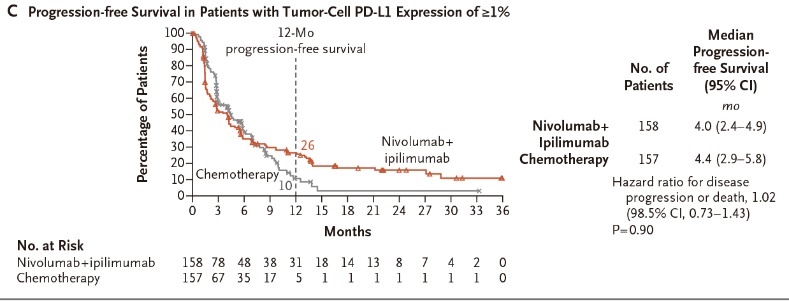 | 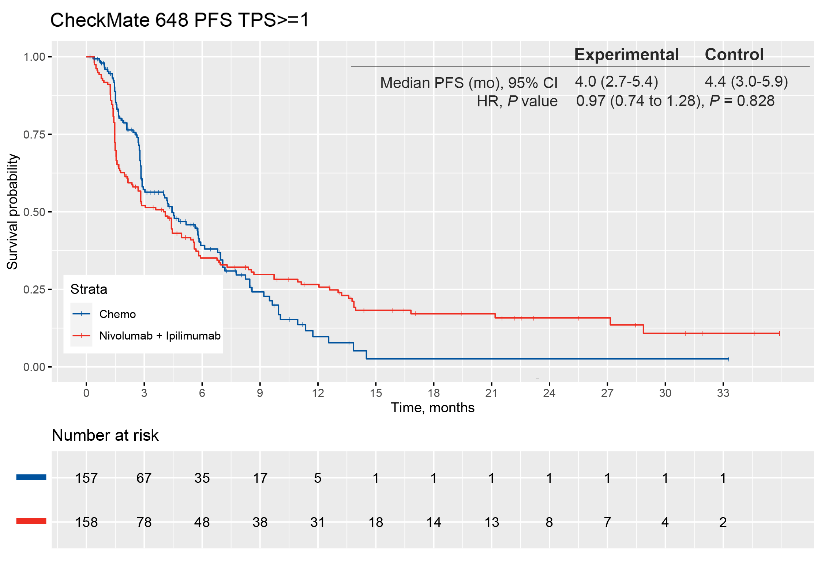 |
| **Study** | **Original figure** | **Reconstructed figure** |
| **ORIENT-15**  ESCC  OS  Overall cohort  PMID: 35440464  Figure 2 top  Therapy: Sintilimab plus chemotherapy vs Placebo + chemotherapy | 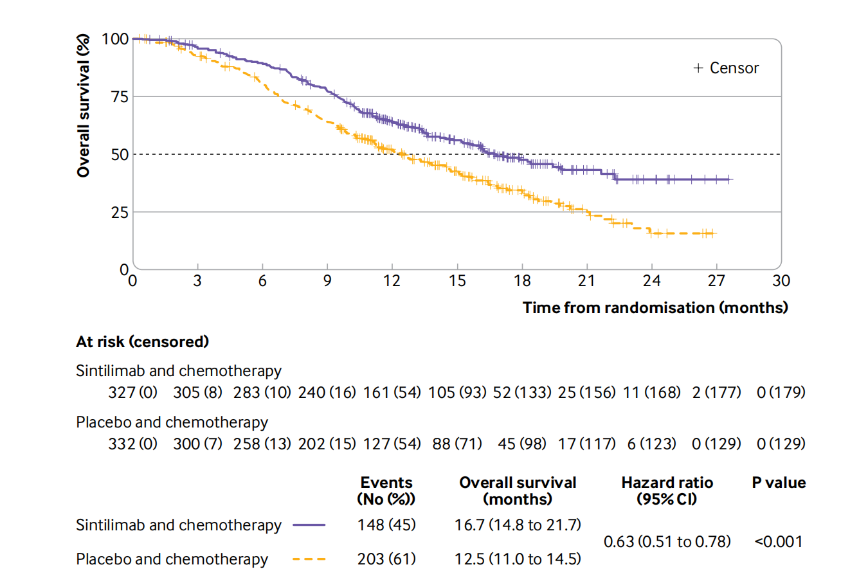 | 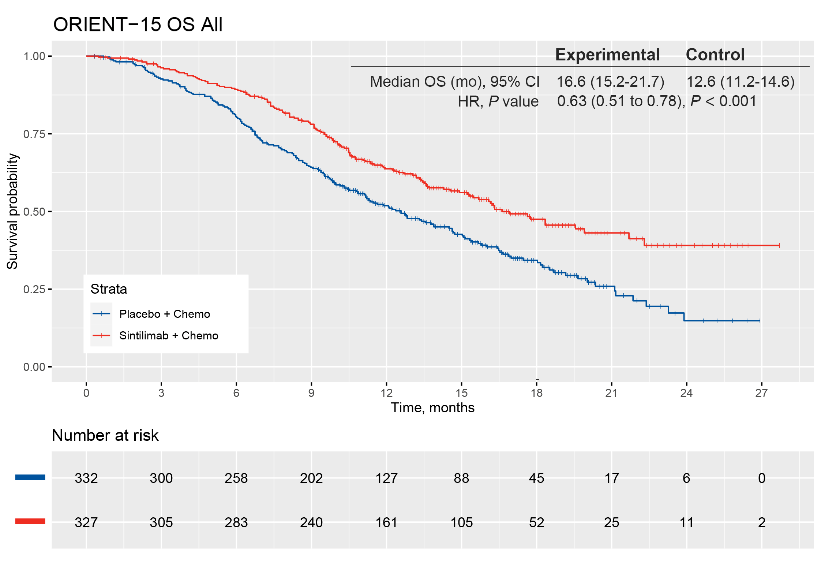 |
| **ORIENT-15**  ESCC  OS  CPS>=10  PMID: 35440464  Figure 2 bottom  Therapy: Sintilimab plus chemotherapy vs Placebo plus chemotherapy | 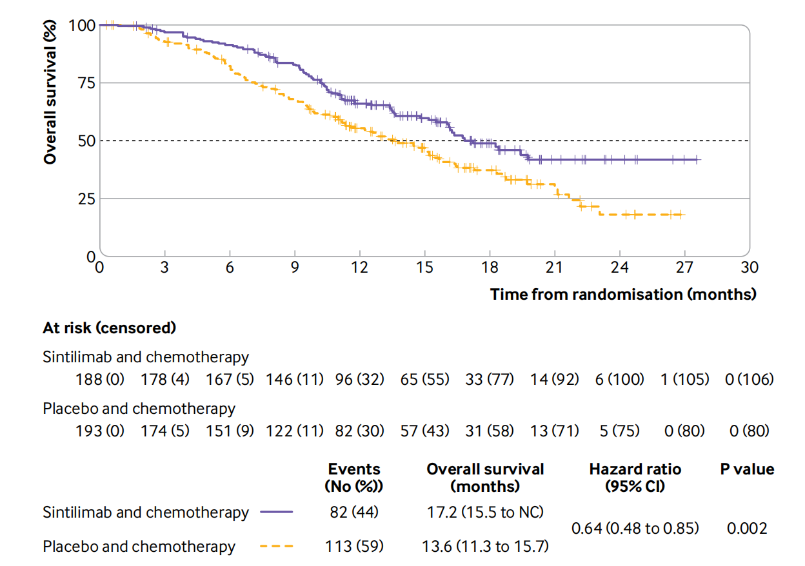 | 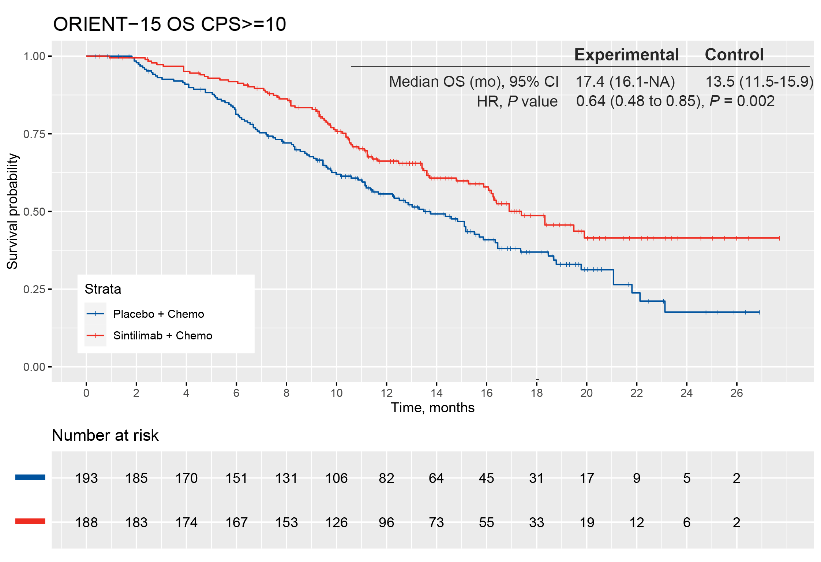 |
| **Study** | **Original figure** | **Reconstructed figure** |
| **ORIENT-15**  ESCC  PFS  Overall cohort  PMID: 35440464  Figure 4 top  Therapy: Sintilimab plus chemotherapy vs Placebo plus chemotherapy | 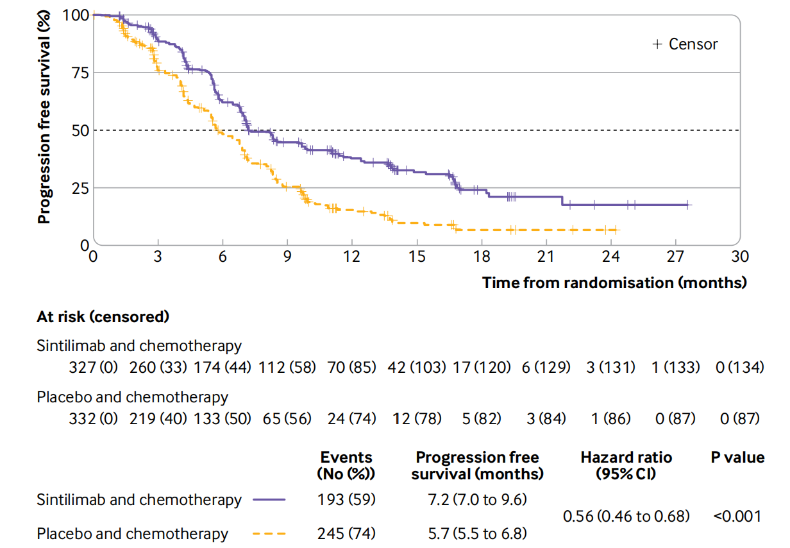 | 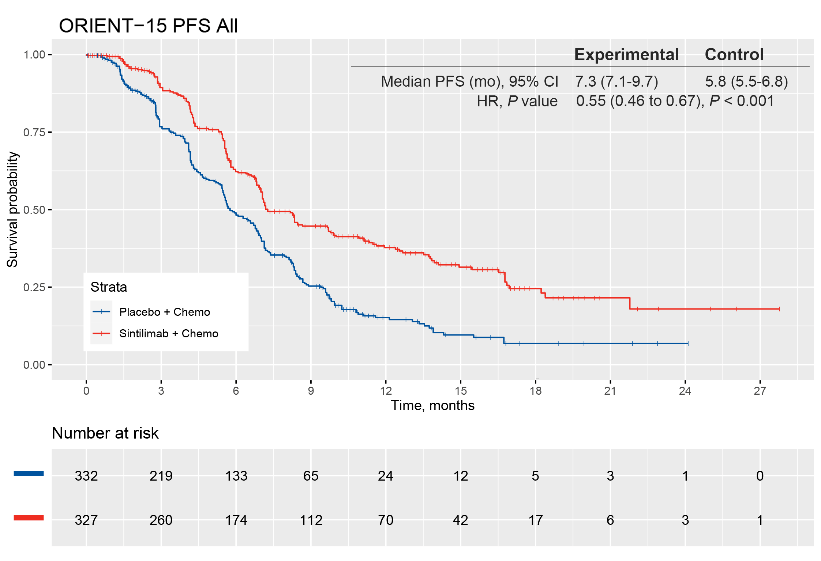 |
| **ORIENT-15**  ESCC  PFS  CPS>=10  PMID: 35440464  Figure 4 bottom  Therapy: Sintilimab + chemotherapy vs Placebo + chemotherapy | 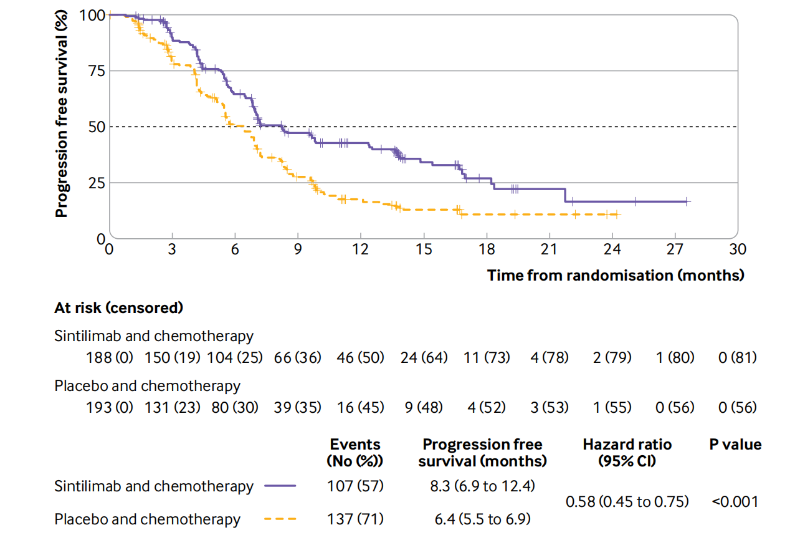 | 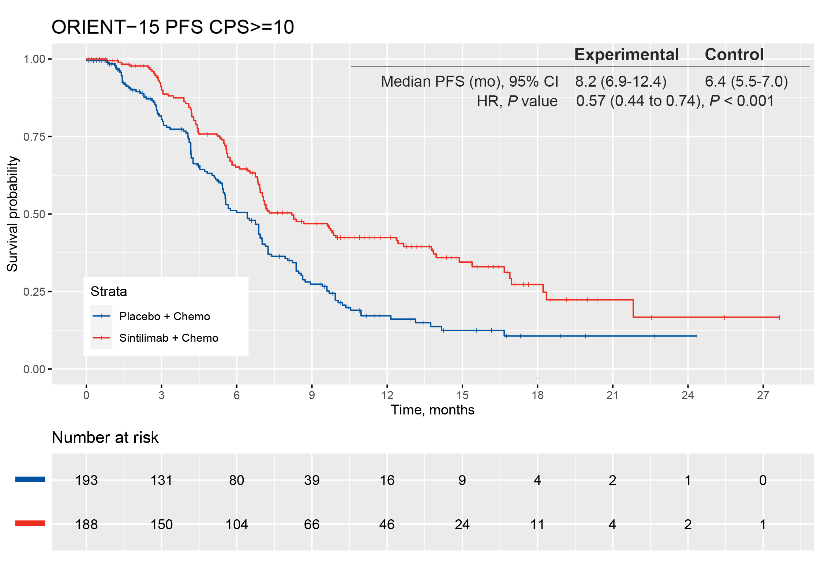 |
| **Study** | **Original figure** | **Reconstructed figure** |
| **JUPITER-06**  ESCC  OS  PD-L1 TPS<1  PMID: 36473145  Figure 1D  Therapy: Toripalimab plus chemotherapy vs placebo plus chemotherapy | 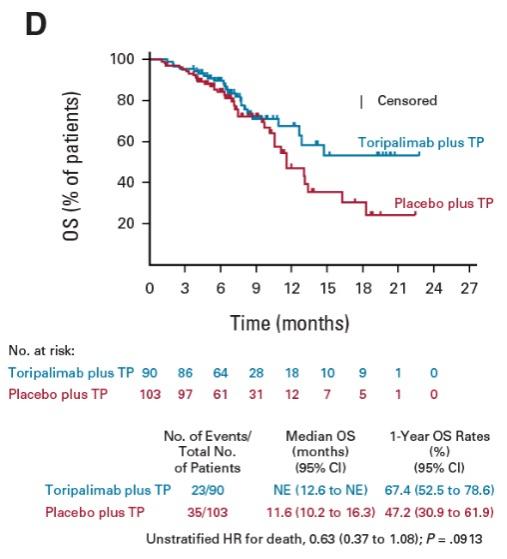 | 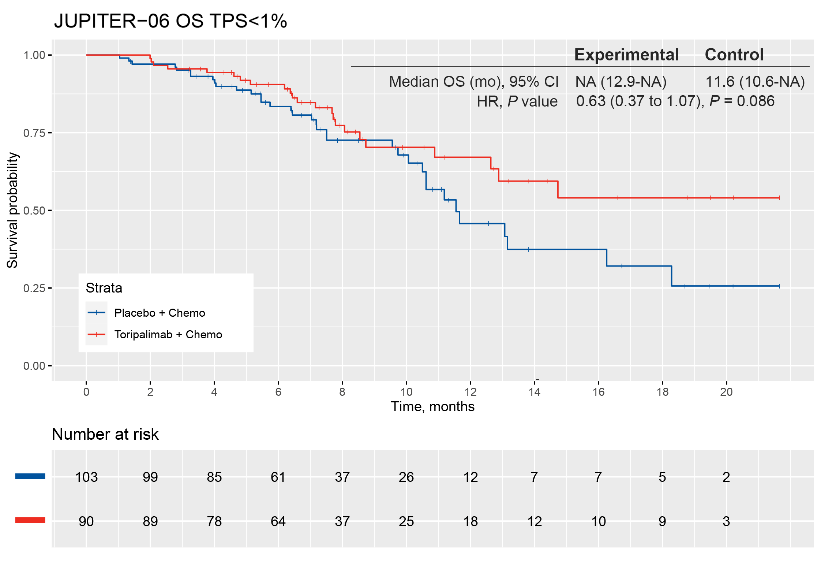 |
| **JUPITER-06**  ESCC  PFS  PD-L1 TPS<1  PMID: 36473145  Figure 1B  Therapy: Toripalimab plus chemotherapy vs Placebo plus chemotherapy | 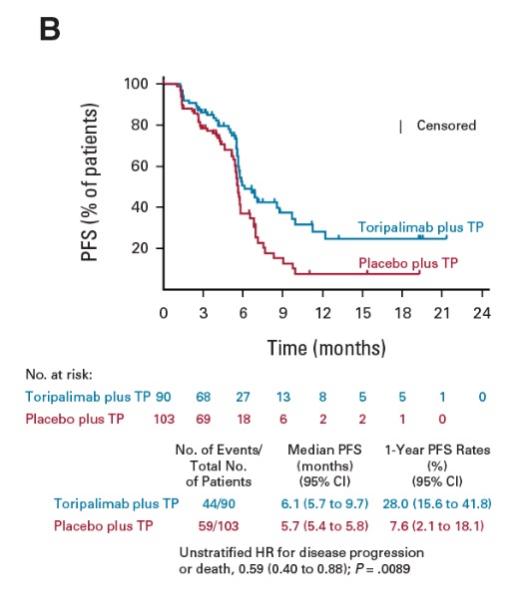 | 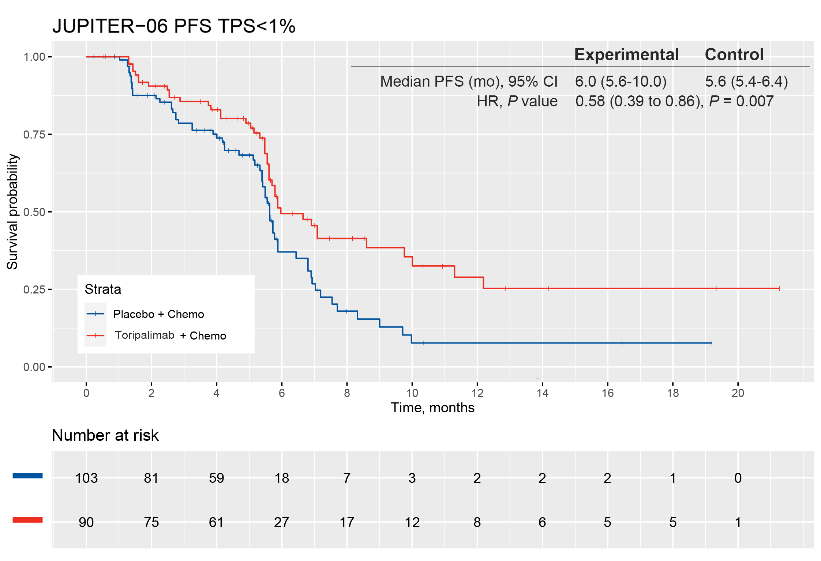 |
| **Study** | **Original figure** | **Reconstructed figure** |
| **ESCORT-1st**  ESCC  OS  PD-L1 TPS<1%  PMID: 34519801  eFigure 5  Therapy: Camrelizumab plus chemotherapy vs Placebo + chemotherapy | 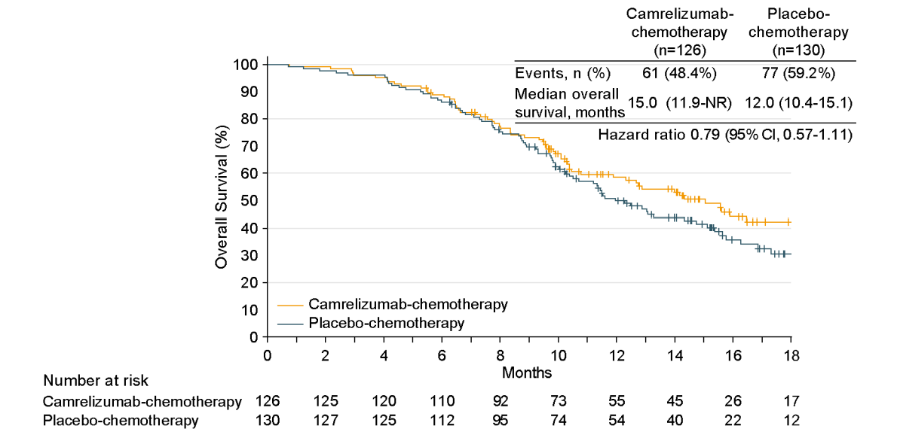 | 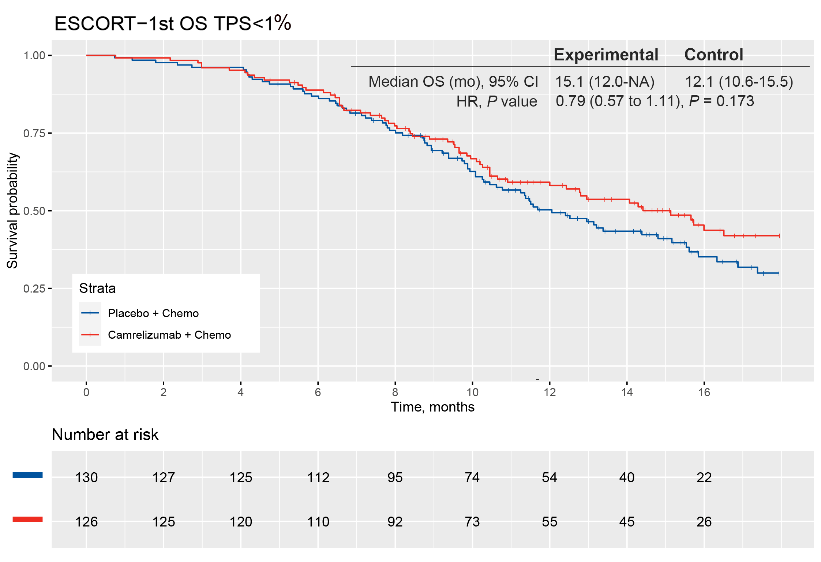 |
| **ESCORT-1st**  ESCC  PFS  TPS<1%  PMID: 34519801  eFigure 7  Therapy: Camrelizumab plus chemotherapy vs Placebo + chemotherapy | 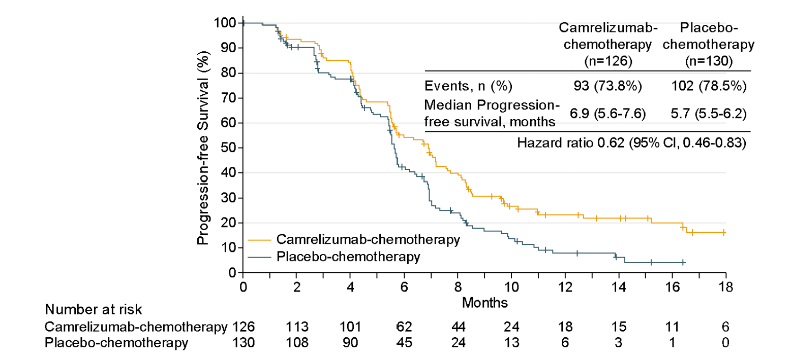 | 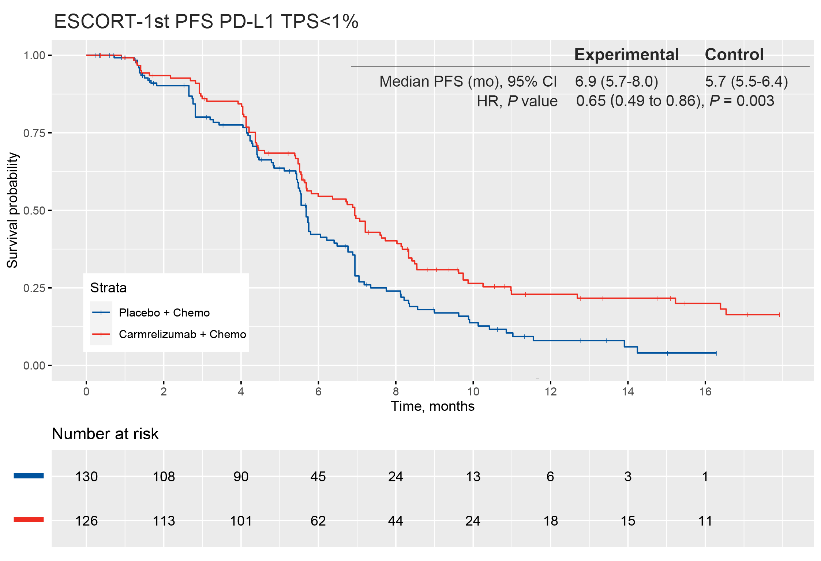 |
| **Study** | **Original figure** | **Reconstructed figure** |
| **Checkmate-649**  HER2 negative GEA  OS  Overall cohort  PMID: 34102137  Figure 2C  Therapy: Nivolumab plus chemotherapy vs Chemotherapy | 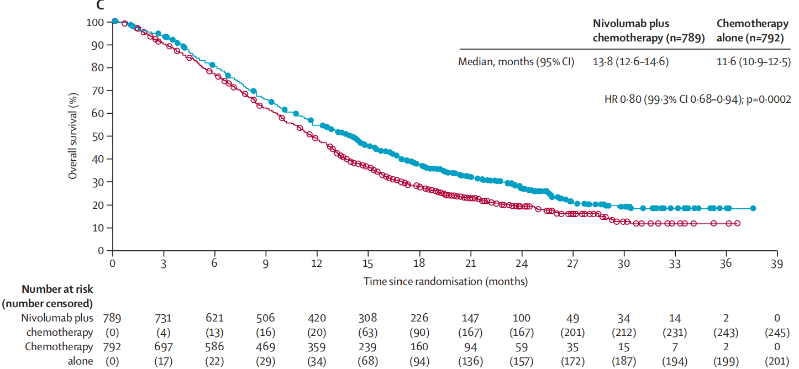 | 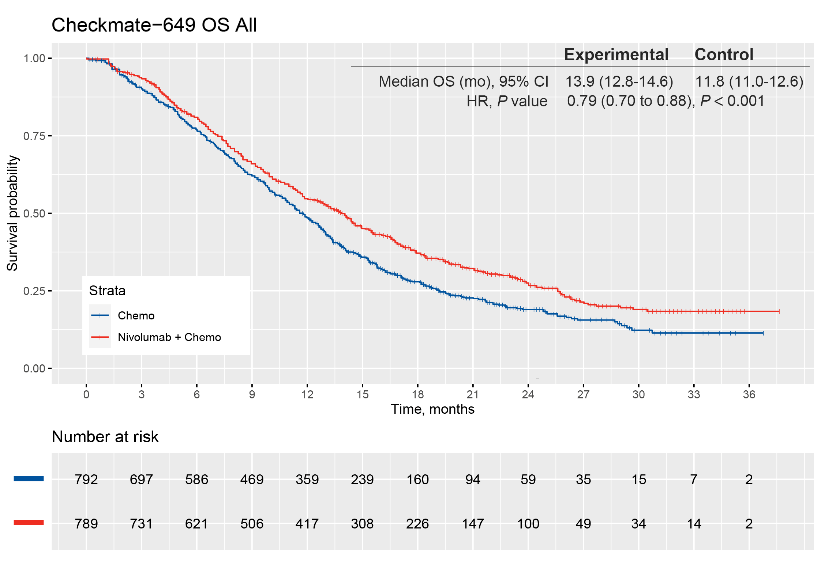 |
| **Checkmate-649**  HER2 negative GEA  OS  CPS>=1  PMID: 34102137  Figure 2B  Therapy: Nivolumab plus chemotherapy vs Chemotherapy | 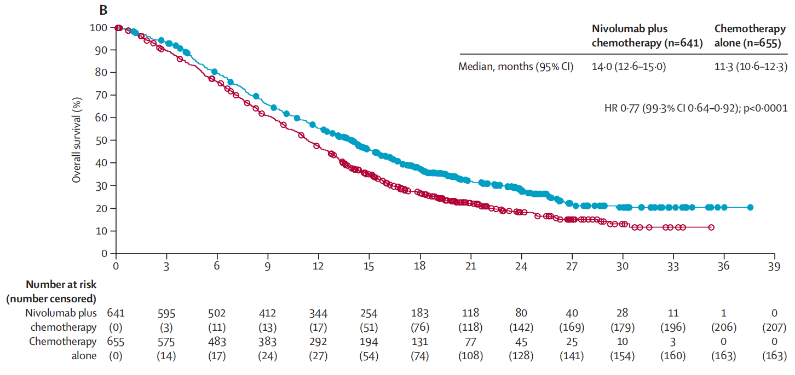 | 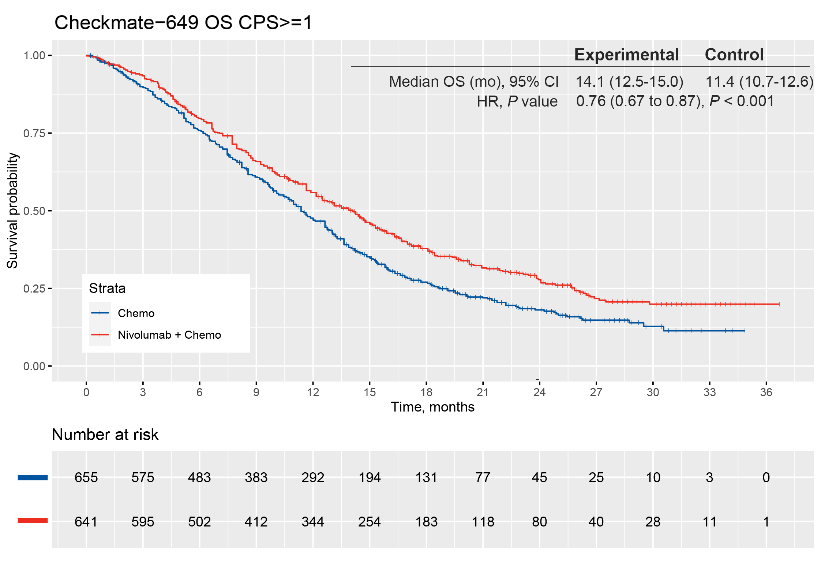 |
| **Study** | **Original figure** | **Reconstructed figure** |
| **Checkmate-649**  HER2 negative GEA  OS  CPS>=5  PMID: 34102137  Figure 2A  Therapy: Nivolumab plus chemotherapy vs Chemotherapy | 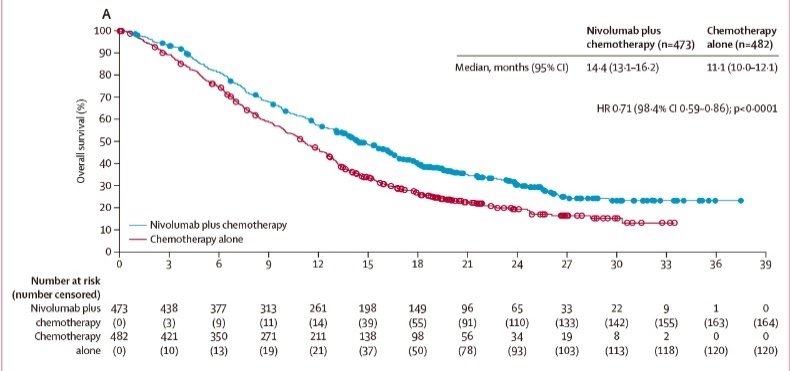 | 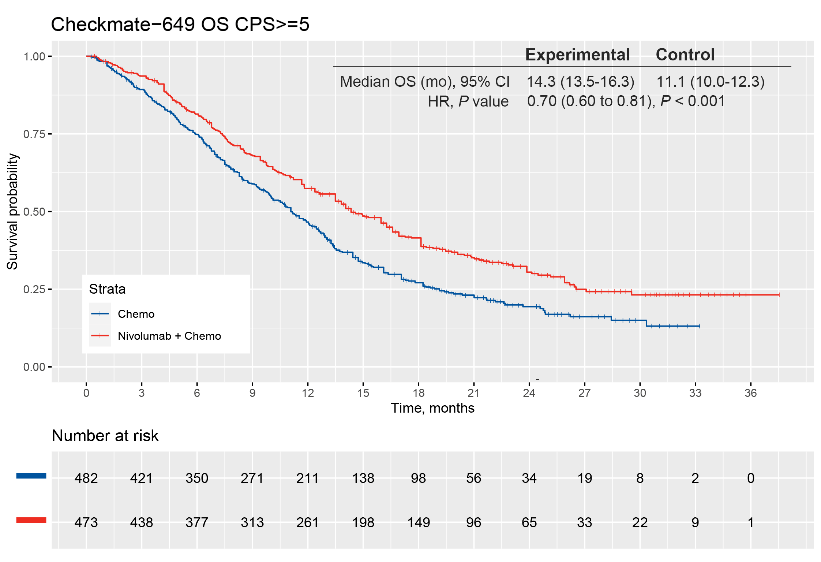 |
| **Checkmate-649**  HER2 negative GEA  PFS  Overall cohort  PMID: 34102137  Figure 3C  Therapy: Nivolumab plus chemotherapy vs Chemotherapy | 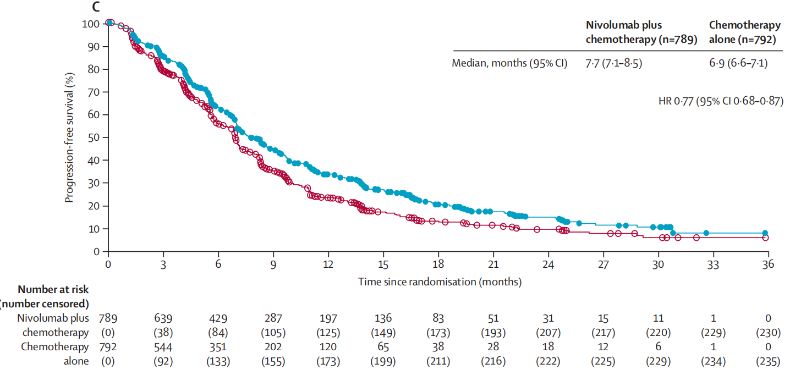 | 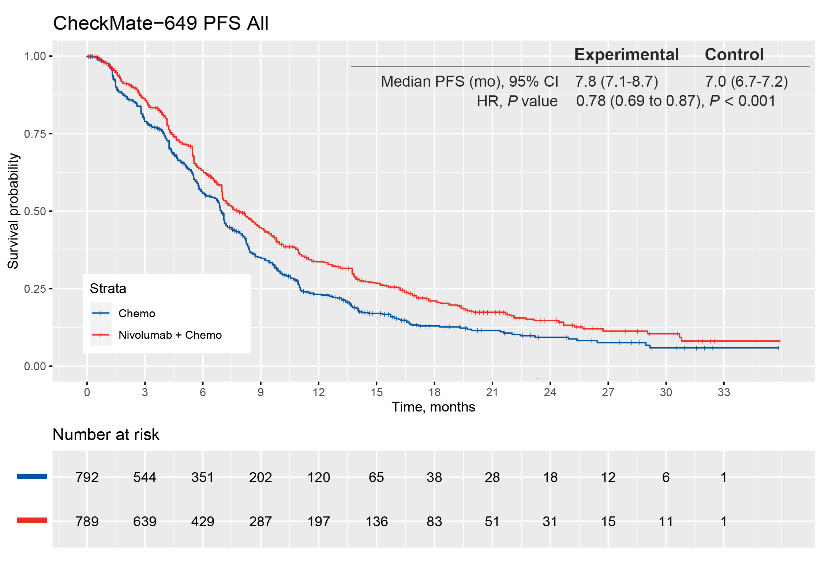 |
| **Study** | **Original figure** | **Reconstructed figure** |
| **Checkmate-649**  HER2 negative GEA  PFS  CPS>=1  PMID: 34102137  Figure 3B  Therapy: Nivolumab plus chemotherapy vs Chemotherapy | 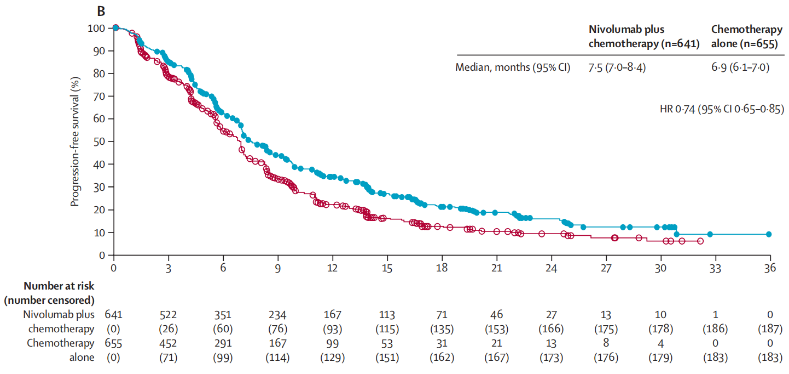 | 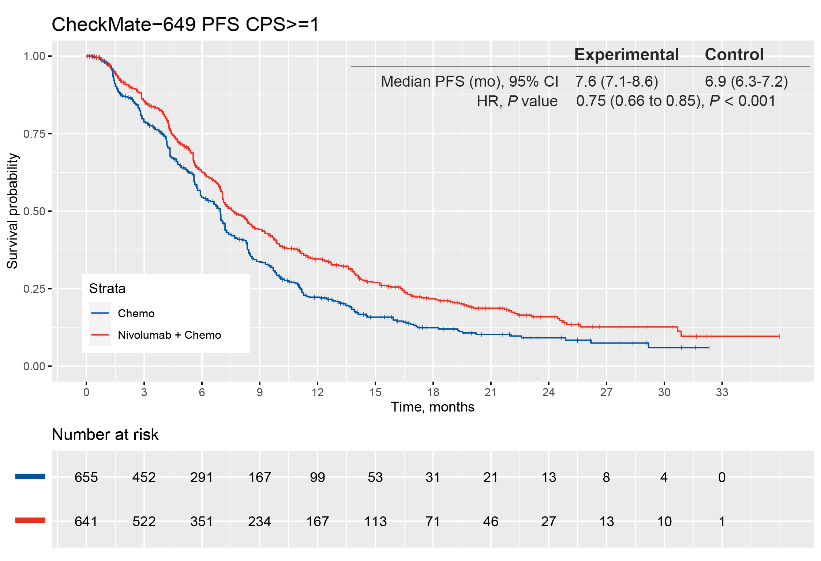 |
| **CheckMate-649**  HER2 negative GEA  PFS  CPS>=5  PMID: 34102137  Figure 3A  Therapy: Nivolumab plus chemotherapy vs Chemotherapy | 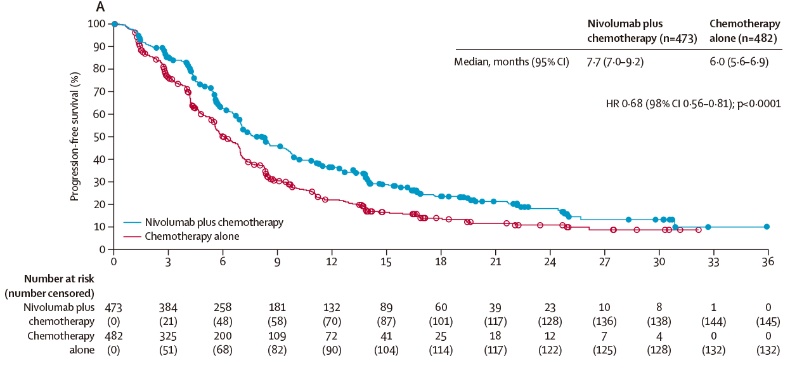 | 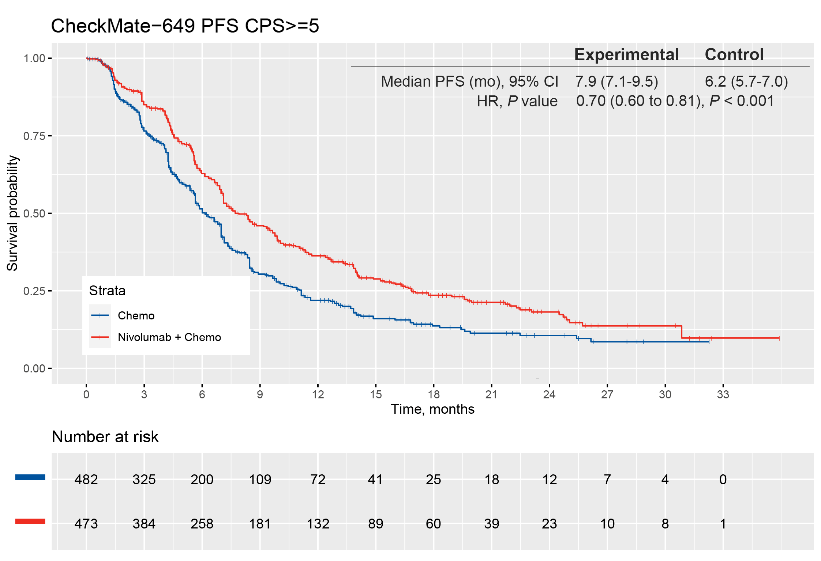 |
| **Study** | **Original figure** | **Reconstructed figure** |
| **KEYNOTE-859**  HER2 negative GEA  OS  Overall cohort  PMID: 37875143  Figure 2A  Therapy: Pembrolizumab plus chemotherapy vs Placebo plus chemotherapy | 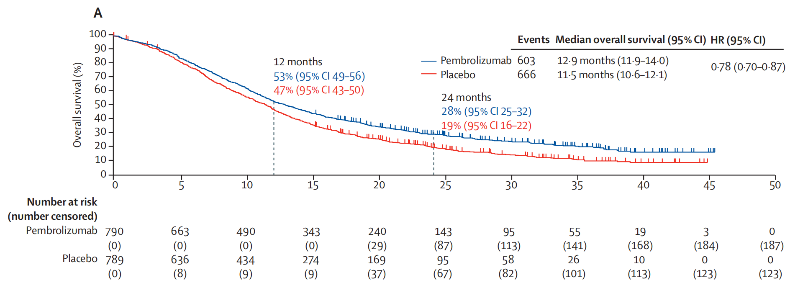 | 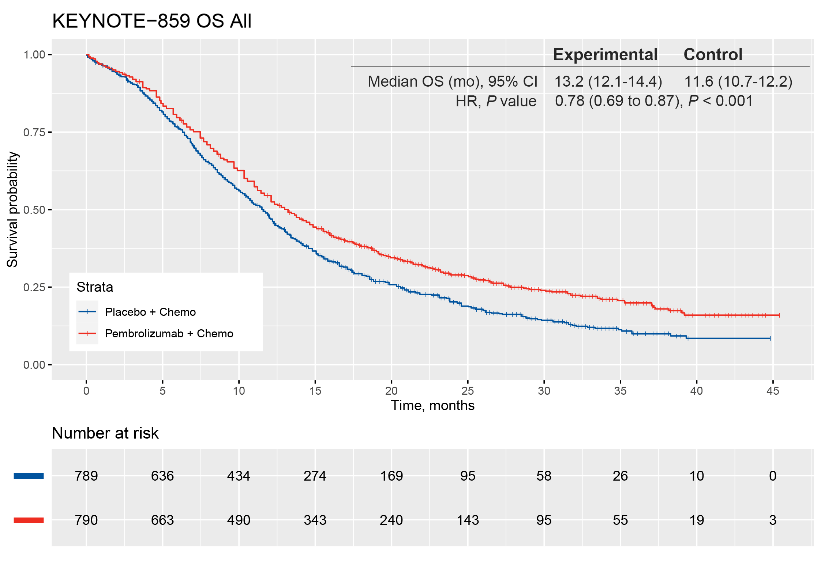 |
| **KEYNOTE-859**  HER2 negative GEA  OS  CPS>=1  PMID: 37875143  Figure 2B  Therapy: Pembrolizumab plus chemotherapy vs Placebo plus chemotherapy | 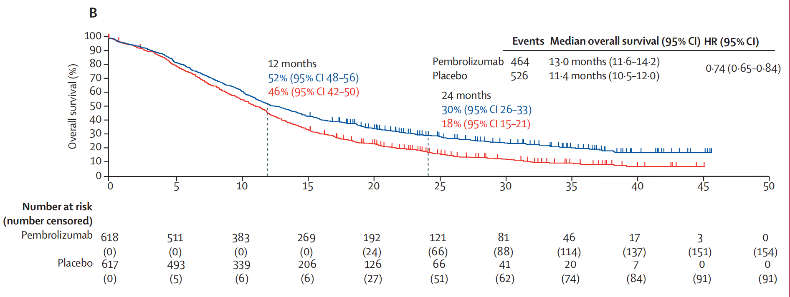 | 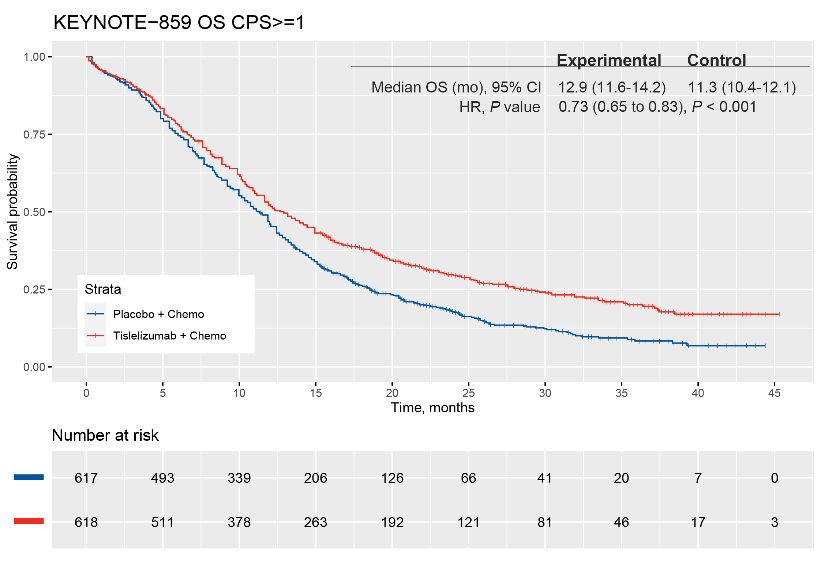 |
| **Study** | **Original figure** | **Reconstructed figure** |
| **KEYNOTE-859**  HER2 negative GEA  OS  CPS>=10  PMID: 37875143  Figure 2C  Therapy: Pembrolizumab plus chemotherapy vs Placebo plus chemotherapy | 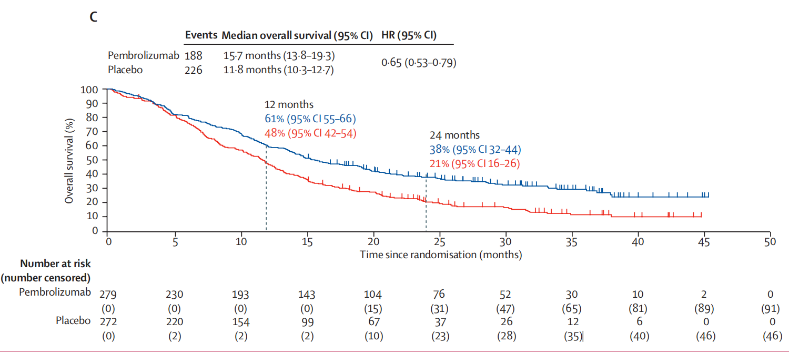 | 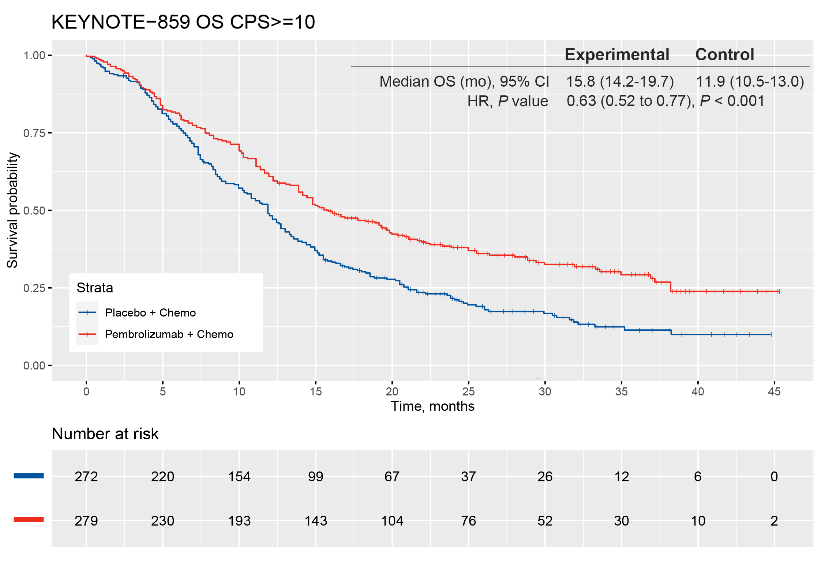 |
| **KEYNOTE-859**  HER2 negative GEA  PFS  Overall cohort  PMID: 37875143  Figure 3A  Therapy: Pembrolizumab plus chemotherapy vs Placebo plus chemotherapy | 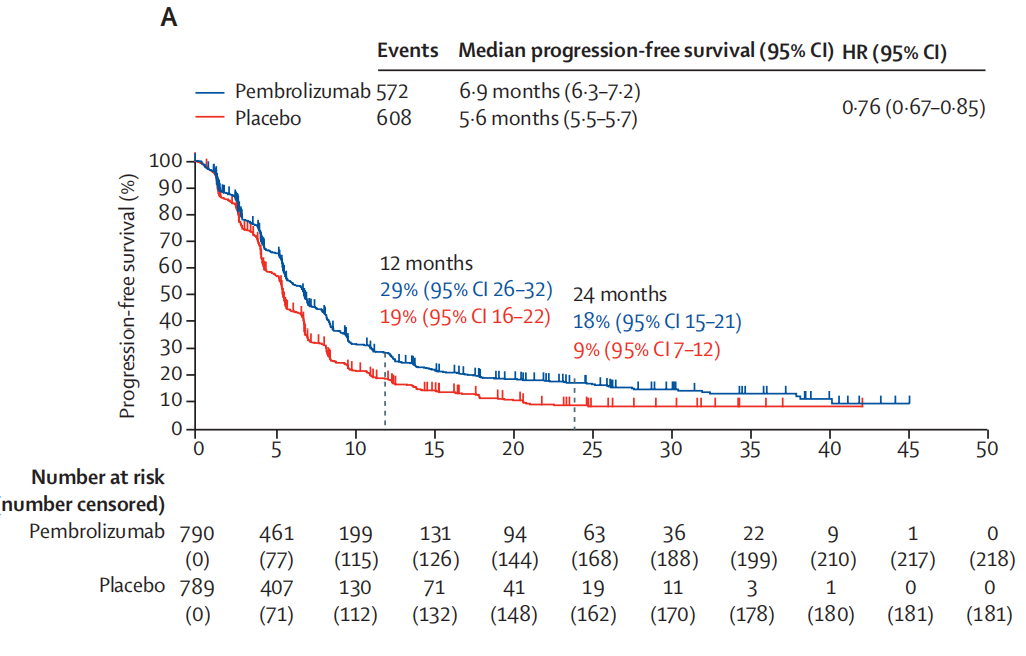 | 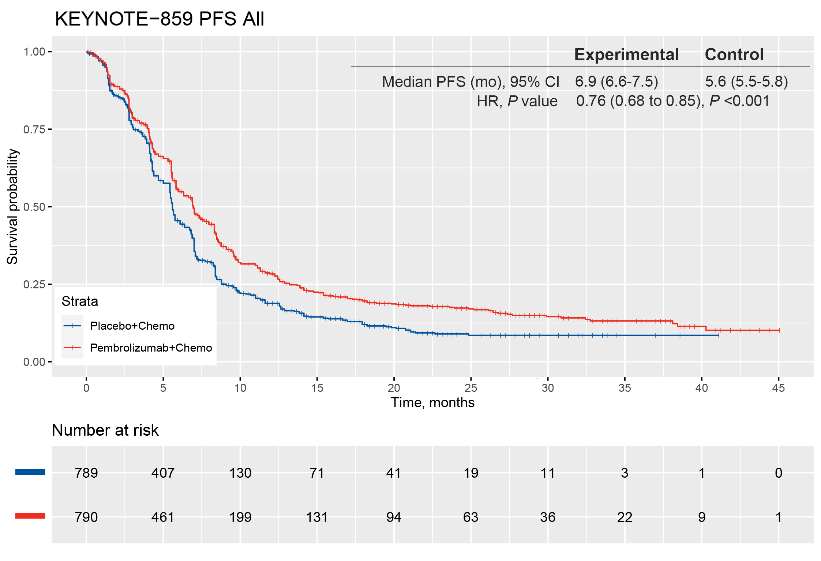 |
| **Study** | **Original figure** | **Reconstructed figure** |
| **KEYNOTE-859**  HER2 negative GEA  PFS  CPS>=1  PMID: 37875143  Figure 3B  Therapy: Pembrolizumab plus chemotherapy vs Placebo plus chemotherapy | 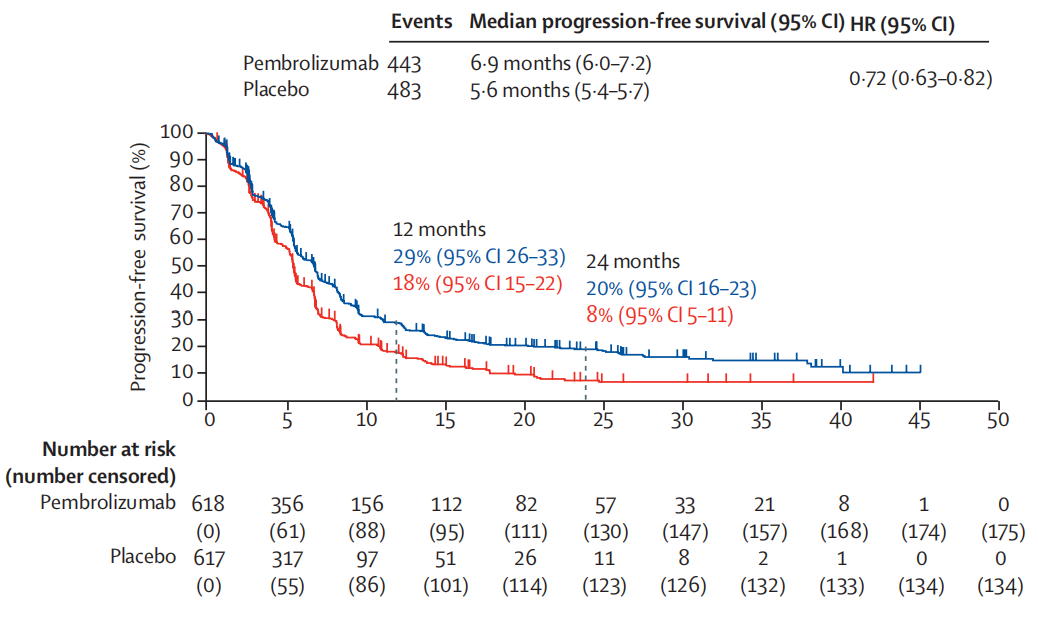 | 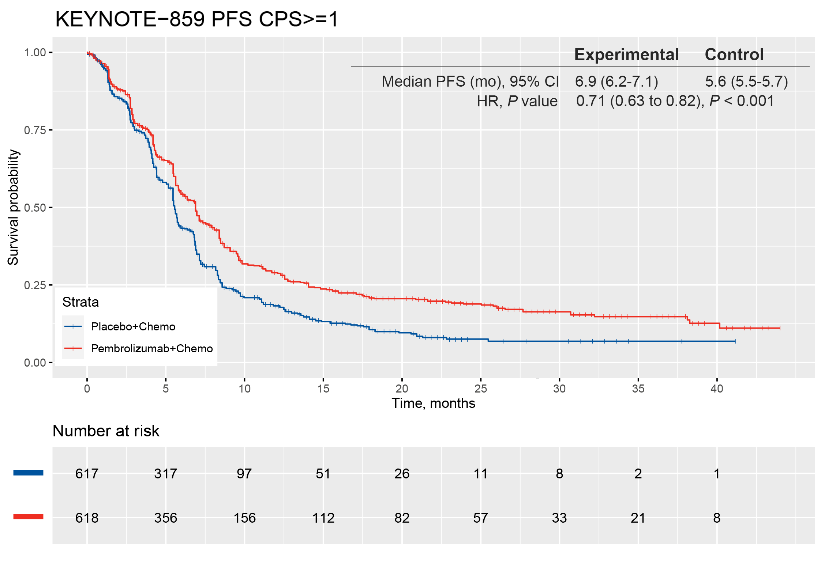 |
| **KEYNOTE-859**  HER2 negative GEA  PFS  CPS>=10  PMID: 37875143  Figure 3C  Therapy: Pembrolizumab plus chemotherapy vs Placebo plus chemotherapy | 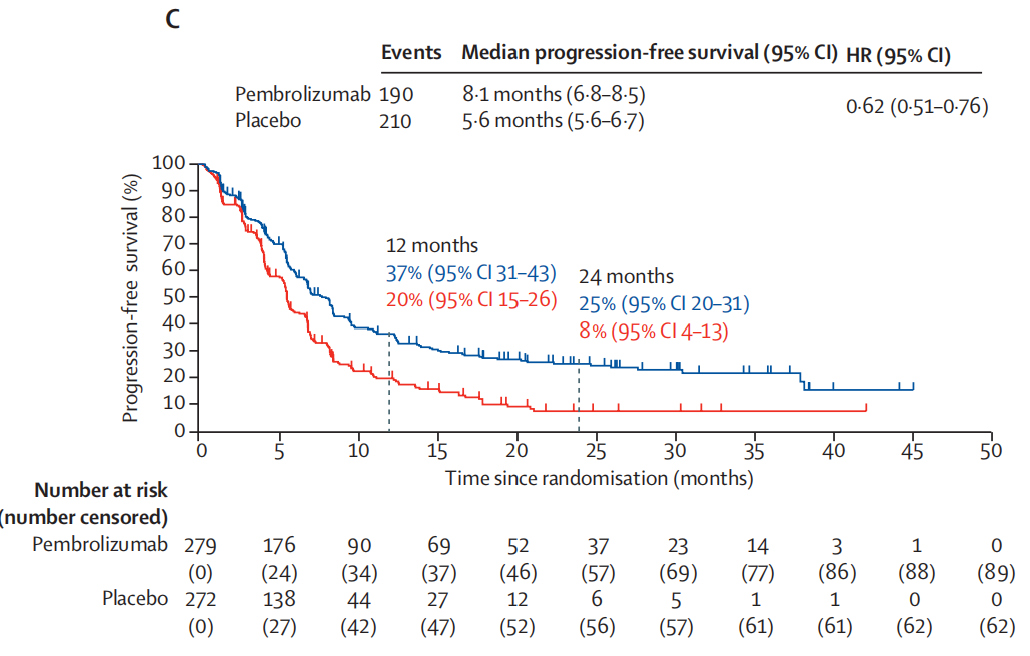 | 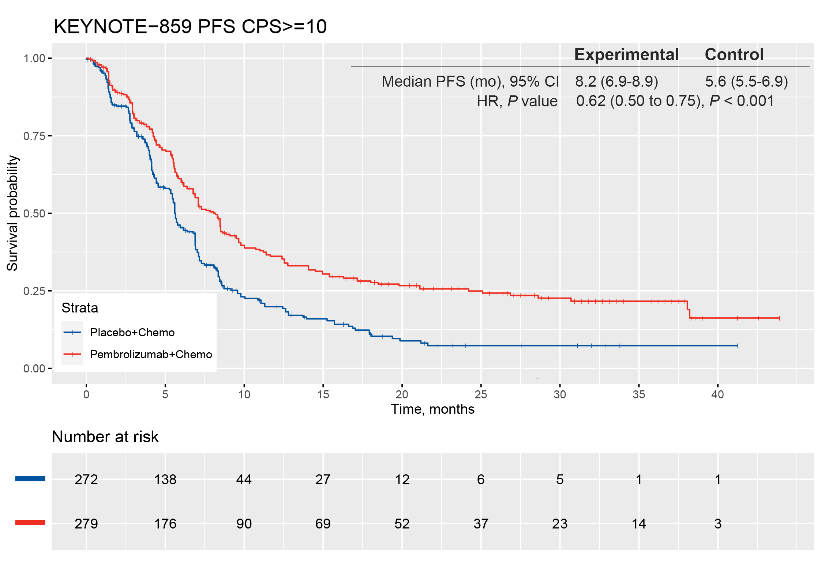 |
| **Study** | **Original figure** | **Reconstructed figure** |
| **KEYNOTE-062**  HER2 negative GEA  OS  CPS>=1  PMID: 32880601  Figure 2C  Therapy: Pembrolizumab plus chemotherapy vs placebo plus chemotherapy | 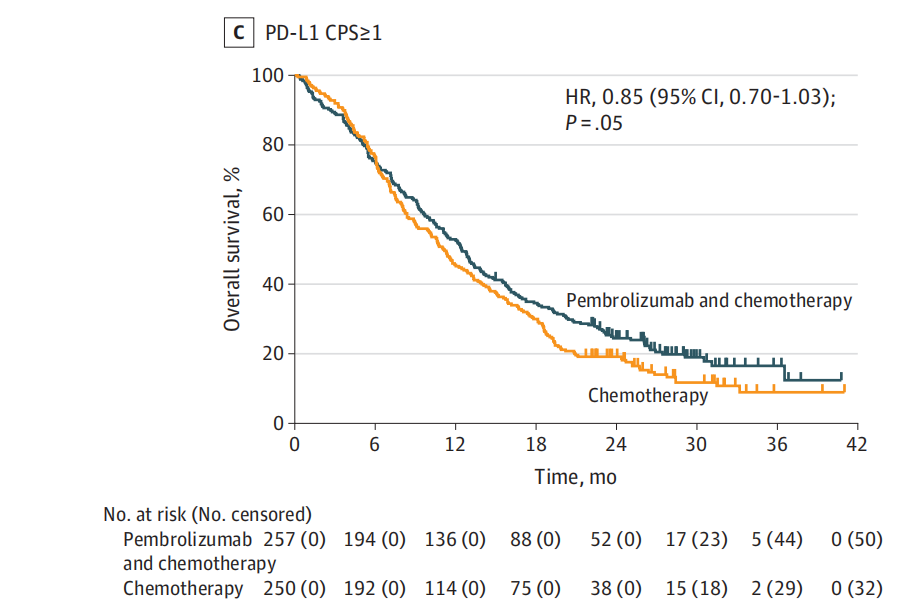 | 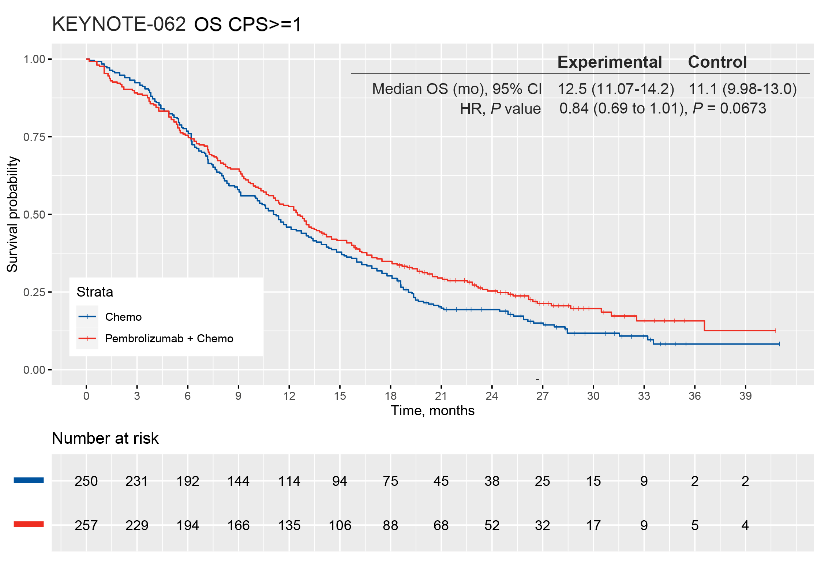 |
| **KEYNOTE-062**  HER2 negative GEA  OS  CPS>=10  PMID: 32880601  Figure 2D  Therapy: Pembrolizumab plus chemotherapy vs placebo plus chemotherapy | 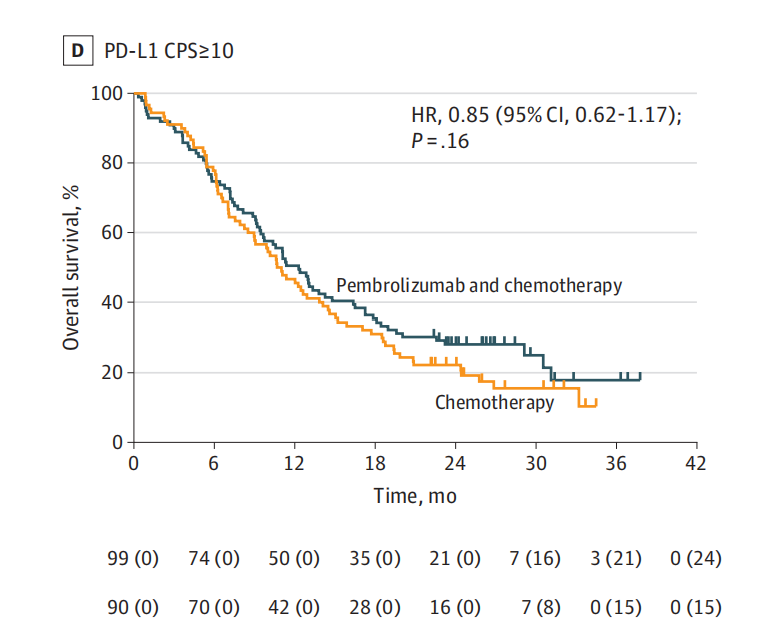 | 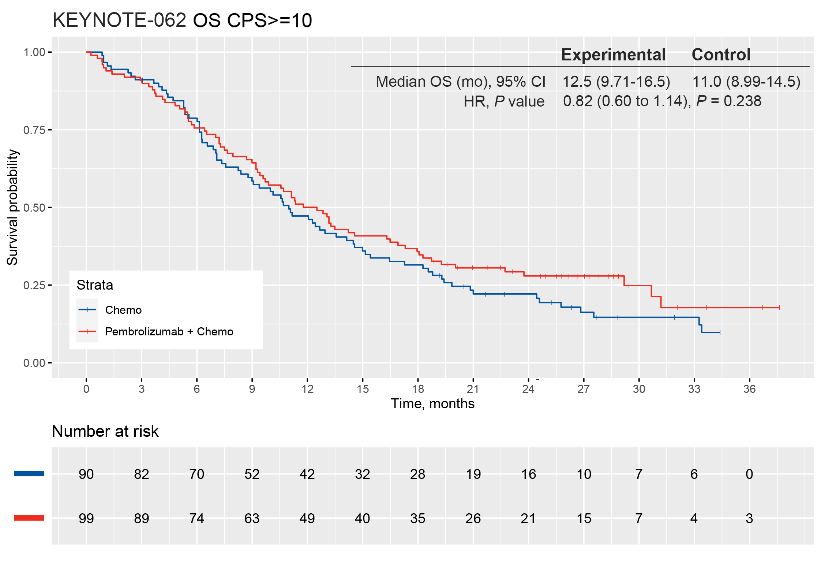 |
| **Study** | **Original figure** | **Reconstructed figure** |
| **KEYNOTE-062**  HER2 negative GEA  PFS  CPS>=1  PMID: 32880601  eFigure 4 C  Therapy: Pembrolizumab plus chemotherapy vs placebo plus chemotherapy | 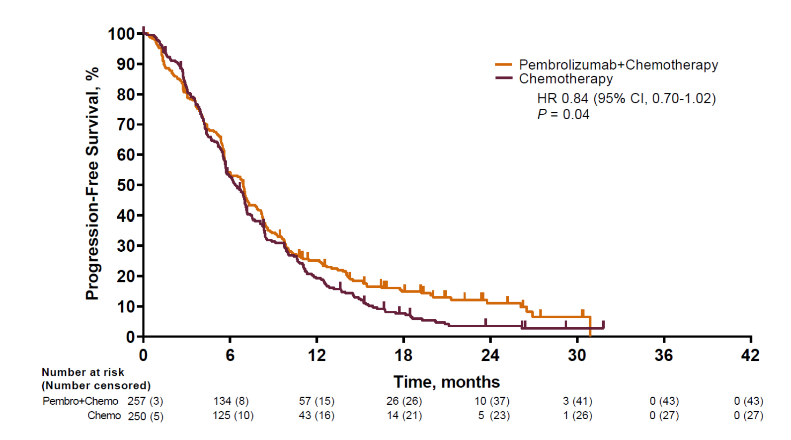 | 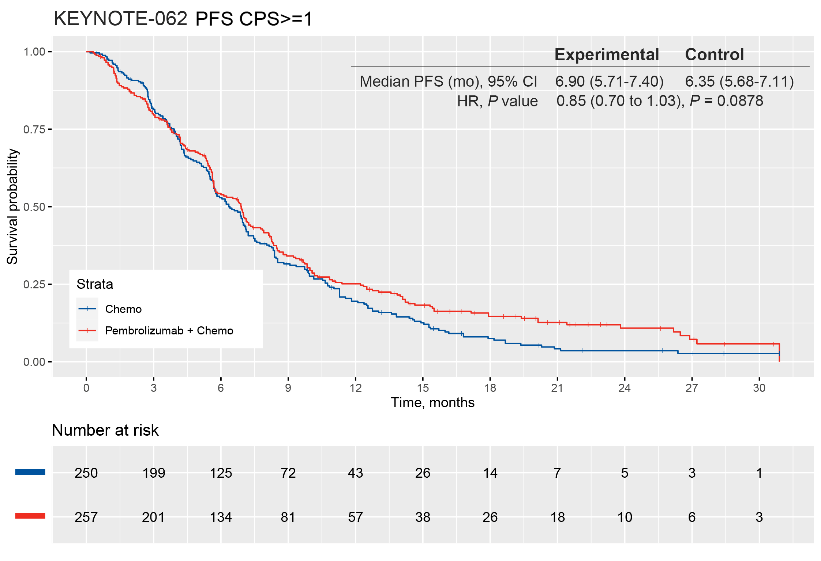 |
| **KEYNOTE-062**  HER2 negative GEA  PFS  CPS>=10  PMID: 32880601  eFigure 4D  Therapy: Pembrolizumab plus chemotherapy vs placebo plus chemotherapy | 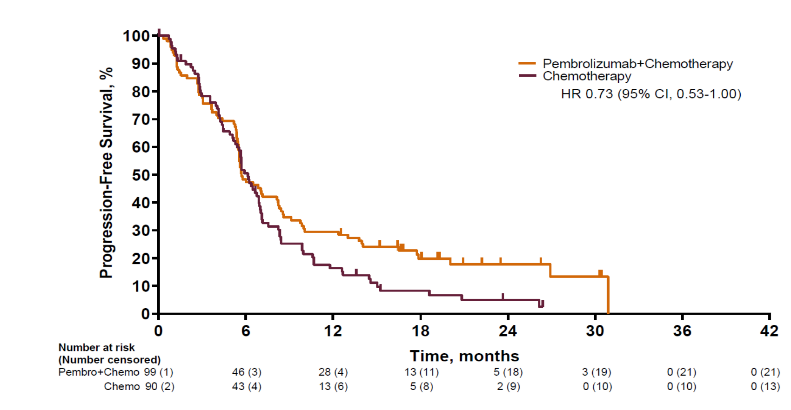 | 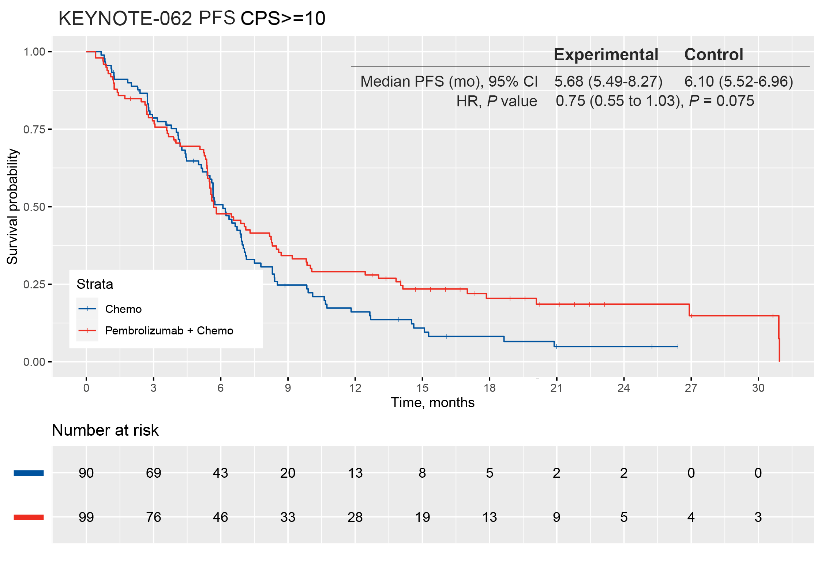 |
| **Study** | **Original figure** | **Reconstructed figure** |
| **ORIENT-16**  HER2 negative GEA  OS  Overall cohort  PMID: 38051328  Figure 2B  Therapy: Sintilimab plus chemotherapy vs Placebo plus chemotherapy | 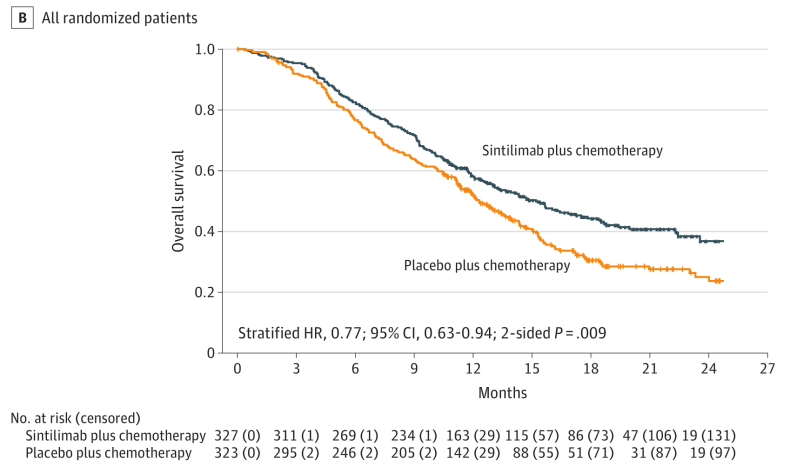 | 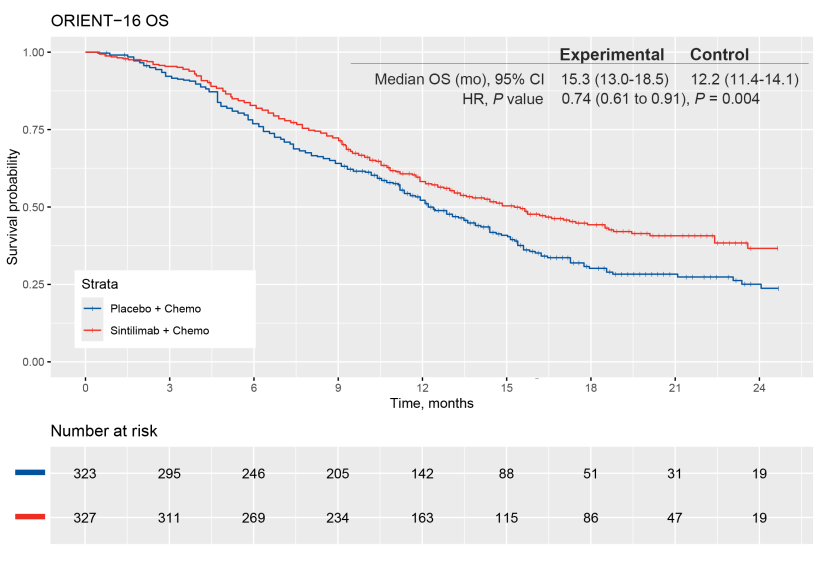 |
| **ORIENT16**  HER-2 negative GEA  OS  CPS>=5  PMID: 38051328  Figure 2B  Therapy: Sintilimab plus chemotherapy vs Placebo plus chemotherapy | 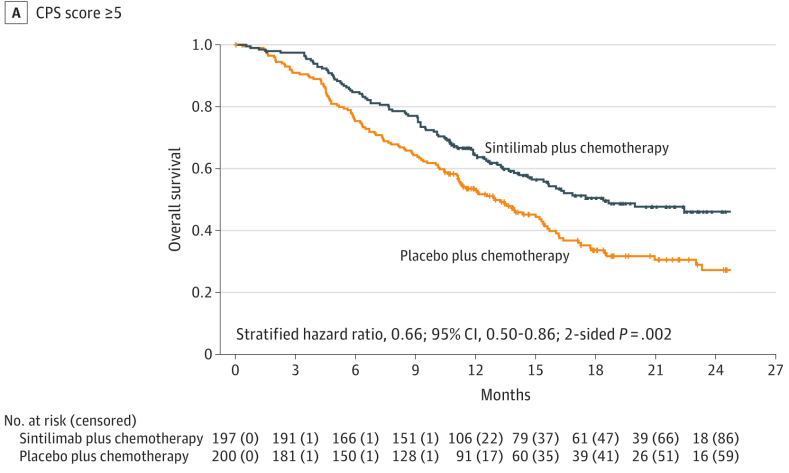 | 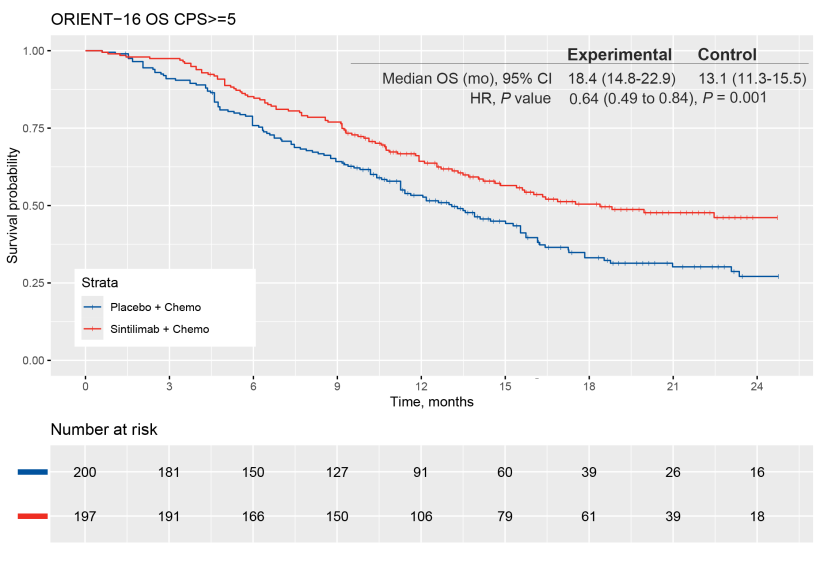 |
| **Study** | **Original figure** | **Reconstructed figure** |
| **ORIENT-16**  HER2 negative GEA  PFS  Overall cohort  PMID: 38051328  eFigure 1B  Therapy: Sintilimab plus chemotherapy vs Placebo plus chemotherapy | 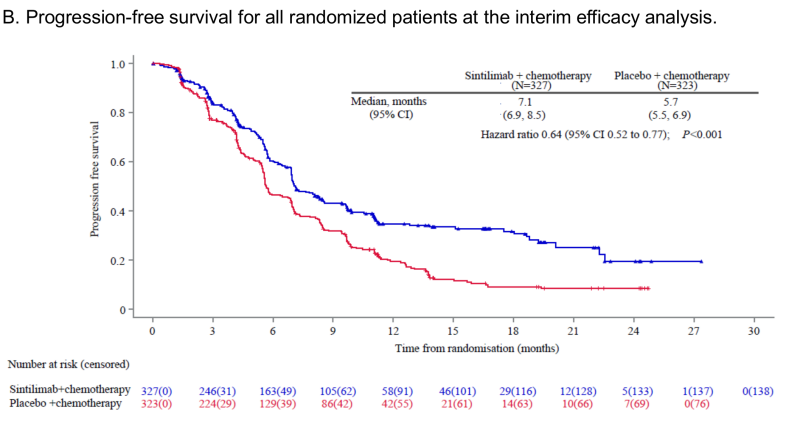 | 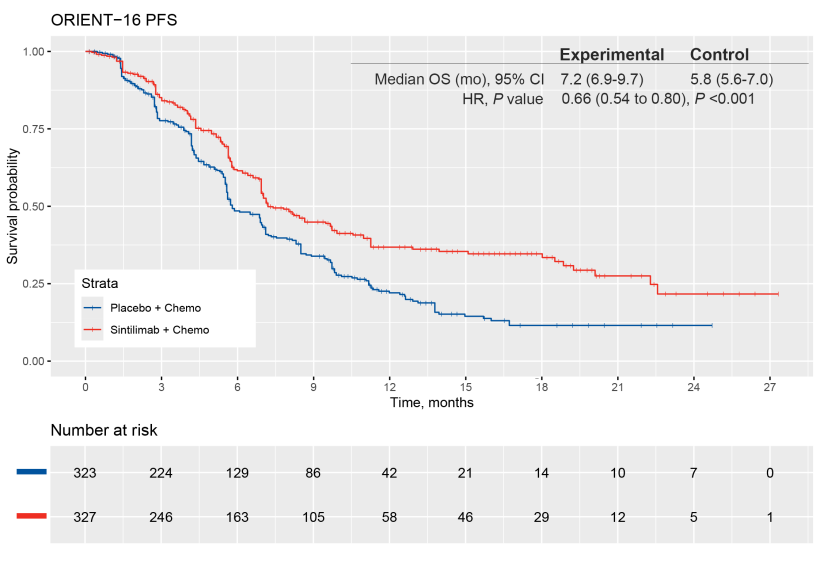 |
| **ORIENT-16**  HER-2 negative GEA  PFS  CPS>=5  PMID: 38051328  eFigure 1A  Therapy: Sintilimab plus chemotherapy vs Placebo plus chemotherapy | 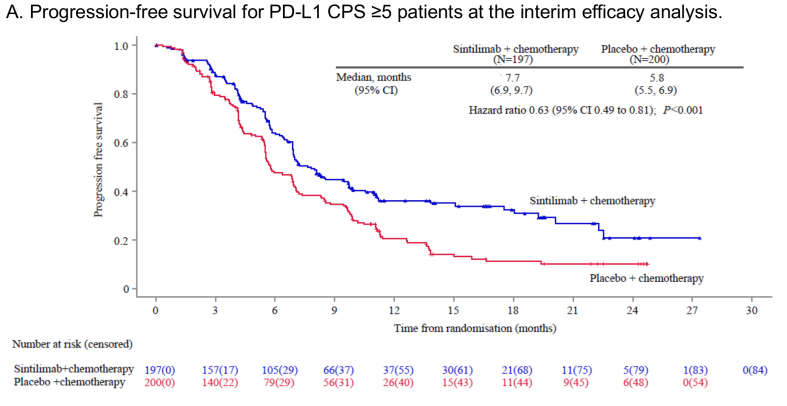 | 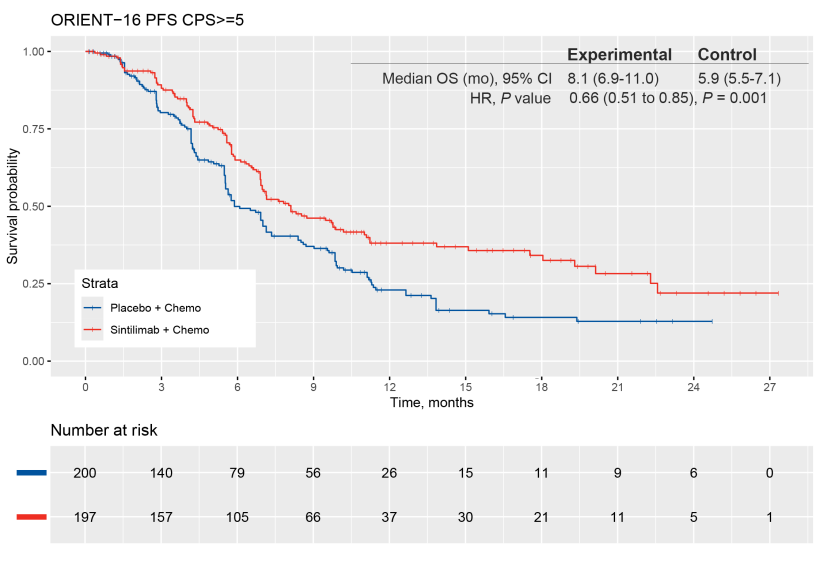 |
| **Study** | **Original figure** | **Reconstructed figure** |
| **RATIONALE-305**  HER2 negative GEA  OS  Overall cohort  (ESMO Congress 2023, LBA80)  Therapy: Tislelizumab plus chemotherapy vs Placebo plus chemotherapy | 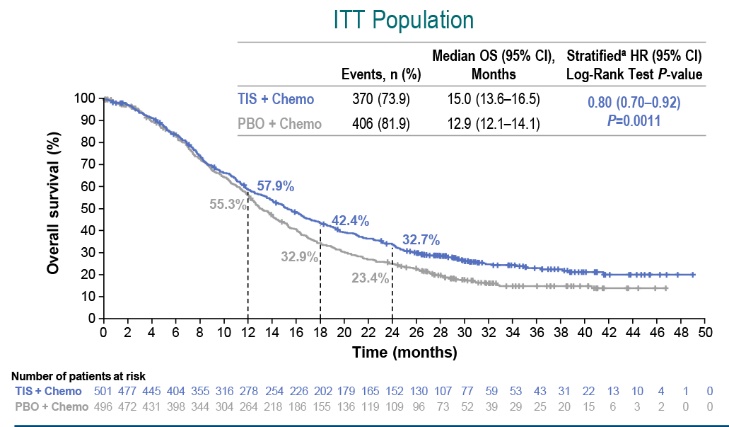 | 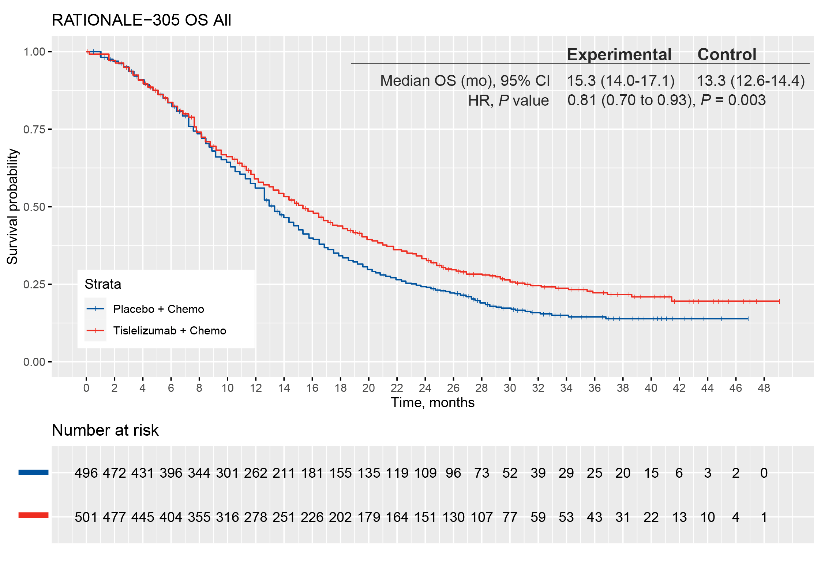 |

**Abbreviations**: HR, Hazard ratio; 95% CI, 95% of Confidence interval; OS, Overall survival; PFS, Progression free survival; TC, Tumor cell stain; IC, Immune cell stain; PD-L1, Programmed death-ligand 1; TPS, Tumor proportion Score; CPS, Combined positive score; TAP, Tumor area positivity; ESMO, European society of medical oncology; AACR, American association for cancer research.

# Figure S6. Reported hazard ratio (HR) for overall survival (OS) and progression free survival (PFS) in programmed death ligand (PD-L1) low subgroup using KMSubtraction workflow.

| **Study** | **PD-L1** | **Outcome** | **Reported HR** | **KMSubtraction with bipartite matching** |
| --- | --- | --- | --- | --- |
| **KEYNOTE-590**  Type: ESCC  Therapy: Pembrolizumab + Chemotherapy vs Chemotherapy | CPS<10 | OS | 0.99 (0.74-1.32) | 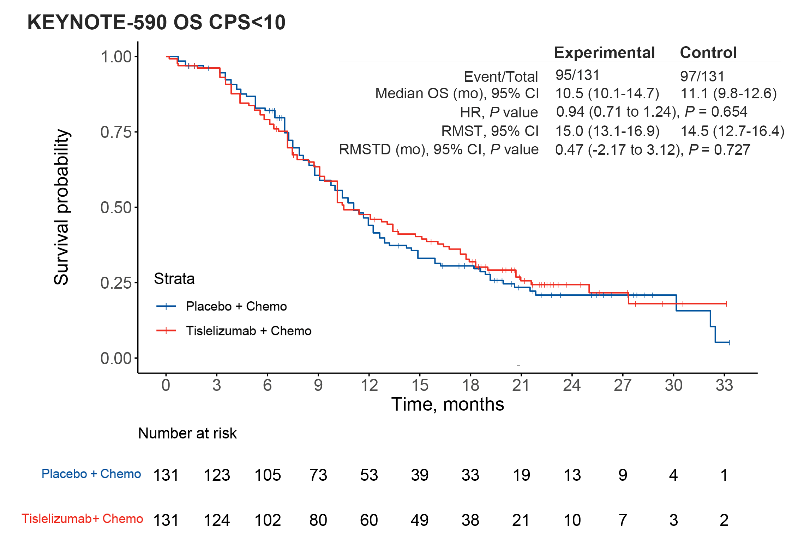 |
| **CheckMate-648**  Type: ESCC  Therapy: Nivolumab + Chemotherapy vs Chemotherapy | TPS <1% | OS | 0.98 (0.76, 1.28) | 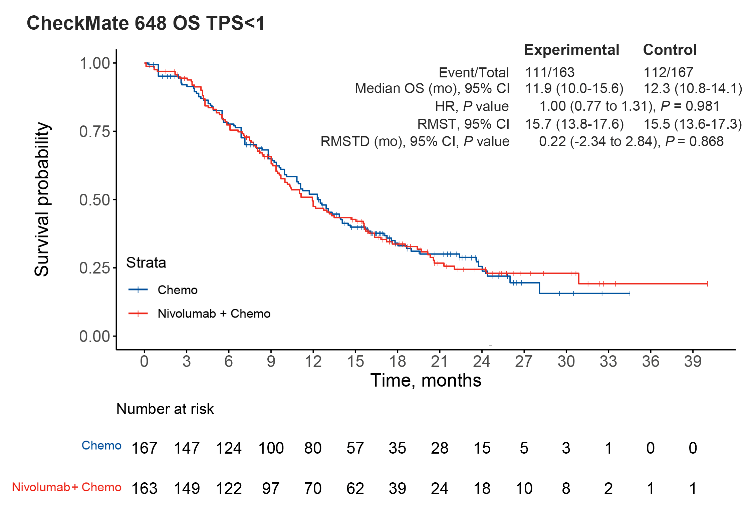 |
| **Study** | **PD-L1** | **Outcome** | **Reported HR** | **KMSubtraction with bipartite matching** |
| **CheckMate-648**  Type: ESCC  Therapy: Nivolumab + Ipilimumab vs Chemotherapy | TPS <1% | OS | 0.96 (0.74, 1.25) | 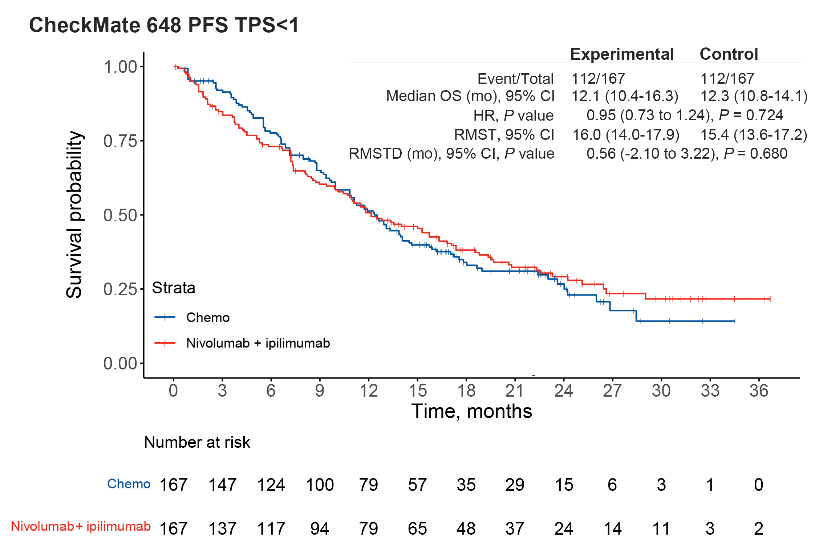 |
| **CheckMate-648**  Type: ESCC  Therapy: Nivolumab + Chemotherapy vs Chemotherapy | TPS <1% | PFS | 0.95 (0.73–1.24) | 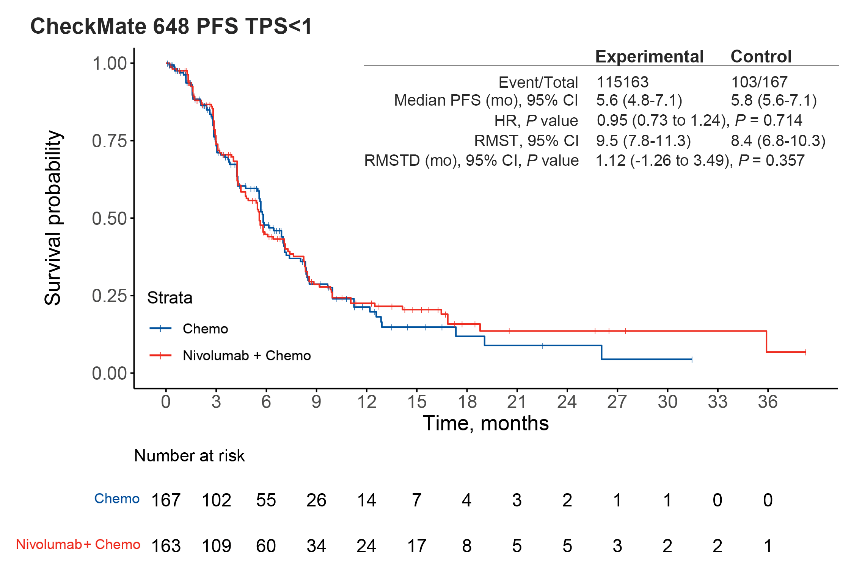 |
| Study | PD-L1 | Outcome | Reported HR | KMSubtraction with bipartite matching |
| **CheckMate-648**  Type: ESCC  Therapy: Nivolumab + Ipilimumab vs Chemotherapy | TPS <1% | PFS | 1.45 (1.13–1.88) | 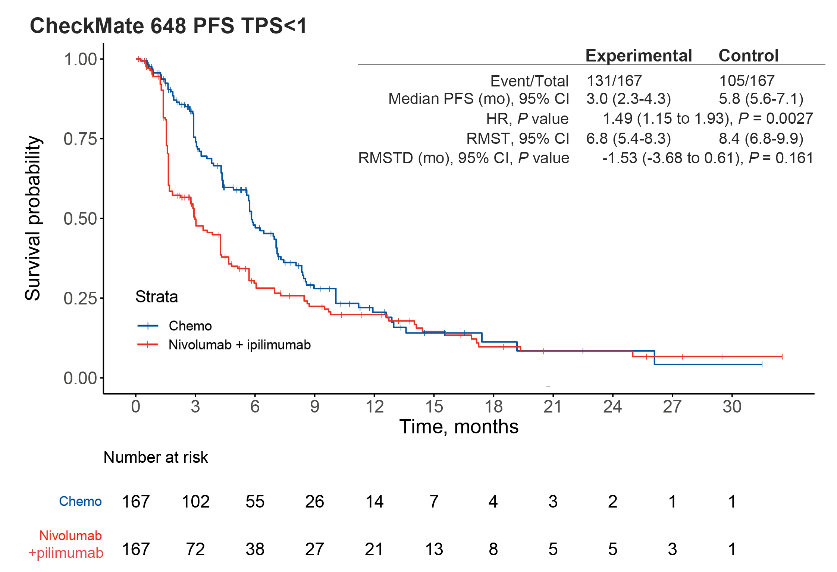 |
| **ORIENT-15**  Type: ESCC  Therapy: Sintilimab chemotherapy vs chemotherapy | CPS<10 | OS | 0.62 (0.45 to 0.85) | 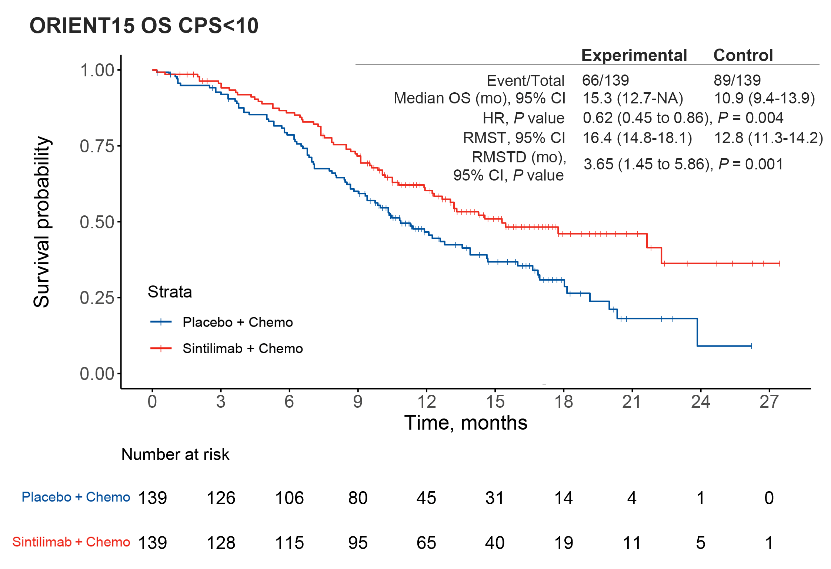 |
| Study | PD-L1 | Outcome | Reported HR | KMSubtraction with bipartite matching |
| **ORIENT-15**  Type: ESCC  Therapy: Sintilimab chemotherapy vs chemotherapy | CPS<10 | PFS | 0.53 (0.40 to 0.71) | 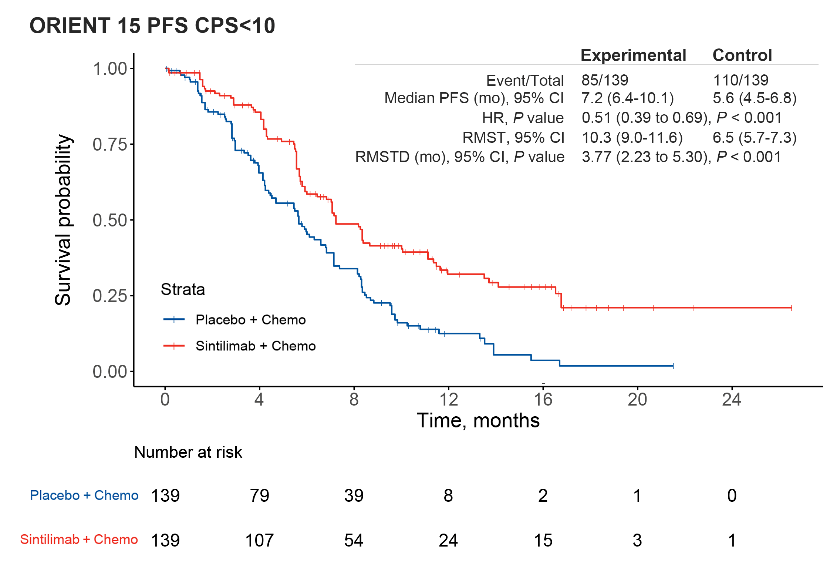 |
| **Checkmate-649**  Type: GEA  Therapy: Nivolumab +Chemotherapy vs Chemotherapy | CPS<1 | OS | 0.92 (0.70-1.23) | 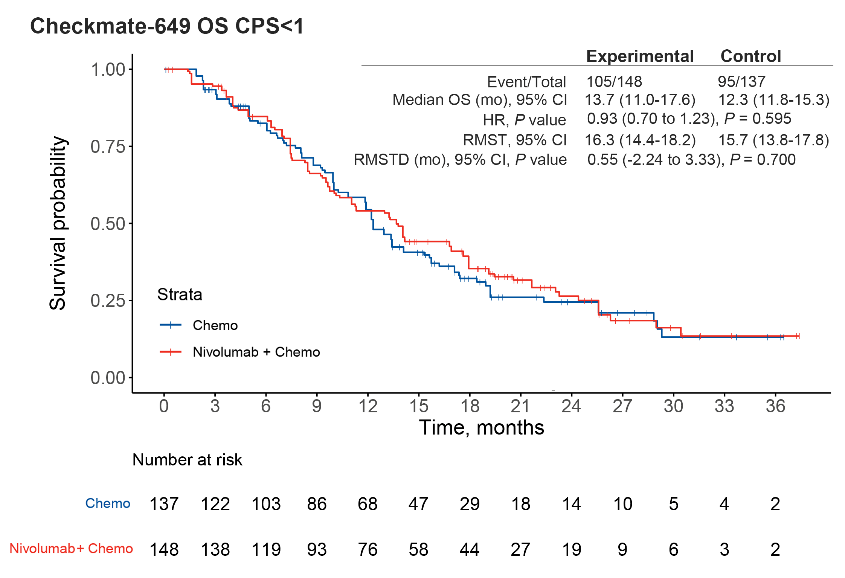 |
| Study | PD-L1 | Outcome | Reported HR | KMSubtraction with bipartite matching |
| **Checkmate-649**  Type: GEA  Therapy: Nivolumab +Chemotherapy vs Chemotherapy | CPS<5 | OS | 0.94 (0.78-1.13) | 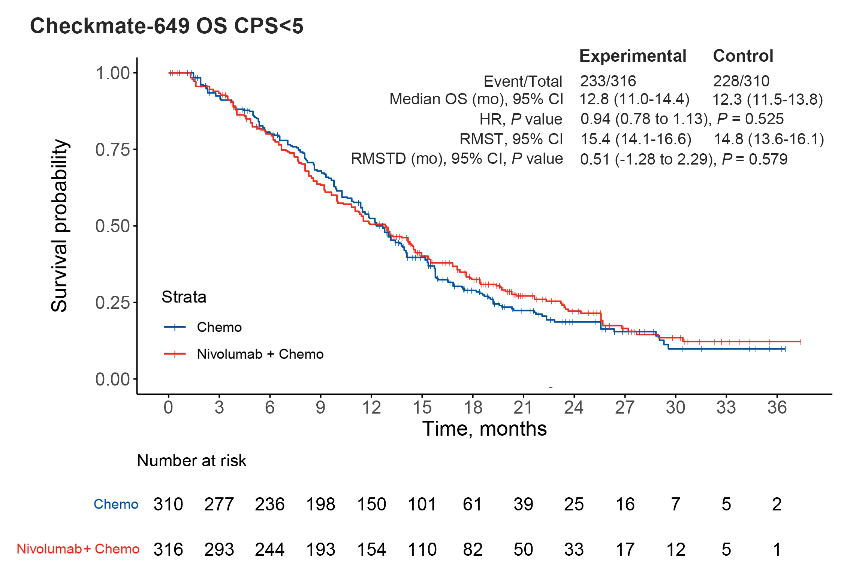 |
| **Checkmate-649**  Type: GEA  Therapy: Nivolumab +Chemotherapy vs Chemotherapy | CPS 1-4 | OS | - | 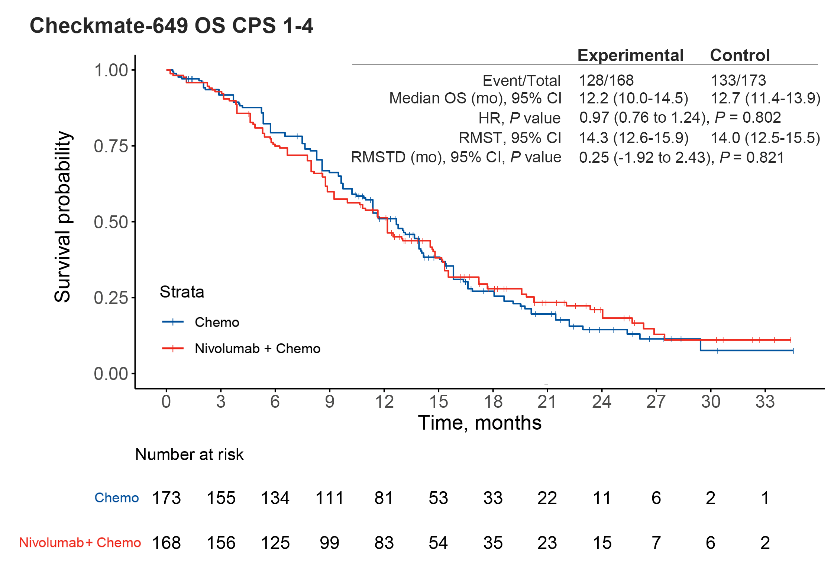 |
| Study | PD-L1 | Outcome | Reported HR | KMSubtraction with bipartite matching |
| **Checkmate-649**  Type: GEA  Therapy: Nivolumab +Chemotherapy vs Chemotherapy | CPS<1 | PFS | 0.93 (0.69-1.26) | 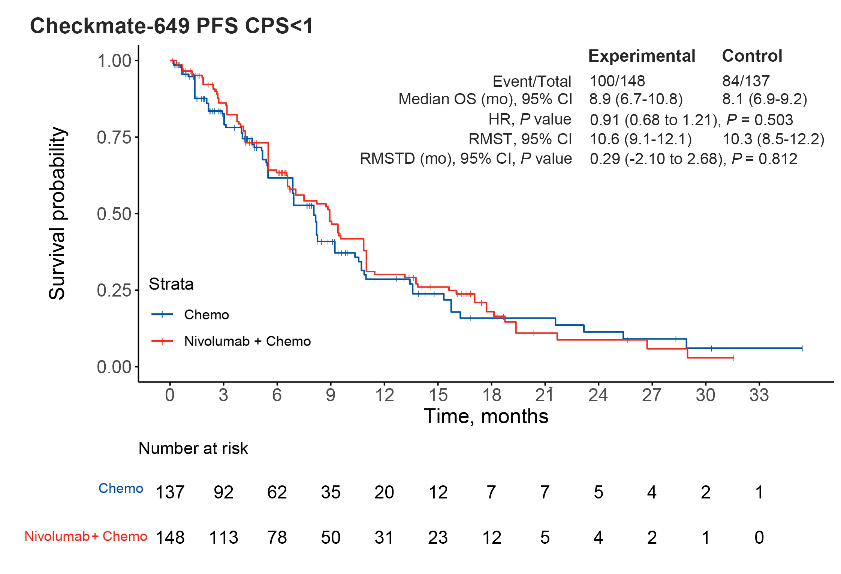 |
| **Checkmate-649**  Type: GEA  Therapy: Nivolumab +Chemotherapy vs Chemotherapy | CPS<5 | PFS | 0.93 (0.76-1.12) | 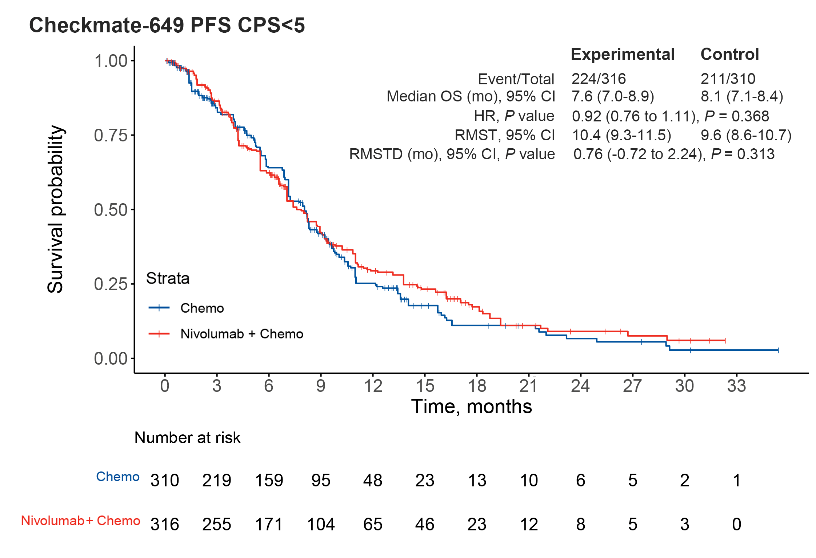 |
| Study | PD-L1 | Outcome | Reported HR | KMSubtraction with bipartite matching |
| **Checkmate-649**  Type: GEA  Therapy: Nivolumab +Chemotherapy vs Chemotherapy | CPS 1-4 | PFS | - | 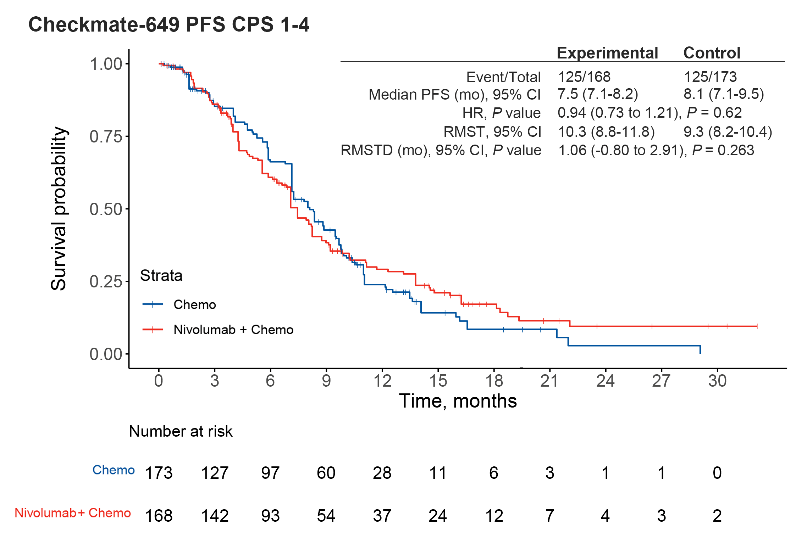 |
| **KEYNOTE-859**  Type: GEA  Therapy: Pembrolizumab + chemotherapy vs placebo +  chemotherapy | CPS<1 | OS | 0.92 (0.73–1.17) | 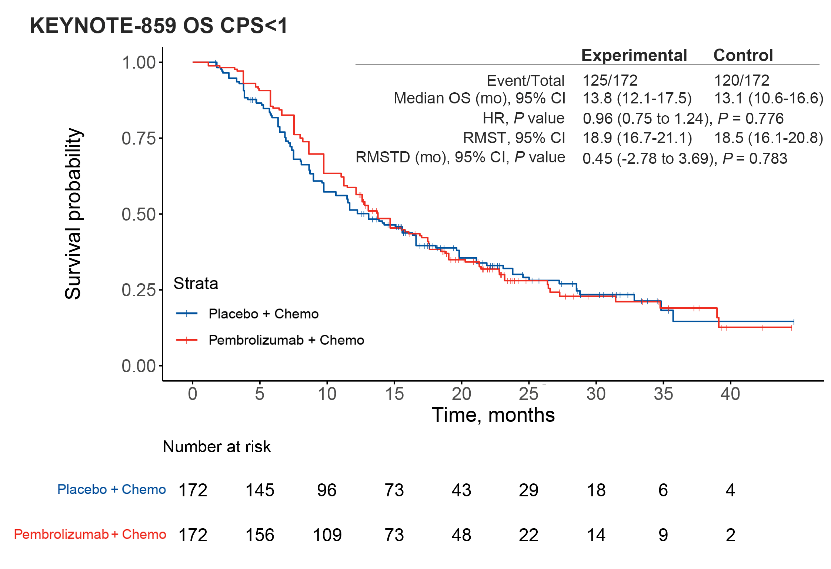 |
| Study | PD-L1 | Outcome | Reported HR | KMSubtraction with bipartite matching |
| **KEYNOTE-859**  Type: GEA  Therapy: Pembrolizumab + chemotherapy vs placebo +  chemotherapy | CPS<10 | OS | 0.86 (0.75–0.98) | 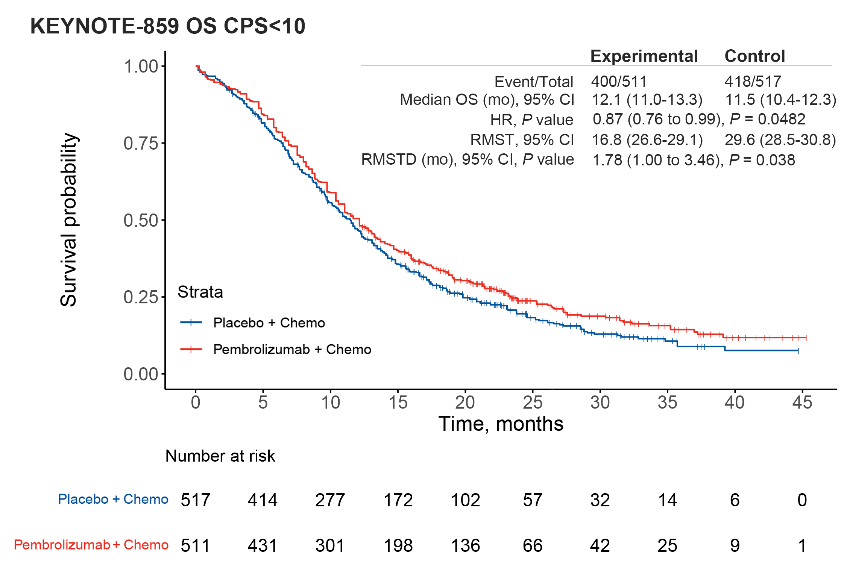 |
| **KEYNOTE-859**  Type: GEA  Therapy: Pembrolizumab + chemotherapy vs placebo +  chemotherapy | CPS 1-9 | OS | 0.83 (0.70–0.98) | 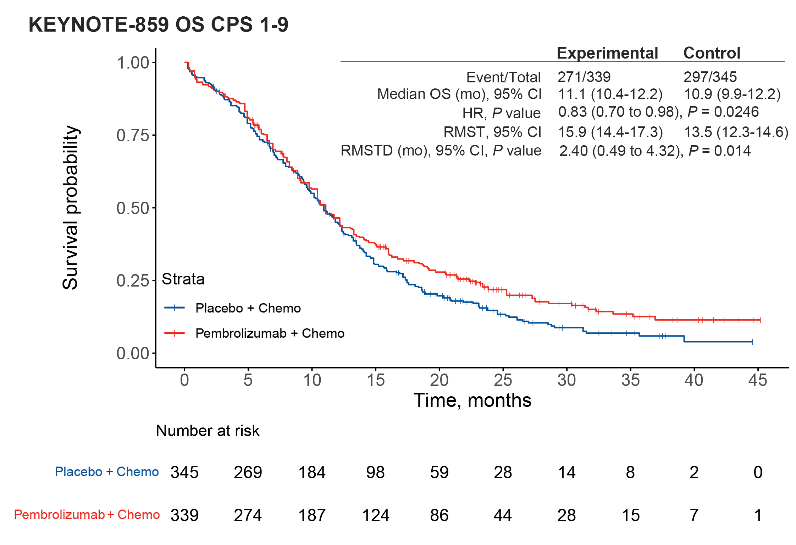 |
| Study | PD-L1 | Outcome | Reported HR | KMSubtraction with bipartite matching |
| **KEYNOTE-859**  Type: GEA  Therapy: Pembrolizumab + chemotherapy vs placebo +  chemotherapy | CPS<1 | PFS | 0.90 (0.70-1.15) | 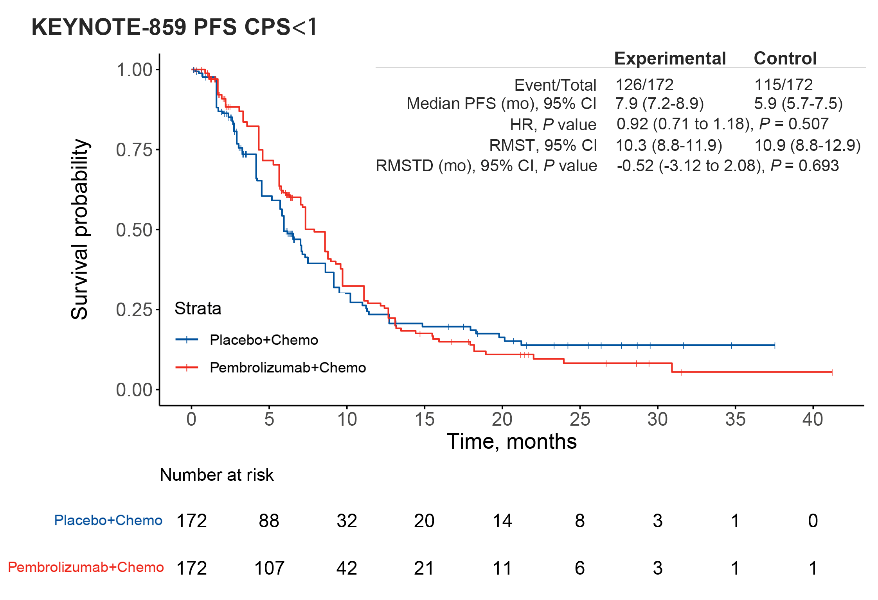 |
| **KEYNOTE-859**  Type: GEA  Therapy: Pembrolizumab + chemotherapy vs placebo +  chemotherapy | CPS<10 | PFS | 0.85 (0.74-0.98) | 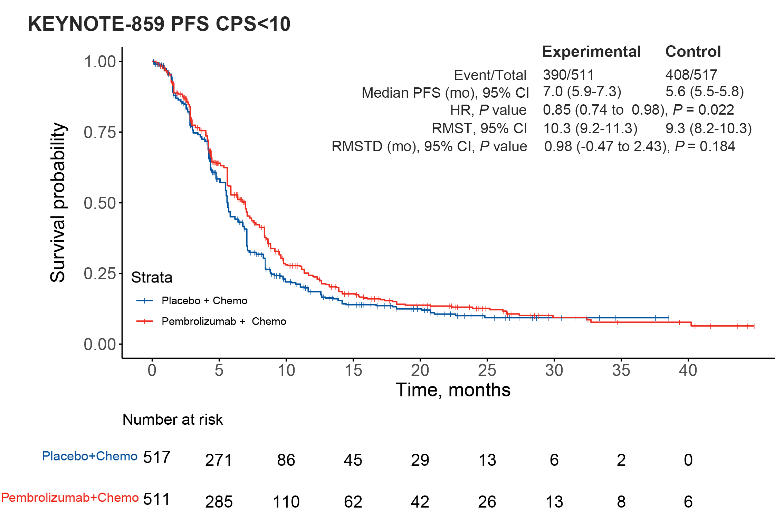 |
| Study | PD-L1 | Outcome | Reported HR | KMSubtraction with bipartite matching |
| **KEYNOTE-859**  Type: GEA  Therapy: Pembrolizumab + chemotherapy vs placebo +  chemotherapy | CPS 1-9 | PFS | 0.83 (0.70–0.99) | 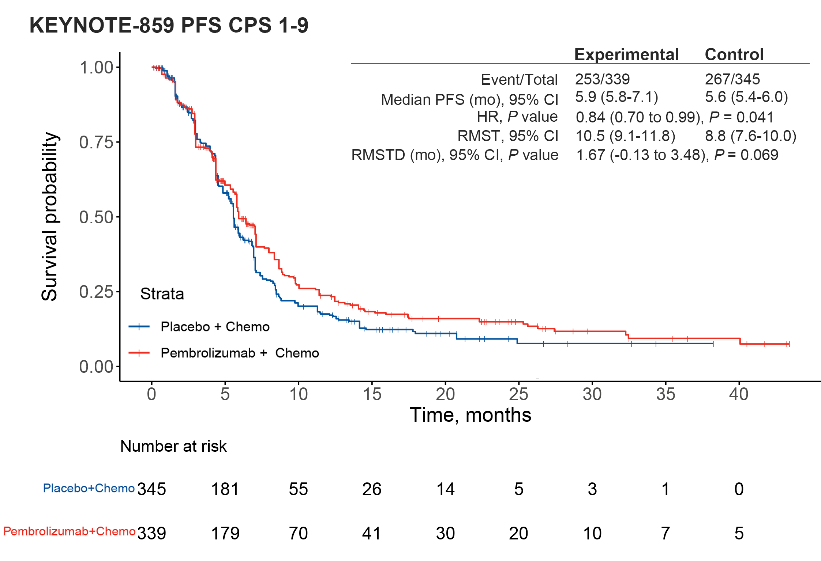 |
| **KEYNOTE-062**  Type: GEA  Therapy: Pembrolizumab + Chemo vs Chemo | CPS 1-9 | OS | - | 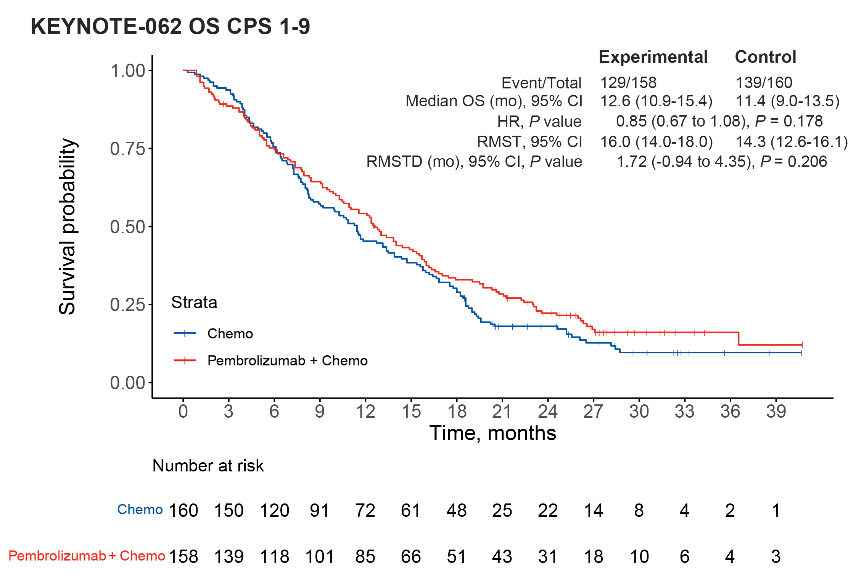 |
| Study | PD-L1 | Outcome | Reported HR | KMSubtraction with bipartite matching |
| **KEYNOTE-062**  Type: GEA  Therapy: Pembrolizumab + Chemo vs Chemo | CPS 1-9 | PFS | - | 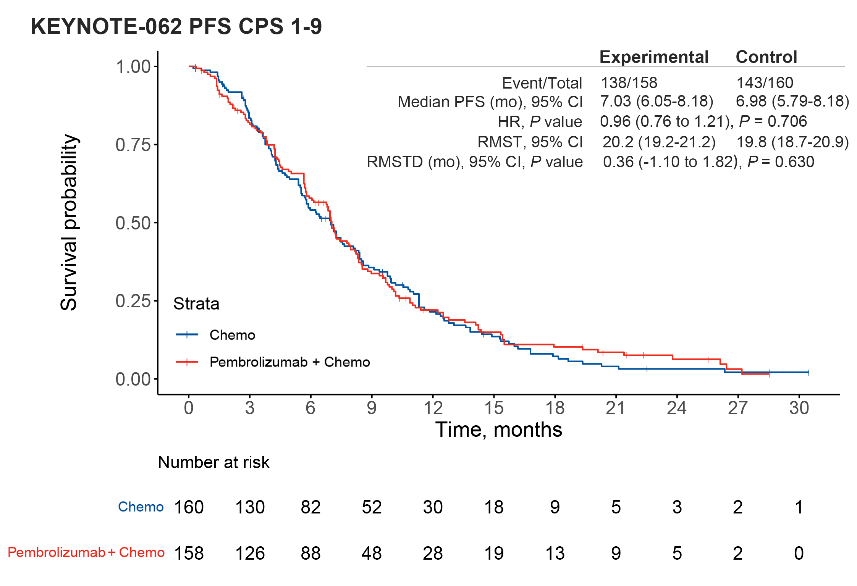 |
| **ORIENT-16**  Type: GEA  Therapy: Sintilimab + Chemotherapy vs Placebo + chemotherapy | CPS<5 | OS | 0.82 (0.63-1.08) | 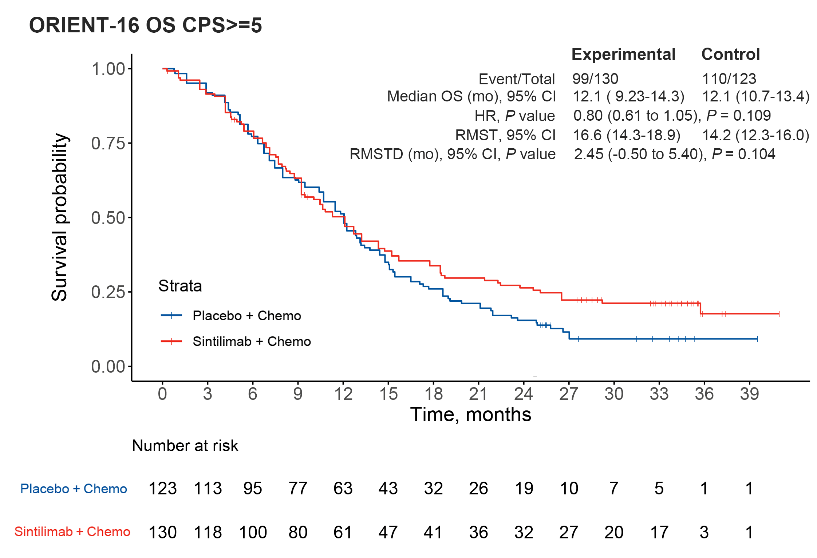 |
| **Study** | **PD-L1** | **Outcome** | **Reported HR** | **KMSubtraction with bipartite matching** |
| **ORIENT-16**  Type: GEA  Therapy: Sintilimab + Chemotherapy vs Placebo + chemotherapy | CPS<5 | PFS | XXXXXXXXXX |  |
| **RATIONALE-305**  Type: GEA  Therapy: | TAP<5 | OS | 0.91 (0.74-1.12) |  |

**Abbreviations:** ESCC, Esophageal squamous cell carcinoma; EXTREME, cetuximab plus cisplatin/carboplatin plus fluorouracil <= six cycles, then cetuximab maintenance; UC, Urothelial carcinoma; HNSCC, Head and neck squamous cell carcinoma; TNBC, Triple-negative breast cancer; CPS, Combined positive score; CI, Confidence interval; HR, Hazard ratio; PD-L1, Programmed death-ligand 1; PFS, Progression-free survival; TAP, Tumor area positivity; HR was based on a Cox regression model including treatment as a covariate, and pooled geographical region.

# Figure S7. Evaluation of KMSubtraction Bipartite Matching

| **KEYNOTE-590-sq OS CPS>=10 (Pembrolizumab +Chemo)** | | |
| --- | --- | --- |
| Empirical cumulative distribution between follow-up time of matched pairs and Bland-Altman plots to explore discrepancies between matched pairs | Kaplan Meier Curves and Cox-proportional hazard’s model comparing matched pairs | |
|  | | |
| **KEYNOTE-590-sq** **OS CPS>=10 (Chemotherapy)** | | |
| Empirical cumulative distribution between follow-up time of matched pairs and Bland-Altman plots to explore discrepancies between matched pairs | | Kaplan Meier Curves and Cox-proportional hazard’s model comparing matched pairs |
|  | | |
| **CHECKMATE-648 OS TPS>=1% (Nivolumab + Chemotherapy)** | |  |
| Empirical cumulative distribution between follow-up time of matched pairs and Bland-Altman plots to explore discrepancies between matched pairs | | Kaplan Meier Curves and Cox-proportional hazard’s model comparing matched pairs |
|  | | |
| **CHECKMATE-648 OS TPS>=1% (Nivolumab + Ipilimumab)** | |  |
| Empirical cumulative distribution between follow-up time of matched pairs and Bland-Altman plots to explore discrepancies between matched pairs | | Kaplan Meier Curves and Cox-proportional hazard’s model comparing matched pairs |
|  | | |
| **CHECKMATE-648 OS TPS>=1% (Chemotherapy)** | |  |
| Empirical cumulative distribution between follow-up time of matched pairs and Bland-Altman plots to explore discrepancies between matched pairs | | Kaplan Meier Curves and Cox-proportional hazard’s model comparing matched pairs |
|  | | |
| **CHECKMATE-648 PFS TPS>=1% (Nivolumab + Chemotherapy)** | |  |
| Empirical cumulative distribution between follow-up time of matched pairs and Bland-Altman plots to explore discrepancies between matched pairs | | Kaplan Meier Curves and Cox-proportional hazard’s model comparing matched pairs |
|  | | |
| **CHECKMATE-648 PFS TPS>=1% (Nivolumab + Ipilimumab)** | |  |
| Empirical cumulative distribution between follow-up time of matched pairs and Bland-Altman plots to explore discrepancies between matched pairs | | Kaplan Meier Curves and Cox-proportional hazard’s model comparing matched pairs |
|  | | |
| **CHECKMATE-648 PFS TPS>=1% (Chemotherapy)** | |  |
| Empirical cumulative distribution between follow-up time of matched pairs and Bland-Altman plots to explore discrepancies between matched pairs | | Kaplan Meier Curves and Cox-proportional hazard’s model comparing matched pairs |
|  | | |
| **ORIENT-15 OS CPS>=10 (Sintilimab + Chemotherapy)** | |  |
| Empirical cumulative distribution between follow-up time of matched pairs and Bland-Altman plots to explore discrepancies between matched pairs | | Kaplan Meier Curves and Cox-proportional hazard’s model comparing matched pairs |
|  | | |
| **ORIENT-15 OS CPS>=10 (Chemotherapy)** | |  |
| Empirical cumulative distribution between follow-up time of matched pairs and Bland-Altman plots to explore discrepancies between matched pairs | | Kaplan Meier Curves and Cox-proportional hazard’s model comparing matched pairs |
|  | | |
| **ORIENT-15 PFS CPS>=10 (Sintilimab + Chemotherapy)** | |  |
| Empirical cumulative distribution between follow-up time of matched pairs and Bland-Altman plots to explore discrepancies between matched pairs | | Kaplan Meier Curves and Cox-proportional hazard’s model comparing matched pairs |
|  | | |
| **ORIENT-15 PFS CPS>=10 (Chemotherapy)** | |  |
| Empirical cumulative distribution between follow-up time of matched pairs and Bland-Altman plots to explore discrepancies between matched pairs | | Kaplan Meier Curves and Cox-proportional hazard’s model comparing matched pairs |
|  | | |
| **Checkmate-649 OS CPS>=1 (Nivolumab + Chemotherapy)** | |  |
| Empirical cumulative distribution between follow-up time of matched pairs and Bland-Altman plots to explore discrepancies between matched pairs | | Kaplan Meier Curves and Cox-proportional hazard’s model comparing matched pairs |
|  | | |
| **Checkmate-649 OS CPS>=1 (Chemotherapy)** | |  |
| Empirical cumulative distribution between follow-up time of matched pairs and Bland-Altman plots to explore discrepancies between matched pairs | | Kaplan Meier Curves and Cox-proportional hazard’s model comparing matched pairs |
|  | | |
| **Checkmate-649 OS CPS>=5 (Nivolumab + Chemotherapy)** | |  |
| Empirical cumulative distribution between follow-up time of matched pairs and Bland-Altman plots to explore discrepancies between matched pairs | | Kaplan Meier Curves and Cox-proportional hazard’s model comparing matched pairs |
|  | | |
| **Checkmate-649 OS CPS>=5 (Chemotherapy)** | |  |
| Empirical cumulative distribution between follow-up time of matched pairs and Bland-Altman plots to explore discrepancies between matched pairs | | Kaplan Meier Curves and Cox-proportional hazard’s model comparing matched pairs |
|  | | |
| **Checkmate-649 OS CPS 1-4 (Nivolumab + Chemotherapy)** | |  |
| Empirical cumulative distribution between follow-up time of matched pairs and Bland-Altman plots to explore discrepancies between matched pairs | | Kaplan Meier Curves and Cox-proportional hazard’s model comparing matched pairs |
|  | | |
| **Checkmate-649 OS CPS 1-4 (Chemotherapy)** | |  |
| Empirical cumulative distribution between follow-up time of matched pairs and Bland-Altman plots to explore discrepancies between matched pairs | | Kaplan Meier Curves and Cox-proportional hazard’s model comparing matched pairs |
|  | | |
| **Checkmate-649 PFS CPS>=1 (Nivolumab + Chemotherapy)** | |  |
| Empirical cumulative distribution between follow-up time of matched pairs and Bland-Altman plots to explore discrepancies between matched pairs | | Kaplan Meier Curves and Cox-proportional hazard’s model comparing matched pairs |
|  | | |
| **Checkmate-649 PFS CPS>=1 (Chemotherapy)** | |  |
| Empirical cumulative distribution between follow-up time of matched pairs and Bland-Altman plots to explore discrepancies between matched pairs | | Kaplan Meier Curves and Cox-proportional hazard’s model comparing matched pairs |
|  | | |
| **Checkmate-649 PFS CPS>=5 (Nivolumab + Chemotherapy)** | |  |
| Empirical cumulative distribution between follow-up time of matched pairs and Bland-Altman plots to explore discrepancies between matched pairs | | Kaplan Meier Curves and Cox-proportional hazard’s model comparing matched pairs |
|  | | |
| **Checkmate-649 PFS CPS>=5 (Chemotherapy)** | |  |
| Empirical cumulative distribution between follow-up time of matched pairs and Bland-Altman plots to explore discrepancies between matched pairs | | Kaplan Meier Curves and Cox-proportional hazard’s model comparing matched pairs |
|  | | |
| **Checkmate-649 PFS CPS 1-4 (Nivolumab + Chemotherapy)** | |  |
| Empirical cumulative distribution between follow-up time of matched pairs and Bland-Altman plots to explore discrepancies between matched pairs | | Kaplan Meier Curves and Cox-proportional hazard’s model comparing matched pairs |
|  | | |
| **Checkmate-649 PFS CPS 1-4 (Chemotherapy)** | |  |
| Empirical cumulative distribution between follow-up time of matched pairs and Bland-Altman plots to explore discrepancies between matched pairs | | Kaplan Meier Curves and Cox-proportional hazard’s model comparing matched pairs |
|  | | |
| **KEYNOTE-859 OS CPS>=1 (Pembrolizumab + Chemotherapy)** | |  |
| Empirical cumulative distribution between follow-up time of matched pairs and Bland-Altman plots to explore discrepancies between matched pairs | | Kaplan Meier Curves and Cox-proportional hazard’s model comparing matched pairs |
|  | | |
| **KEYNOTE-859 OS CPS>=1 (Placebo + Chemotherapy)** | |  |
| Empirical cumulative distribution between follow-up time of matched pairs and Bland-Altman plots to explore discrepancies between matched pairs | | Kaplan Meier Curves and Cox-proportional hazard’s model comparing matched pairs |
|  | | |
| **KEYNOTE-859 OS CPS>=10 (Pembrolizumab + Chemotherapy)** | |  |
| Empirical cumulative distribution between follow-up time of matched pairs and Bland-Altman plots to explore discrepancies between matched pairs | | Kaplan Meier Curves and Cox-proportional hazard’s model comparing matched pairs |
|  | | |
| **KEYNOTE-859 OS CPS>=10 (Placebo + Chemotherapy)** | |  |
| Empirical cumulative distribution between follow-up time of matched pairs and Bland-Altman plots to explore discrepancies between matched pairs | | Kaplan Meier Curves and Cox-proportional hazard’s model comparing matched pairs |
|  | | |
| **KEYNOTE-859 OS CPS 1-9 (Pembrolizumab + Chemotherapy)** | |  |
| Empirical cumulative distribution between follow-up time of matched pairs and Bland-Altman plots to explore discrepancies between matched pairs | | Kaplan Meier Curves and Cox-proportional hazard’s model comparing matched pairs |
|  | | |
| **KEYNOTE-859 OS CPS 1-9 (Placebo + Chemotherapy)** | |  |
| Empirical cumulative distribution between follow-up time of matched pairs and Bland-Altman plots to explore discrepancies between matched pairs | | Kaplan Meier Curves and Cox-proportional hazard’s model comparing matched pairs |
|  | | |
| **KEYNOTE-859 PFS CPS>=1 (Pembrolizumab + Chemotherapy)** | |  |
| Empirical cumulative distribution between follow-up time of matched pairs and Bland-Altman plots to explore discrepancies between matched pairs | | Kaplan Meier Curves and Cox-proportional hazard’s model comparing matched pairs |
|  | | |
| **KEYNOTE-859 PFS CPS>=1 (Placebo + Chemotherapy)** | |  |
| Empirical cumulative distribution between follow-up time of matched pairs and Bland-Altman plots to explore discrepancies between matched pairs | | Kaplan Meier Curves and Cox-proportional hazard’s model comparing matched pairs |
|  | | |
| **KEYNOTE-859 PFS CPS>=10 (Pembrolizumab + Chemotherapy)** | |  |
| Empirical cumulative distribution between follow-up time of matched pairs and Bland-Altman plots to explore discrepancies between matched pairs | | Kaplan Meier Curves and Cox-proportional hazard’s model comparing matched pairs |
|  | | |
| **KEYNOTE-859 PFS CPS>=10 (Placebo + Chemotherapy)** | |  |
| Empirical cumulative distribution between follow-up time of matched pairs and Bland-Altman plots to explore discrepancies between matched pairs | | Kaplan Meier Curves and Cox-proportional hazard’s model comparing matched pairs |
|  | | |
| **KEYNOTE-859 PFS CPS 1-9 (Pembrolizumab + Chemotherapy)** | |  |
| Empirical cumulative distribution between follow-up time of matched pairs and Bland-Altman plots to explore discrepancies between matched pairs | | Kaplan Meier Curves and Cox-proportional hazard’s model comparing matched pairs |
|  | | |
| **KEYNOTE-859 PFS CPS 1-9 (Placebo + Chemotherapy)** | |  |
| Empirical cumulative distribution between follow-up time of matched pairs and Bland-Altman plots to explore discrepancies between matched pairs | | Kaplan Meier Curves and Cox-proportional hazard’s model comparing matched pairs |
|  | | |
| **KEYNOTE-062 OS CPS>=10 (Pembrolizumab + Chemotherapy)** | | |
| Empirical cumulative distribution between follow-up time of matched pairs and Bland-Altman plots to explore discrepancies between matched pairs | | Kaplan Meier Curves and Cox-proportional hazard’s model comparing matched pairs |
|  | | |
| **KEYNOTE-062 OS CPS>=10 (Chemotherapy)** | | |
| Empirical cumulative distribution between follow-up time of matched pairs and Bland-Altman plots to explore discrepancies between matched pairs | | Kaplan Meier Curves and Cox-proportional hazard’s model comparing matched pairs |
|  | | |
| **KEYNOTE-062 PFS CPS>=10 (Pembrolizumab+ Chemotherapy)** | | |
| Empirical cumulative distribution between follow-up time of matched pairs and Bland-Altman plots to explore discrepancies between matched pairs | | Kaplan Meier Curves and Cox-proportional hazard’s model comparing matched pairs |
|  | | |
| **KEYNOTE-062 PFS CPS>=10 (Chemotherapy)** | | |
| Empirical cumulative distribution between follow-up time of matched pairs and Bland-Altman plots to explore discrepancies between matched pairs | | Kaplan Meier Curves and Cox-proportional hazard’s model comparing matched pairs |
|  | | |
| **ORIENT-16 OS CPS>=5 (Sintilimab + Chemotherapy)** | | |
| Empirical cumulative distribution between follow-up time of matched pairs and Bland-Altman plots to explore discrepancies between matched pairs | | Kaplan Meier Curves and Cox-proportional hazard’s model comparing matched pairs |
|  | | |
| **ORIENT-16 OS CPS>=5 (Placebo + Chemotherapy)** | | |
| Empirical cumulative distribution between follow-up time of matched pairs and Bland-Altman plots to explore discrepancies between matched pairs | | Kaplan Meier Curves and Cox-proportional hazard’s model comparing matched pairs |
|  | | |
| **ORIENT-16 PFS CPS>=5 (Sintilimab + Chemotherapy)** | | |
| Empirical cumulative distribution between follow-up time of matched pairs and Bland-Altman plots to explore discrepancies between matched pairs | | Kaplan Meier Curves and Cox-proportional hazard’s model comparing matched pairs |
|  | | |
| **ORIENT-16 PFS CPS>=5 (Placebo + Chemotherapy)** | | |
| Empirical cumulative distribution between follow-up time of matched pairs and Bland-Altman plots to explore discrepancies between matched pairs | | Kaplan Meier Curves and Cox-proportional hazard’s model comparing matched pairs |
|  | | |
| **RATIONALE-305 OS TAP>=5% (Tislelizumab + Chemotherapy)** | | |
| Empirical cumulative distribution between follow-up time of matched pairs and Bland-Altman plots to explore discrepancies between matched pairs | | Kaplan Meier Curves and Cox-proportional hazard’s model comparing matched pairs |
|  | | |
| **RATIONALE-305 OS TAP>=5% (Placebo + Chemotherapy)** | | |
| Empirical cumulative distribution between follow-up time of matched pairs and Bland-Altman plots to explore discrepancies between matched pairs | | Kaplan Meier Curves and Cox-proportional hazard’s model comparing matched pairs |
|  | | |

# Figure S8. Convergence plots and histograms of simulations

| **KEYNOTE-590-sq OS (Pembrolizumab + Chemotherapy)** | | |
| --- | --- | --- |
|  |  |  |
| **KEYNOTE-590-sq OS (Chemotherapy)** | | |
|  |  |  |
| **Checkmate-648 OS Nivolumab + Chemotherapy** | | |
|  |  |  |
| **Checkmate-648 OS Nivolumab + Ipilimumab** | | |
|  |  |  |
| **Checkmate-648 OS Chemotherapy** | | |
|  |  |  |
| **Checkmate-648 PFS Nivolumab + Chemotherapy** | | |
|  |  |  |
| **Checkmate-648 PFS Nivolumab + Ipilimumab** | | |
|  |  |  |
| **Checkmate-648 PFS Chemotherapy** | | |
|  |  |  |
| **ORIENT-15 OS Sintilimab+ Chemotherapy All/CPS>=10** | | |
|  |  |  |
| **ORIENT-15 OS Chemotherapy All/CPS>=10** | | |
|  |  |  |
| **ORIENT-15 PFS Sintilimab+ Chemotherapy All/CPS>=10** | | |
|  |  |  |
| **ORIENT-15 PFS Chemotherapy All/CPS>=10** | | |
|  |  |  |
| **KEYNOTE-859 OS Pembrolizumab + Chemotherapy All/CPS>=1** | | |
|  |  |  |
| **KEYNOTE-859 OS Placebo + Chemotherapy All/CPS>=1** | | |
|  |  |  |
| **KEYNOTE-859 OS Pembrolizumab plus chemotherapy All/CPS>=10** | | |
|  |  |  |
| **KEYNOTE-859 OS Placebo + Chemotherapy All/CPS>=10** | | |
|  |  |  |
| **KEYNOTE-859 OS Pembrolizumab plus chemotherapy CPS>=10/>=1** | | |
|  |  |  |
| **KEYNOTE-859 OS Chemotherapy CPS>=10/>=1** | | |
|  |  |  |
| **KEYNOTE-859 PFS Pembrolizumab + Chemotherapy All/CPS>=1** | | |
|  |  |  |
| **KEYNOTE-859 PFS Chemotherapy All/CPS>=1** | | |
|  |  |  |
| **KEYNOTE-859 PFS Pembrolizumab plus chemotherapy All/CPS>=10** | | |
|  |  |  |
| **KEYNOTE-859 PFS Chemotherapy All/CPS>=10** | | |
|  |  |  |
| **KEYNOTE-859 PFS Pembrolizumab plus chemotherapy CPS>=10/>=1** | | |
|  |  |  |
| **KEYNOTE-859 PFS Chemotherapy CPS>=10/>=1** | | |
|  |  |  |
| **KEYNOTE-062 OS CPS>=10 Pembrolizumab + Chemotherapy** | | |
|  |  |  |
| **KEYNOTE-062 OS CPS>=10 Chemotherapy** | | |
|  |  |  |
| **KEYNOTE-062 PFS CPS>=10 Pembrolizumab + Chemotherapy** | | |
|  |  |  |
| **KEYNOTE-062 PFS CPS>=10 Chemotherapy** | | |
|  |  |  |
| **ORIENT-16 OS CPS>=5 Sintilimab + Chemotherapy** | | |
|  |  |  |
| **ORIENT-16 OS CPS>=5 Placebo + Chemotherapy** | | |
|  |  |  |
| **ORIENT-16 PFS CPS>=5 Sintilimab + Chemotherapy** | | |
|  |  |  |
| **ORIENT-16 PFS CPS>=5 Placebo + Chemotherapy** | | |
|  |  |  |
| **RATIONALE-305 OS Tislelizumab + Chemotherapy** | | |
|  |  |  |
| **RATIONALE-305 OS Placebo + Chemotherapy** | | |
|  |  |  |
